# *Aspergillus niger* membrane-associated proteome analysis for the identification of glucose transporters

**DOI:** 10.1186/s13068-015-0317-9

**Published:** 2015-09-17

**Authors:** J. Sloothaak, D. I. Odoni, L. H. de Graaff, V. A. P. Martins dos Santos, P. J. Schaap, J. A. Tamayo-Ramos

**Affiliations:** Laboratory of Systems and Synthetic Biology, Wageningen University, Stippeneng 4, 6708 WE Wageningen, The Netherlands

**Keywords:** *Aspergillus niger*, Membrane-associated proteome, Shotgun proteomics, Hidden Markov models, HMM_gluT_, Transportome, Glucose transporters, MstG, MstH

## Abstract

**Background:**

The development of biological processes that replace the existing petrochemical-based industry is one of the biggest challenges in biotechnology. *Aspergillus niger* is one of the main industrial producers of lignocellulolytic enzymes, which are used in the conversion of lignocellulosic feedstocks into fermentable sugars. Both the hydrolytic enzymes responsible for lignocellulose depolymerisation and the molecular mechanisms controlling their expression have been well described, but little is known about the transport systems for sugar uptake in *A. niger*. Understanding the transportome of *A. niger* is essential to achieve further improvements at strain and process design level. Therefore, this study aims to identify and classify *A. niger* sugar transporters, using newly developed tools for in silico and in vivo analysis of its membrane-associated proteome.

**Results:**

In the present research work, a hidden Markov model (HMM), that shows a good performance in the identification and segmentation of functionally validated glucose transporters, was constructed. The model (HMM_gluT_) was used to analyse the *A. niger* membrane-associated proteome response to high and low glucose concentrations at a low pH. By combining the abundance patterns of the proteins found in the *A. niger* plasmalemma proteome with their HMM_gluT_ scores, two new putative high-affinity glucose transporters, denoted MstG and MstH, were identified. MstG and MstH were functionally validated and biochemically characterised by heterologous expression in a *S. cerevisiae* glucose transport null mutant. They were shown to be a high-affinity glucose transporter (*K*_m_ = 0.5 ± 0.04 mM) and a very high-affinity glucose transporter (*K*_m_ = 0.06 ± 0.005 mM), respectively.

**Conclusions:**

This study, focusing for the first time on the membrane-associated proteome of the industrially relevant organism *A. niger*, shows the global response of the transportome to the availability of different glucose concentrations. Analysis of the *A. niger* transportome with the newly developed HMM_gluT_ showed to be an efficient approach for the identification and classification of new glucose transporters.

**Electronic supplementary material:**

The online version of this article (doi:10.1186/s13068-015-0317-9) contains supplementary material, which is available to authorized users.

## Background

The development of biological systems for the industrial synthesis of biofuels and chemicals is a main objective of today’s biotechnology. For cost-effective production of valuable products, efficient microbial fermentations of non-food lignocellulosic material are essential. Filamentous fungi, in particular the fungus *Aspergillus niger*, play a prominent role in this field of biotechnology. *A. niger* is of significant industrial relevance and has been exploited as a production platform for both organic acids and hydrolytic enzymes [1]. It is an efficient degrader of the major plant cell wall polysaccharides cellulose, hemicellulose and pectin [2], and is one of the main industrial producers of commercial enzymes for plant biomass conversion due to its high enzyme secretory capacity [3]. In the last decades, its versatile arsenal of extracellular enzymes for lignocellulose depolymerisation [4], and the molecular mechanisms controlling their expression, have been well described [5–7]. However, while previous studies have revealed the existence of an array of uptake systems in this fungus [8–10], little is known about the identity and specificity of the transport systems involved in sugar uptake.

Most of the existing knowledge related to monosaccharide uptake in fungi originates from studies in the model yeast *Saccharomyces cerevisiae*. This yeast is able to transport and metabolise glucose, fructose, mannose and galactose. Transport of these simple sugars is mediated only through facilitated diffusion by the majority of the transporters from the Hxt family, composed of Hxt1–Hxt17, and Gal2. They belong to the sugar porter (SP) family [11], which is the largest subfamily of the major facilitator superfamily (MFS). Hxt1–Hxt4, Hxt6 and Hxt7 have been found to be able to support growth of yeast in glucose on their own, thus being considered the major hexose transporters in yeast. In addition to the Hxt family, three members of the maltose transporter family (Agt1, Mph2 and Mph3) are also able to transport glucose [12]. The individual characterisation of each of these transporters was possible using engineered *S. cerevisiae* strains, deleted for *hxt1*–*7* [13] and *hxt1*–*17*, *gal2*, *agt1*, *mph2* and *mph 3* [12], as hosts for the functional validation and biochemical study of these proteins. These yeast mutant strains, unable to grow on glucose, fructose, mannose and galactose as a single carbon source, have also subsequently been used as tools for the functional characterisation of sugar transporters from other fungal species [14–21].

In contrast to *S. cerevisiae*, the only functionally validated sugar transporters in *A. niger* are the recently identified d-galacturonic acid transporter GatA [22, 23], two fructose transporters [10] and the high-affinity sugar/H^+^ symporter MstA [14]. Furthermore, transcriptional data for the *A. nigermstC* gene suggests that it encodes a low-affinity glucose transporter [9], but no experimental data supporting its role as a functional sugar transporter is publicly available. MFS proteins display a strong structural conservation [24], and structure-based profile hidden Markov models can be used to identify putative sugar transporters in the *A. niger* in silico proteome. To obtain a profile hidden Markov model (HMM), a multiple sequence alignment is turned into a position-dependent scoring system with segments of variable conservation levels and length [25]. As a result of the weighted scoring system, HMMs are less sensitive to changes in the non-conserved regions of a given protein family than more traditional methods based on shared primary sequence similarity alone, like e.g. the standard Blast algorithm. These changes include variability of the residues at a certain position as well as insertions and deletions. In this study, a profile hidden Markov model specific for glucose transporters (HMM_gluT_) was computed based on a structure-based multiple sequence alignment of 42 proteins with a known function related to glucose transport.

Genome information combined with transcriptome analyses of various growth conditions can give a good inventory of *A. niger* plasma membrane components with hypothetical sugar transporter functions. These data types, however, by definition do not take regulatory events at the posttranscriptional level into account, although these events can influence protein abundances and localisation. An inventory of the *A. niger* plasma membrane proteome at defined culture conditions can provide a more reliable source of information for the identification of the most important sugar transport components. To date, only few fungal plasmalemma (PM)-enriched proteomes have been reported, and none of them involved an industrially relevant filamentous fungus. The main focus of study has been on *S. cerevisiae* [26–28] and on several pathogenic fungi [29–31], as their PM represents a cellular component of substantial interest from a diagnostic and therapeutic point of view [29]. Thus, since the PM proteome of *A. niger* has not been a subject of study yet, it would be a first step towards a better understanding of its dynamics and topology.

Recently, our research group successfully used a shotgun proteomics approach to study protein secretion mechanisms in *A. niger*, allowing the characterisation of the secretory subproteome of the fungus and its changes in different conditions by using label-free LC–MS/MS [32, 33]. This is a powerful tool to analyse enriched organelle cell fractions, both in qualitative and quantitative terms [34], and thus permits the identification and quantification of the most relevant components of the *A. niger* cell membranes. In this work, the membrane-associated proteome of *A. niger* was studied for the identification of new glucose transporters, using newly developed experimental and complementary computational approaches.

## Results and discussion

### In silico transportome analysis and construction of a hidden Markov model specific for glucose transporters

As an integral part of the membrane, transporters must contain at least one protein domain that is thermodynamically stable in the hydrophobic environment of the phospholipid tails. In eukaryotes, these are typically α-helical structures. Transmembrane proteins can be predicted by applying a transmembrane hidden Markov model (tmHMM) that incorporates the hydrophobicity, charge bias, helix lengths and grammatical constraints of known transmembrane proteins into one model [35]. In addition, a comprehensive list of *A. niger* sugar transporters can be made by applying hidden Markov models for both the major facilitator superfamily (HMM_MFS_), and sugar porters (HMM_SP_). As the largest subfamily of the MFS, the sugar porter (SP) family currently has the most identified members [36], and as such provides a good basis for constructing a specific profile HMM from multiple alignments of extensively characterised members.

**Fig. 1:**
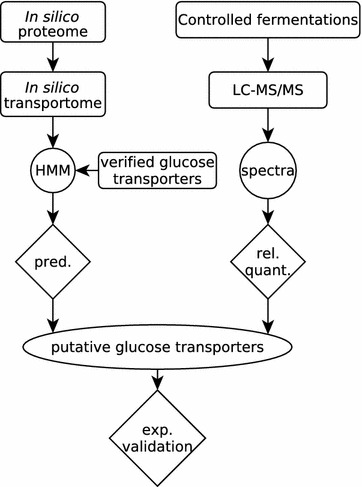
Bioinformatic and proteomic profiling of the *A. niger* transportome. The flow chart outlines the complementary approach taken to identify putative glucose transporters. Experimental validation of the transporters identified was carried out in *S. cerevisiae*

**Fig. 2:**
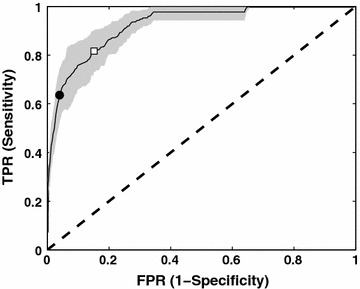
Receiver operating characteristic (ROC) curve for the HMM_gluT_ transporter model. Plotted is the mean of 30 runs of a 10 × 3-fold cross-validation. The confidence interval is shown in *grey*. Calculated inclusion thresholds, *d*_min_ (*white square*) and MCC_max_ (*black circle*), are indicated

The experimental steps and complementary bioinformatics pipeline to identify and validate *A. niger* glucose transporters are depicted in Fig. 1. Initial in silico analysis of the theoretical *A. niger* proteome with the precomputed HMM_MFS_ and HMM_SP_, obtained from the Pfam database [37], showed that more than 250 of the proteins predicted in *A. niger* ATCC1015 have a conserved domain architecture related to sugar transport (Table 1). Very similar results were obtained for the *A. niger* CBS 513.88 strain (not shown). This high abundance of sugar transporters makes *A. niger* a versatile host for the bioconversion of lignocellulosic biomass to products of interest, especially also in comparison to *S. cerevisiae*, which cannot metabolise as many sugars and consequently has fewer sugar transporters [11]. Although the HMM_SP_ captures the conserved domain architecture of potential sugar porters, it cannot discriminate between different sugar substrates, and was thus considered to be too broad for the purpose of this study. Therefore, a list of 42 biochemically characterised glucose transporters from 10 different organisms was obtained from the UniProt database [38], and a profile HMM specific for glucose transporters (HMM_gluT_) was built from these sequences (see alignment in Additional file 1). The functions of proteins are often more conserved in their tertiary structure than in their primary amino acid sequence, as residues that are not crucial for the function of the protein will be subject to evolutionary changes over time. The typical structure of known glucose transporters comprises 12 transmembrane helix domains divided into 2 groups of 6 helixes [39, 40], and the HMM_gluT_ was computed from a structure-based multiple sequence alignment incorporating transmembrane helix predictions, rather than using an alignment algorithm based on the primary amino acid sequence alone. Appropriate threshold values, above which a hit with the HMM_gluT_ has to score in order to be considered a true positive, can be calculated by evaluating the properties of the HMM_gluT_. Receiver operating characteristic (ROC) curves are instrumental in assigning the best threshold values, since they display the trade-off between sensitivity and specificity. In order to obtain ROC curves, the HMM_gluT_ was first validated using a 10 × 3-fold cross-validation approach (Fig. 2). Threshold values with the best trade-off between the true and false-positive rates of the prediction were determined from the resulting ROC curve. In this study, two thresholds were used. The first is *d*_min_, which is the point on the curve that has the minimal distance to [0 1] in the 2-dimensional *x*–*y* plane. Another way to determine the best trade-off point is by calculating the point in which the Matthews correlation coefficient is maximal (MCC_max_), as the prediction is regarded as better the closer the MCC value is to 1, whereas a value of 0 is regarded as no better than a random guess. To compare the performance of this approach to a more traditional approach, in which Blast is used to identify homologous proteins, each of the 42 verified glucose transporters was used as query in a separate Blast search against the same dataset used to evaluate the HMM_gluT_. The resulting ROC curves for each of the methods is depicted in Additional file 2 and shows that, for the datasets used, an approach using HMMs outperforms Blast in discriminating glucose transporters from other sugar transporters.

**Table 1:** MFS domain proteins in *A. niger* and *S. cerevisiae*

	*N*_tot_	*N*_tmHMM_	*N*_MFS_	*N*_SP_	*N*_gluT_	*N*_gluT_ (*d*_min_)	*N*_gluT_ (MCC_max_)
*A. niger* ATCC1015	11,910	2561	469	256	252	19	5
*S. cerevisiae* CEN.PK	5439	1022	73	43	45	15	12

*N*_*tot*_ total number of predicted proteins, *N*_*tmHMM*_ number of proteins containing at least one transmembrane helix domain, *N*_*MFS*_ number of proteins containing a MFS domain, *N*_*SP*_ number of proteins containing an SP domain, *N*_*gluT*_ number of proteins above inclusion threshold of HMM_gluT_ (as given by the HMMER3.0 tool), *N*_*gluT*_ (*d*_*min*_) number of proteins above the inferred threshold score at *d*_min_, *N*_*gluT*_ (*MCC*_*max*_) number of proteins above the inferred threshold score at MCC_max_

By using the previously calculated thresholds as lower limits for the HMM_gluT_ score, the list of putative glucose transporters was effectively narrowed down to 19 sugar porters when the *d*_min_ threshold was applied, and to only 5 sugar porters, including MstA, when the MCC_max_ threshold was applied (Table 1). Note that MstA was truly ‘discovered’ by the HMM_gluT_, since it was not a priori included in the training set. The average sensitivity, specificity, accuracy and inferred HMM_gluT_ scoring values at the two threshold points, *d*_min_ and MCC_max_, are noted in Table 2. As expected, the specificity at the MCC_max_ threshold is very high, but it comes at the cost of having a lower sensitivity. The *d*_min_ threshold on the other hand is less strict, thus allowing for a higher false-positive rate in order to not neglect any true positives.

**Table 2:** Average sensitivity, specificity, accuracy and inferred HMM_gluT_ threshold values for the HMM_gluT_ at *d*_min_ and MCC_max_

	Sensitivity (%)	Specificity (%)	Accuracy (%)	HMM_gluT_ score
*d*_min_	81.67	84.79	84.61	379.41
MCC_max_	63.57	96.00	94.12	531.03

### The *A. niger* plasmalemma proteome in response to different glucose concentrations at low pH

A way to verify both the HMM_gluT_ and the predicted glucose transporter candidates is by using an experimental setup, in which the abundance of transmembrane proteins in relevant conditions can be compared with their HMM_gluT_ scores. In this complementary work, the focus was put on the study of the *A. niger* transportome in the presence of different concentrations of glucose at a pH which is both physiologically and biotechnologically relevant for this fungus. Mycelium of *A. niger* N400 was pre-grown for 18 h in minimal medium with 100 mM sorbitol as sole carbon source and subsequently transferred in equal amounts to controlled fermenters containing minimal medium with the following carbon source compositions: 100 mM sorbitol (reference condition), 100 mM sorbitol plus 1 mM glucose (low-glucose condition) and 100 mM sorbitol plus 60 mM glucose (high-glucose condition). The initial pH of these cultures was set at pH 4.0, which corresponded with the final pH measured at the pre-growth stage. Immediately after inoculation the pH of the fermenter cultures started to drop, reaching pH 3.5, in all cases, after 50–60 min. For the remainder of the experiment this was kept constant at pH 3.5. High-resolution analysis of the sugar content in the culture medium sampled 2 h after inoculation showed that there was sorbitol consumption in the reference condition; sorbitol and glucose consumption in the low-glucose condition; and glucose but no sorbitol consumption in the high-glucose condition. This result confirmed, as reported [41], that the organism strongly favours consumption of good carbon sources like glucose, over poorer carbon sources, such as sorbitol, even when the latter is also present at a high concentration (100 mM). The 2-h time-point was selected for membrane-associated protein analysis in all three conditions.

Isolation of fractions enriched for cell membranes was performed using a protocol similar to the one developed by Oliveira and co-workers [32]. This protocol involves the previously described workflow: cell disruption, crude organelle separation, and subsequent enrichment [42]. After several differential centrifugation steps, a pellet containing crude low-density organelles (P3) was obtained and subjected to density gradient centrifugation, yielding a set of five fractions (P3A–P3E). The PM marker vanadate-sensitive H^+^ ATPase and the mitochondrial membrane cytochrome *c* oxidase activities were then measured in the initial cell-free extract, the P3 pellet and the P3A to P3E fractions derived from it. Compared to the cell-free extract, the P3 pellet was shown to be 2.4–3.2 times enriched in plasma membranes. No further enhanced PM enrichment was found in the P3A to P3E fractions; however, cytochrome *c* oxidase activity was higher in the P3A to P3E fractions when compared to P3. Since mitochondrial membranes were not the main focus of this research, the P3 pellets, considered to be more optimal for the analysis of plasmalemma proteins, were further processed and subjected to shotgun proteomics analysis (detailed information regarding subcellular fractionation, marker enzyme assays and sample preparation for LC–MS/MS can be found in the “Methods”).

The LC–MS/MS spectra obtained were processed as described in the “Methods”. In total, 833 proteins were identified, of which 432 were present in all three conditions, 72 proteins were found only in the reference condition, 34 only in the low-glucose condition, and 106 proteins were found exclusively in the high-glucose condition. Of the proteins identified, almost 30 % had one or more predicted tmHMM domains, indicative of integral membrane proteins (Additional file 3). The relative abundances of the proteins containing at least one tmHMM domain, which add up to 15.28, 17.50 and 15.45 % of the total protein isolated in the reference, low- and high-glucose conditions, respectively, can be found in Additional file 4. Proteins associated with the mitochondrial membrane were most abundant, followed by the proteins associated with the plasma membrane. However, the plasma membrane-associated fraction consists of a higher number of different proteins than the mitochondrial membrane-associated fraction, with 86 and 35 identified proteins, respectively. Proteins associated with the membrane-bound endoplasmic reticulum (ER) constitute the third most abundant group of the list, with a total of 48 different proteins identified. Finally, proteins linked to other membrane-associated organelles, comprising the endomembrane system and membrane-bound organelles, such as the Golgi apparatus, vacuoles and lysosomes, account for <1 % of the total protein isolated (Additional file 5). In all three conditions, approximately one-third of the proteins that have one or more tmHMM domains are annotated as proteins with a transporter function. This fraction, denoted as the *A. niger* transportome, accounted for 3.9, 4.4 and 4.1 % of the total protein isolated in the reference, low- and high-glucose conditions, respectively. Mitochondrial carrier proteins are prevalent in all three conditions, followed by ATPases. Proteins with the MFS architecture, which are the main interest in this study, comprise the third most abundant group of the isolated *A. niger* transportome. Proteins (putatively) involved in the secretory pathway and amino acid transport were found in relative abundances of <0.5 % of the total protein isolated. Other transporters, comprising (putative) oligopeptide transporters, formate/nitrite transporters, ammonium transporters, iron permeases, Na^+^/solute symporters, ABC transporters, inorganic phosphate transporters, nucleotide-sugar transporters, major intrinsic proteins and the translocation protein Sec62, accounted for ≤0.01 % of the total protein isolated, respectively.

### Performance of the HMM_gluT_ for the identification of candidate glucose transporters

The *A. niger* transportome was further analysed for the presence of glucose transporters. Table 3 summarises the results of the bioinformatics analysis, which was performed in the same way as previously for the entire *A. niger* in silico proteome, i.e. the data were first queried with the hidden Markov models specific for proteins containing a MFS or SP domain. As already observed for the *A. niger* in silico proteome, a search with the HMM_SP_ lowered the number of proteins that scored above the inclusion threshold in comparison to the search performed with the less specific HMM_MFS_. Querying the transportome with the newly developed HMM_gluT_, using the previously calculated thresholds, lowered the number of putative, albeit highly probable, glucose transporters to 4, 3, and 2 promising hits in the reference, low- and high-glucose conditions, respectively.

**Table 3:** Number of MFS porter proteins found in the three growth conditions

	*N*_MFS_	*N*_SP_	*N*_gluT_	*N*_gluT_ (*d*_min_)	*N*_gluT_ (MCC_max_)
Sorbitol	14	13	13	4	4
Sorbitol + 1 mM glucose	9	6	7	3	3
Sorbitol + 60 mM glucose	15	9	10	2	2

*N*_*MFS*_ number of proteins containing a MFS domain, *N*_*SP*_ number of proteins containing a SP domain, *N*_*gluT*_ number of proteins above the default inclusion threshold of HMM_gluT_ (as given by the HMMER3.0 tool), *N*_*gluT*_ (*d*_*min*_) number of proteins above inferred threshold score at *d*_min_, *N*_*gluT*_ (*MCC*_*max*_) number of proteins above inferred threshold score at MCC_max_

**Fig. 3:**
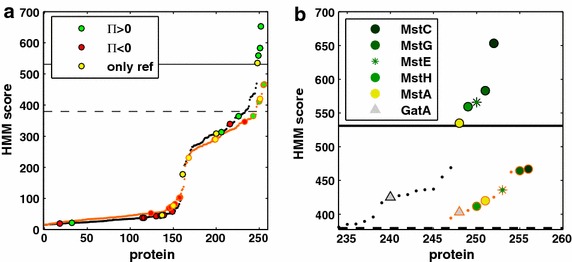
HMM_SP_ and HMM_gluT_ scores of MFS porter proteins. **a** HMM scores for the theoretical ATCC1015 proteome, with HMM_gluT_ = *black dotted line* and HMM_SP_ = *orange dotted line*. *Horizontal lines* indicate the HMM_gluT_ thresholds *d*_min_ (*dashed*) and MCC_max_ (*solid*). MFS porter proteins found in the three conditions applied are indicated with *filled circles* and *colour*-coded according to their relative abundance patterns. **b** Close-up of the proteins that scored above the *d*_min_ (*dashed line*) and MCC_max_ *(solid line*) thresholds. The galacturonic acid transporter GatA (indicated with a *grey* *triangle* and not found in the conditions applied) is added as a reference. With HMM_gluT_, the Mst transporters are well separated from GatA, whereas the more general HMM_SP_ does not discriminate the putative glucose transporters from the galacturonic acid transporter

The relative abundances of the identified MFS proteins that scored above the default inclusion threshold of HMM_gluT_, HMM_SP_ and HMM_MFS_, ordered by their HMM_gluT_ scores, can be found in Table 4. High HMM scores corresponded to MFS porters that have been related to glucose uptake in previous studies, or novel MFS porters that, in the present study, showed abundance patterns that point towards a possible role as glucose transporter. In Fig. 3a, the proteins found in the three conditions are highlighted on the HMM curves for the glucose and general sugar transporter model depicted. The colour coding corresponds to their relative abundances in the three conditions (see “Methods” for details). The HMM curves as such show the scores of all proteins in the theoretical *A. niger* ATCC1015 proteome that hit above the default inclusion threshold of both HMM_gluT_ and HMM_SP_. The majority of proteins that were more abundant in the low-glucose condition relative to both the reference and high-glucose condition cluster closer to the *d*_min_ and MCC_max_ thresholds, whereas the proteins that were more abundant in the high-glucose condition scored overall lower on the HMM_gluT_. This indicates that the model is better at detecting high-affinity glucose transporters, which might be due to the sequences that were selected to build the HMM_gluT_ (Additional file 1). A close-up of the top scoring proteins is depicted in Fig. 3b. The highest score was obtained with protein ID 1121621, which is the putative low-affinity glucose transporter MstC. Studies on its transcriptional regulation during the exponential growth phase in batch fermentation and in chemostat cultures, carried out at different dilution rates, have been performed by Jørgensen et al. [9]. The *mstC* transcripts were only detected during the batch fermentation phase, indicating that *mstC* expression is associated with higher glucose concentrations or with specific growth rates. In the present work, the protein abundance of the MstC protein was found to be similar in all the three conditions studied. This result, together with the previous findings of Jørgensen et al., suggests that the presence of MstC is independent of the glucose concentration. The second best hit, with protein ID 1142882, and the fourth best hit, with protein ID 1143598, are yet-uncharacterised transport proteins, henceforth denoted as MstG and MstH, respectively. Their high HMM_gluT_ scores and overall abundance pattern in the three experimental conditions strongly indicate a possible role for them as glucose transporters. MstG and MstH were found with relatively high abundance levels only in the two non-carbon catabolite-repressing conditions studied, being higher in abundance when low glucose concentrations were present. This type of abundance pattern fits that of high-affinity glucose transporters described in *S. cerevisiae* and *Aspergillus* species [14, 43, 44], that are preferably expressed in the presence of low concentrations of glucose, poor carbon (de-repressing) sources and also in starvation conditions. Another protein with protein ID 1125134, scoring in between MstG and MstH in the HMM_gluT_ was not observed in the three studied conditions. It is denoted as MstE (An03g02190) in the UniProt database, and has been found to be expressed in germinating spores [45]. The fifth highest scoring hit, with protein ID 1143191, is MstA. The *mstA* coding gene is transcriptionally controlled by the carbon catabolite repressor CreA and the environmental pH regulator PacC. Its transcript levels were found to be higher during carbon starvation at pH 6.0 [14]. Van Kuyk et al. also observed low expression levels at pH 4.0 and 8.0, and in the presence of repressing glucose concentrations at pH 6.0 [14]. In the present study, MstA was detected in very low abundance; however, only in the de-repressing reference condition, supporting previous results that suggested a limited role of this transporter when the environmental pH is low. The combined results of the specific protein abundance patterns and the high HMM_gluT_ scores indicate that MstG and MstH could have a role as high/mid-affinity glucose transporters, therefore both transporters were selected for further characterisation.

**Table 4:** Relative abundance and HMM scores of MFS porter proteins detected in the three growth conditions

protID	HMM_gluT_	HMM_SP_	HMM_MFS_	Relative abundance ± sd (%) × 100			Remark
				Sorbitol	Sorbitol + 1 mM glucose	Sorbitol + 60 mM glucose	
1121621	653.1	467	78.5	9.08 ± 1.02	11.89 ± 4.79	9.47 ± 3.50	MstC (Q8J0U9)
1142882	583.1	464.5	74.8	2.24 ± 0.48	4.47 ± 0.20	n.d.	MstG
1143598	559.3	411.7	86.1	6.38 ± 1.83	17.54 ± 2.39	n.d	MstH
1143191	535	420	75.7	8.51e−3 ± 1.14e−3	n.d.	n.d.	MstA (Q8J0V1)
1101809	364.4	364.7	85.6	4.83 ± 0.51	2.01 ± 0.29	n.d.	
1180703	339.2	346.3	97.4	n.d.	n.d.	1.37 ± 0.14	MstD (Q8J0U8)
1188093	312.9	289.3	82.0	1.08 ± 0.39	0.82 ± 0.11	0.82 ± 0.25	
1144791	308.1	289.6	55.9	6.25 ± 1.25	n.d.	n.d.	
1189214	177.3	230.6	73.7	0.29 ± 0.03	n.d.	n.d.	
1128338	57.6	103.6	72.9	3.70 ± 0.32	4.36 ± 0.02	6.46 ± 0.84	
1111630	46.4	101.6	63.2	n.d.	n.d.	1.06 ± 0.10	
1184634	46	76.4	0	0.30 ± 4.12e−5	n.d.	n.d.	
1122202	44.6	52.6	135.9	0.87 ± 0.10	n.d.	0.48 ± 0.29	
1178623	42.9	68.3	149.3	n.d.	n.d.	4.71 ± 1.07	
1164538; 1188786	37.5; 37.4	82.3; 72.1	104.3; 98.9	2.11 ± 0.14	n.d.	2.60 ± 0.21	Same protein group^a^
1089440	21.4	0	92.5	n.d.	1.16 ± 0.12	n.d.	
1118545	19.1	0	0	n.d.	n.d.	3.87 ± 1.13	
1105147	0	0	54.4	8.93 ± 0.03	7.14 ± 2.01	11.47 ± 1.24	
1129336	0	0	31.8	0.34 ± 0.01	0.50 ± 0.10	1.07 ± 0.01	
1124902	0	0	130.7	n.d.	n.d.	1.37 ± 0.44	
1165706	0	0	114.3	n.d.	n.d.	0.05 ± 0.01	
1188840	0	0	99.1	n.d.	n.d.	3.00 ± 1.30	
1146101	0	0	65.3	n.d.	n.d.	1.21 ± 0.91	

UniProt accession numbers in brackets

*n.d.* not detected in one or both of the biological replicates

^a^These proteins could not be distinguished by the proteomics analysis and were thus grouped together to one protein group

### Transcriptional analysis of *mstG* and *mstH* in the presence of different carbon sources

**Fig. 4:**
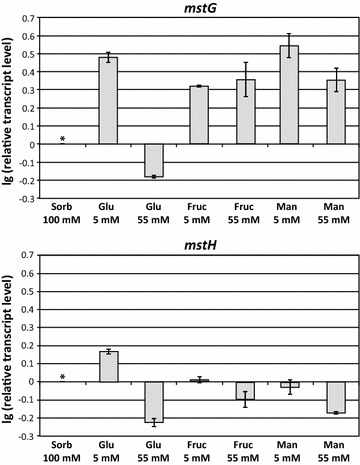
Transcriptional analysis of *mstG* and *mstH*. Mycelium of the *A. niger* strain N400 was precultured on minimal medium with sorbitol 100 mM and thereafter transferred to minimal medium with the following carbon sources: sorbitol 100 mM, glucose 5 mM, glucose 55 mM, fructose 5 mM, fructose 55 mM, mannose 5 mM and mannose 55 mM. Samples from the seven culture conditions were taken 2 h after mycelium transfer, and expression analyses of *mstG* and *mstH* were performed by qPCR using a histone-like gene (gene ID 207921) transcript for normalisation. Results are given as relative transcript rations in logarithmic scale (lg). The *values* provided in the figures are means of two biological replicates. Transcript levels always refer to the reference sample (sorbitol 100 mM), indicated with an *asterisk*

In order to obtain additional insights in the regulation and possible biological role of MstG and MstH, transcriptional levels were analysed in mycelium samples from cultures containing: minimal medium with 100 mM sorbitol (used as a reference condition) and minimal medium plus glucose, fructose or mannose at high (55 mM) or low (5 mM) concentration. Samples taken 2 h after mycelium transfer were processed and RT-qPCR analysis was performed. Both genes were found to be expressed in all the studied conditions, but their expression levels were clearly influenced by the different culture conditions (Fig. 4). The relative transcript levels of both genes in the reference, low-glucose and high-glucose conditions showed a pattern comparable to what was observed at proteomic level; *mstG* and *mstH* were upregulated in the presence of a low glucose concentration and downregulated when the glucose concentration was high. However, *mstG* and *mstH* expression patterns showed to be divergent in the presence of high and low concentrations of fructose and mannose. With these carbon sources *mstG* expression levels were similar to those observed with a low concentration of glucose. In the case of *mstH*, as observed in the presence of glucose 55 mM, high concentrations of fructose (55 mM) and mannose (55 mM) also led to a decrease of its transcript levels when compared to the reference condition. While these results do not provide conclusive evidence that MstG is indeed a high-affinity glucose transporter they are not in disagreement with its possible role either. In the case of *mstH*, only the glucose 5 mM condition enhanced its expression, suggesting that MstH could have a specific role in *A. niger* when low concentrations of the monosaccharide are available.

### Functional validation of the *A. niger* sugar transporters MstG and MstH

**Fig. 5:**
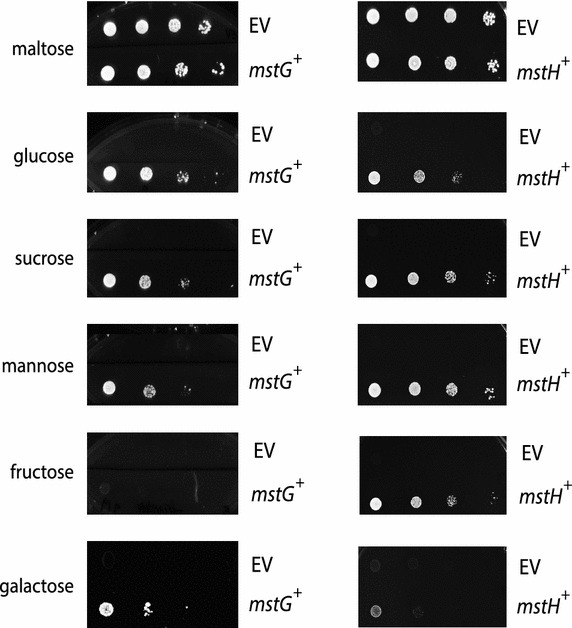
MstG and MstH functional analysis. Growth of strain EBY.VW4000 containing the *mstG* gene (*mstG* ^+^), *mstH* gene (*mstH* ^+^), or harbouring the empty expression vector p426HXT7-6His (EV) in minimal medium agar plates containing the following sugars at a final concentration of 1 % (w/v): maltose, glucose, sucrose, mannose, fructose and galactose. Agar plates were incubated at 30 °C for 96 h. All transformants expressing each gene showed the same growth pattern; therefore, the figure depicts only one transformant per transporter as representative

As discussed above, the high HMM_gluT_ scores, the protein abundance patterns and transcriptional analyses of the MFS porters MstG and MstH point towards a role in glucose uptake. In order to test their functionality, the engineered *S. cerevisiae* strain EBY.VW.4000 [12], a glucose transporter null mutant unable to grow on glucose, mannose, galactose or fructose as carbon source, was chosen as host for functional complementation analysis. The yeast strain was transformed with the 2μ expression plasmid p426HXT7-6His-mstG or p426HXT7-6His-mstH, containing the respective cDNA under control of the constitutive promoter HXT7_p_ and the terminator CYC1_t_. Single colony transformants were isolated from minimal medium agar plates containing 2 % maltose and the ability of both genes to restore growth of the EBY.VW.4000 transformant strain in the presence of different monosaccharides was studied. Tenfold serial dilutions of exponentially growing cells from at least two different transformants expressing each gene were spotted on different minimal medium plates supplemented with 1 % (w/v) of the following carbon sources: glucose, galactose, fructose, mannose, sucrose and maltose (Fig. 5). The *mstG* transformants were able to grow on glucose, galactose, mannose and sucrose as single carbon sources, whereas *mstH* transformants grew on glucose, sucrose, mannose, galactose and, in contrast to *mstG* strains also on fructose. MstH transformants also showed better growth on mannose and sucrose, but poorer growth on galactose. Regarding the sucrose transport by both transporters, since the EBY.VW4000 strain encodes an extracellular invertase [18], it is unknown if the transported substrate was sucrose or glucose in the case of MstG, and sucrose, glucose or fructose in the case of MstH. These results indicated that MstG and MstH are functional sugar transporters with the ability to transport a variety of substrates.

In order to have better insights about the affinity for glucose of both transporters, the growth rates of the transformants expressing MstG and MstH were studied in the presence of different glucose concentrations. Additional file 6 shows growth curves and glucose consumption figures of the transformant strains grown on minimal medium with glucose 2.5, 10 and 50 mM. Both transporters showed to be functional in all glucose concentrations. However, when comparing individual growth curves and glucose uptake, some differences could be observed. The growth rate of the MstG transformant during the exponential growth phase was comparable in the three different conditions, while in case of the MstH transformant, at the highest glucose concentration the growth rate was reduced. Accordingly, the glucose consumption rate of the MstG transformant in the 50 mM condition was much faster, being able to deplete the monosaccharide after around 40 h of growth, whereas in the same time-span the MstH transformant was only able to consume a 60 % of the total amount suggesting that MstG and MstH are glucose transporters with different affinities for the sugar.

**Fig. 6:**
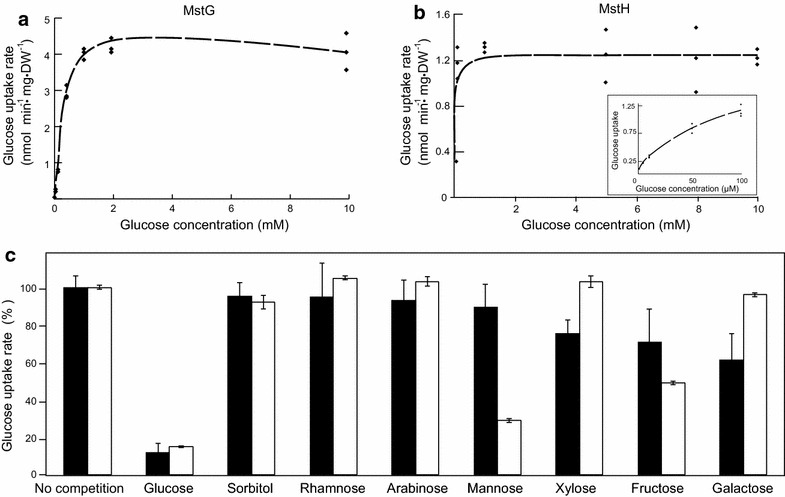
Uptake of ^14^C-labelled glucose by the yeast hexose transporter null mutant EBY.VW4000 expressing mstG or mstH as a function of glucose concentration. **a**, **b** *Diamonds* represent measured uptake rates of ^14^C labelled glucose (*n* = 3) as a result of expression of *mstG* (**a**) or *mstH* (**b**). *Black lines* represent least-squares-fitted Michaelis–Menten model (*mstG*; *K*_m_ = 0.5 mM; *V*_max_ = 5.8 nmol min^−1^ mg DW^−1^; *mstH*: *K*_m_ = 0.06 mM, and a *V*_max_ = 1.3 nmol min^−1^ mg DW^−1^). *Insert* results for *mstH* using micro-molar substrate concentrations. **c** Uptake rate of 1 mM ^14^C-labelled glucose by EBY.VW4000 expressing *mstG* (*filled boxes*) or *mstH* (*open boxes*) in the presence of a tenfold excess of competing sugars

To determine MstG and MstH transport characteristics, (^14^C) glucose transport assays were performed with the transformant strains as described [46]. Initial glucose uptake rates at various substrate concentrations were fitted to the Michaelis–Menten model with non-competitive substrate inhibition and used to estimate the appropriate kinetic parameters as described [47]. MstG was confirmed to transport glucose with apparent *K*_m_ values of 0.5 ± 0.04 mM, and *V*_max_ of 5.8 ± 0.04 nmol min^−1^ mg DW^−1^ (Fig. 6). Its affinity for glucose was lower than the one reported for *A. niger* MstA with a *K*_m_ value of 0.025 ± 0.01 mM. However, MstG can still be classified as a high-affinity glucose transporter, since its *K*_m_ value is lower than the one reported for the *S. cerevisiae* high-affinity hexose transporter HXT6 (*K*_m_ value 1.4 ± 0.1 mM) [47]. Initially MstH kinetics were determined using the same range of substrate concentrations as for MstG. The results obtained suggested that MstH had a much higher affinity for glucose than MstG. Therefore, the assay was optimised and repeated using a micro-molar range of glucose concentrations. MstH was confirmed to transport glucose with apparent *K*_m_ values of 0.06 ± 0.005 mM and a *V*_max_ of 1.3 ± 0.2 nmol min^−1^ mg DW^−1^ (Fig. 6), similar to what has been reported for *A. niger* MstA.

Since MstG and MstH were able to restore growth of the hexose non-transporting EBY.VW4000 on other monosaccharides as well, their substrate specificity was evaluated by determining glucose relative transport levels in the presence of a tenfold excess of various competing sugars (Fig. 6). Both transporters clearly showed higher specificities for glucose. However, besides glucose, galactose, fructose and xylose were able to inhibit MstG glucose transport by 39, 29 and 25 %, respectively; whereas, in the MstH transformant mannose and fructose reduced the uptake of labelled glucose by 70 and 50 %, respectively. *A. niger* MstA also showed a preference for glucose, while being able to transport other monosaccharides as well [14]. Similarly, substrate specificity of *Aspergillus nidulans* hexose transporters HxtB and HxtC have been assayed [20], and a reduction of glucose uptake of 70–80 % was observed for galactose, fructose and mannose. In accordance to the functioning of many glucose transporters from other filamentous fungi, the function of MstG and MstH was found to be energy dependent via proton symport. This was confirmed by measurements of glucose initial uptake rate in the presence of CCCP that resulted in a 60 ± 8 % (*n* = 3) and 64 ± 6 % (*n* = 3) reduction of labelled glucose uptake by MstG and MstH, respectively.

## Conclusions

In this study, two complementary approaches were used to both increase the understanding of the *A. niger* membrane-associated proteome, and to identify novel glucose transporters. In a purely in silico approach, a hidden Markov model (HMM_gluT_) was constructed for the identification of glucose transporters. HMM_gluT_ performed well in the identification and segmentation of functionally validated glucose transporters. In a complementary in vivo approach, defined culture conditions were applied to study the response of the *A. niger* membrane-associated proteome to different glucose concentrations. To the best of our knowledge, this is the first study on the membrane-associated proteome of the industrially relevant fungus *A. niger.* The study provided a better understanding of the membrane composition and topology, especially with respect to proteins with a putative transporter function; the *A. niger* transportome. Analysis of the *A. niger* in vivo transportome with HMM_gluT_ was shown to be effective; by combining the abundance patterns of the proteins identified in the experimental conditions with their respective HMM_gluT_ scores, two new putative glucose transporters, MstG and MstH, were identified. They were functionally validated in an engineered yeast strain with a monosaccharide transporter null-background and confirmed to be high-affinity transporters for glucose.

## Methods

### In silico transportome analysis and construction of a hidden Markov model specific for glucose transporters

The *A. niger* ATCC1015 and *S. cerevisiae* CEN.PK proteomes plus their annotations, which were used for the in silico analysis, were obtained from the JGI database [48]. For *A. niger* ATCC1015, only the best model proteome was used. Transmembrane helix domains were predicted with a standalone version of the TMHMM tool from the TMHMM website [35]. Hidden Markov models for the major facilitator superfamily (HMM_MFS_) and sugar porter (HMM_SP_) domains were obtained from the Pfam database [37].

The protein sequences used to build the HMM_gluT_ were obtained by entering the search term “sugar transporter” in the uniprot database and downloading the resultant xml and canonical fasta files. The xml file was parsed for proteins that were not experimentally verified to have the biological function assigned to them. The remaining proteins were divided into two separate datasets; the first dataset contained only the proteins with an experimentally verified GO term related to glucose transport (GO:0005355 and GO:0015758); the second dataset contained the remaining proteins (core dataset). The protein sequences of the experimentally verified glucose transporters were aligned by accessing the PRALINE structural alignment tool [49] via the SOAP interface and using the following settings: BLOSUM62, PSI-BLAST pre-profile processing (Homology extended alignment, 3 iterations), structural features: DSSP-defined secondary structure search, PSIPRED and TMHMM, fasta outputfile. The aligned fasta output was converted to the Stockholm format, and the HMM_gluT_ was built from the resultant output.sto file using the “hmmbuild” command from HMMER v3.0 [50]. *A. niger* MstA was excluded from the multiple alignment.

For the threefold cross-validation, the initial dataset, containing the 42 verified glucose transporters, was randomly partitioned into three equally sized subsets. The model was then built from every combination of two subsets, while the validation of the model was carried out on the core dataset plus the third subset of the glucose transporters not used for the model building process. This allowed for the determination of false and true negatives in comparison to false and true positives, and thus gave an estimate of the prediction power of the model. The threefold cross-validation was repeated ten times, each time with different random subsets of the verified glucose transporters. Two thresholds were determined from the average ROC curve resulting from the 10 × 3-fold cross-validation of the HMM_gluT_:

1. Minimal distance to the point [0 1] (*d*_min_):

$$ d = \sqrt {(1 - s_{\text{n}} )^{2} + (1 - s_{\text{p}} )^{2} } . $$

With *s*_n_ = sensitivity (TPR) and s_p_ = specificity (TNR).

Another metric to calculate the optimal trade-off point between true and false predictions is the Matthews correlation coefficient. The Matthews correlation coefficient is essentially a one number representation of the confusion matrix, and is thus suitable to compare the outcome of different model predictions. This coefficient is calculated as follows:

2. Matthews correlation coefficient (MCC):

$$ {\text{MCC}} = \frac{{{\text{TP}} \cdot {\text{TN}} - {\text{FP}} \cdot {\text{FN}}}}{{\sqrt {\left( {{\text{TP}} + {\text{FP}}} \right)({\text{TP}} + {\text{FN}})({\text{TN}} + {\text{FP}})({\text{TN}} + {\text{FN}})} }} $$

Here, a value closer to 1 is clearly a better prediction, and thus the inferred HMM_gluT_ score at the highest MCC value calculated along the ROC curve, MCC_max_, was set as second threshold.

Similarly, the Blast approach was evaluated by using each of the 42 verified glucose transporters as separate query for a local Blastp search against the core dataset plus the other verified glucose transporters, allowing for the determination of true and false-positive rates (see Additional file 2).

### Strains and growth conditions

*Escherichia coli* DH5α (*endA*1, *hsdR*17, *gyrA*96, *thi*-1, *relA*1, *supE*44, *recA*1, Δ *lacU*169 (Φ80 *lacZ*ΔM15)), grown at 37 °C, was used for cloning experiments and plasmid propagation. Luria broth (LB) was used as growth medium (1 % w/v tryptone, 0.5 % w/v yeast extract, 1 % w/v NaCl) with or without 100 μg mL^−1^ ampicillin.

The wild-type strain *A. niger* N400 (CBS 120.49), used for the plasma membrane proteomics analysis, was grown at 30 °C on complete medium plates for spores generation and maintenance [51]. Mycelial biomass of the N400 strain was obtained after 18 h of growth in liquid cultures containing minimal medium [51], including 5 g L^−1^ of yeast extract, with 100 mM sorbitol as carbon source. Equal amounts of water-rinsed mycelium were transferred to 1-L benchtop fermenters (Sartorius) with 750 mL of minimal medium containing 4.50 g NaNO_3_, 1.13 g KH_2_PO_4_, 0.38 g KCl, 0.38 g MgSO_4·_7 H_2_O and 750 µL of Vishniac solution [51, 52]. Three different conditions, varying on the carbon source composition, were studied: sorbitol 100 mM (reference condition), sorbitol 100 mM plus glucose 1 mM (low-glucose condition) and sorbitol 100 mM plus glucose 60 mM (high-glucose condition). Two biological replicates per condition were studied. Fermenters were stirred at 1000 rpm and aerated with filtered air (0.6 L min^−1^), keeping oxygen levels over 60 %. The initial pH, set at 4.0, was allowed to drop until pH 3.5 and kept constant afterwards by sodium hydroxide addition.

Mycelium samples for RT-qPCR analysis were obtained from a mycelium transfer experiment similar to the one described above, using minimal medium with the following carbon source compositions: 100 mM sorbitol (reference condition), glucose 5 mM, glucose 55 mM, fructose 5 mM, fructose 55 mM, mannose 5 mM and mannose 55 mM. Two biological replicates per condition were studied as well. Cultures were performed in Erlenmeyer flasks, stirred at 200 rpm and the initial pH of the medium in all conditions was set at 4.0. Two hours after inoculation mycelium samples were taken and quickly washed, dried with a single-use towel, snap-frozen with liquid nitrogen and stored at −80 °C until further processing.

The *S. cerevisiae* strain EBY.VW4000 (CEN.PK2-1C *hxt13Δ*::*loxP*; *hxt15::ΔloxP*; *hxt16Δ*::*loxP*; *hxt14Δ*::*loxP*; *hxt12Δ*::*loxP*; *hxt9Δ*::*loxP*; *hxt11Δ*::*loxP*; *hxt10Δ*::*loxP*; *hxt8Δ*::*lox*P; *hxt514Δ*::*loxP*; *hxt2Δ*::*loxP*; *hxt367Δ*::*loxP*; *gal2Δ*; *stl1Δ*::*loxP*; *agt1*::*loxP*; *ydl247wΔ*::*loxP*; *yjr160cΔ*::*loxP*), used for the functional complementation experiments and the characterisation of glucose transporters [12], was grown at 30 °C and maintained in solid complete medium containing 10 g L^−1^ of yeast extract, 20 g L^−1^ of peptone and 20 g L^−1^ of maltose. The EBY.VW4000-derived strains obtained in the present study were grown in liquid minimal medium containing 6.7 g L^−1^ of yeast nitrogen base with ammonium sulphate (Difco), 20 g L^−1^ of maltose, supplemented with leucine (30 mg L^−1^), tryptophan (20 mg L^−1^) and histidine (20 mg L^−1^).

### *A. niger* membrane-associated protein purification and quality control analysis

*Aspergillus niger* mycelium samples (2–3 g, press-dried), washed with a 20 mM HEPES buffer pH 7.6 containing 150 mM NaCl, and resuspended in the same solution containing 1 % (v/v) protease inhibitor cocktail for yeast and fungi (Sigma), were mechanically disrupted using a French press (8000 psi). Cell-free extracts were centrifuged for 5 min at low speed (500*g*), in order to remove unbroken cells and pellet debris. The supernatants were then centrifuged during 20 min at medium speed (5000*g*), to pellet and remove remaining heavy organelles. The remaining supernatants were centrifuged for 120 min at high speed (~85,000*g*), to pellet light organelles (P3).

P3 pellets were resuspended using a Dounce homogenizer in 1 mL of a 20 mM HEPES buffer pH 7.6 containing 250 mM sucrose. P3 suspensions were overlayed in a discontinuous sucrose density gradient, prepared by layering successive decreasing sucrose densities solutions (6 × 1 mL), with concentrations ranging from 1.20 to 0.70 M, upon one another. Sucrose density gradients were centrifuged (~100,000*g*, 60 min) to isolate different membrane-associated fractions from P3 pellet. Five fractions were obtained (P3A, P3B, P3C, P3D and P3E).

### Sample preparation for LC–MS/MS

The protein content of enriched plasma membrane-associated fractions was determined using the BCA protein assay. Membrane proteins were solubilised by mixing volumes of each fraction containing 25 μg of protein with equal volumes of a 2× solution of 20 mM HEPES pH 7.6 containing 1 M 6-aminocaproic acid and 10 g L^−1^ of *n*-dodecyl-beta-d-maltoside. Cell membrane–detergent mixes were incubated in a thermoblock (ThermoMixer) for 1 h at 20 °C and vigorous stirring (1000 rpm). Afterwards, samples were sonicated in a water bath for 15 min, and finally they were centrifuged at 22,000*g* for 30 min. Supernatants containing solubilised proteins were concentrated using MMicrocon YM-10 columns (cutoff, 10 kDa; Millipore, Eschborn, Germany) and loaded into a 12 % SDS–polyacrylamide gel, which was run until the loaded samples entered into the gel. The gel was stained according to the manufacturer’s instructions using Page Blue staining (Fermentas) and rinsed with ultrapure water. Each sample-gel lane was cut into one slice (approx. 1 cm^2^), carefully sliced into smaller pieces of about 1 mm^3^ and transferred into microcentrifuge tubes. Samples were destained and equilibrated through three washing steps using the following solutions: 50 mM ammonium bicarbonate (ABC) (incubated 5 min), ABC/acetonitrile (1:1, v/v) (incubated 5 min) and neat acetonitrile (incubated 5 min). These washing steps were successively repeated two times. The gel samples were then swelled in 10 mM dithiothreitol (DTT) for 20 min at 56 °C to reduce protein disulfide bonds. Subsequently, the DTT solutions were removed and samples were alkylated with 50 mM 2-chloroacetamide in ABC, for 20 min, at room temperature, in the dark. The 2-chloroacetamide solutions were removed, and samples were again washed twice with: neat acetonitrile (incubated 5 min), ABC (incubated 5 min) and neat acetonitrile (incubated 5 min). Approximately 150 μL of digestion buffer, containing sequencing grade modified trypsin (12.5 ng μL^−1^) (Promega, Madison, WI, USA) in ABC, was added to each sample, making sure that all gel pieces were kept wet during digestion (adding, if necessary, additional ABC solution). Protein samples were digested overnight at 37 °C. Peptide digestion products were extracted by adding 50 μL of 2 % trifluoroacetic acid (TFA), followed by an incubation step in a thermoblock (ThermoMixer) for 20 min, at room temperature and vigorous stirring (1400 rpm). Gel pieces were then subjected to 20 s sonication in a water bath, centrifuged and supernatants were transferred to new tubes. The peptide extraction step was then repeated once by washing the gel pieces with buffer B (80 % acetonitrile, 0.1 % formic acid) followed by the mentioned incubation and sonication steps. Supernatants from both extractions were pooled and samples were placed in a vacuum centrifuge for acetonitrile evaporation until 20–40 μL were left. Finally, samples were acidified by addition of TFA (1:1, v/v) and peptide clean-up procedure, prior to LC–MS/MS analysis, was performed using the “STop And Go Extraction” procedure as described before [53].

### Mass spectrometric measurements

LC–MS/MS analysis was performed at Radboud Proteomics Centre as described previously [54]. Measurements were performed by nanoflow reversed-phase C18 liquid chromatography (EASY nLC, Thermo Scientific) coupled online to a 7-Tesla linear ion trap Fourier-Transform ion cyclotron resonance mass spectrometer (LTQ FT Ultra, Thermo Scientific).

### Proteomics data analysis

The LC–MS/MS spectra obtained from the proteomics experiment were identified and quantified using the maxQuant software [55]. The peptides were mapped against the annotated *A. niger* ATCC1015 in silico proteome obtained from the JGI database (http://genome.jgi-psf.org/Aspni7/Aspni7.home.html) using the default settings of the maxQuant version 1.3.0.5 [variable modifications: oxidation (M) and acetylation (protein N-term); enzyme used: trypsin/P; fixed modifications carbamidomethyl (cys)], except for the variables affecting the label-free quantification. For this, the multiplicity was set to 1, and the parameters for label-free quantification as well as the iBAQ and peak property calculations were selected. Only proteins with two or more unique peptide hits were considered for further analysis. Protein localisation was determined using the softberry protComp prediction server (http://linux1.softberry.com). The relative abundances of the identified proteins are represented as follows:

$$ \varPi = \frac{{1 + A_{\text{L}} }}{{1 + A_{\text{S}} }} - \frac{{1 + A_{\text{H}} }}{{1 + A_{\text{S}} }} , $$

where *A*_L_ is the relative abundance of the protein in the low-glucose condition; *A*_H_, relative abundance of the protein in the high-glucose condition; *A*_S_, relative abundance of the protein in the reference (sorbitol) condition.

### Transcriptional analysis of *mstG* and *mstH* genes

Mycelium samples were disrupted with glass beads in a Fastprep-24 instrument, and RNA extraction was performed by a Maxwell 16 instrument using the Maxwell 16 LEV simplyRNA kit (Promega). Reverse transcription and qPCR analysis were performed following the protocols and instruments described in Mach-Aigner et al. [56]. The previously described histone-like gene “*hist*” transcript (gene ID 207921) was used as reference for normalisation of the expression data [56]. The following sequences belong to the primers used for qPCR analysis in this study: hist-FW, ACAATGACTGGCCGTGGAAAGG; hist-RV, ATACGCTTGACACCACCACGAC; mstG-FW, CGGTGGTGGTATGGCTTTCT; mstG-RV, GTTCTCAGGCACACCGTACA; mstH-FW, GCCATCATGATCGGTCTGTTTGTC, mstH-RV, ACTGATGGTTCCGGTGTCATATCC.

### Construction of *S. cerevisiae* EBY.WV4000 transformants expressing *A. niger mstG* and *mstH* genes and glucose uptake assays

The coding sequence of the genes *mstG* and *mstH*, digested with *Spe*I and *Xho*I were cloned on the *S. cerevisiae* expression vector p426HXT7-6His, previously linearised with *Spe*I and *Xho*I under the control of the constitutive promoter HXT7_p_ and the terminator CYC1_t_. Transformation of *S. cerevisiae* EBY.WV4000 with p426HXT7-6His (empty vector), p426HXT7-6His_mstG and p426HXT7-6His_mstH was performed as described [57].

Uptake assays were performed as described with minor adjustments [46]. 5 mL of Synthetic Complete medium without uracil (SC-ura; 0.67 % YNB Difco + dropout supplement mix without uracil Sigma-Aldrich) with 2 % maltose as a carbon source was inoculated from plate with EBY.VW4000 with pRS426H7 (control) and EBY.VW4000 with pRS426H7_mstG or pRS426H7_mstH and incubated overnight (30 °C, 225 rpm) as pre-inoculum. Pre-inoculum was transferred to 500 mL SC-ura with 2 % maltose and incubated for 24 h. Cells were harvested by centrifugation (4000*g*, 10 min) and washed with 50 mL ice-cold MQ. Cells were then resuspended in 2 mL ice-cold SC-ura without carbon source, divided in 40-μL aliquots and kept on ice.

Aliquots were incubated for 5 min at 30 °C before uptake assay was started. To start the reaction, 10 μL of a five times concentrated labelled glucose solution (d-[U-14C]-glucose, Campro Scientific) was added. After exactly 10 s the reaction was stopped by the addition of 1 mL of 100 mM LiCl and vacuum filtration (0.45 μm HV filters, 1225 sampling manifold, Millipore), with subsequent washing with 2 × 5 mL of ice-cold 100 mM LiCl. After 5 min of drying in the vacuum manifold, the filters were transferred to scintillation vials with 7.5 mL scintillation liquid (Ultima Gold, Perkin Elmer) and activity was counted (Packard Tricarb 1600TR). All reactions were performed in triplicates. For each reaction, a negative control assay without incubation was performed.

To determine transport kinetics, uptake of labelled glucose with a range of concentrations from 1 μM to 10 mM was measured in SC-ura at pH 5.0. Glucose solutions with an activity of approximately 700–70,000 Bq were used. To determine kinetic parameters *K*_m_ and *V*_max_, the data were fitted to the following Michaelis–Menten model with substrate inhibition using the least-squares method.

$$ V = \frac{{V_{ \hbox{max} } \cdot [S]}}{{K_{\text{m}} + \left[ S \right] + \frac{{[S]^{2} }}{{K_{i} }}}} $$

To determine substrate specificity, the uptake of 1 mM of labelled glucose was measured in the presence of 10 mM of a competing carbon source. d-glucose, d-sorbitol, d-xylose, d-mannose, l-rhamnose, l-arabinose, d-fructose and d-galactose were selected as competing carbon sources.

To determine if the transporter functions via proton symport, activity was measured while uncoupling the proton gradient by carbonyl cyanide m-chlorophenyl hydrazine (CCCP). This inhibitor is dissolved in DMSO and added before temperature equilibration of the cells. The uptake rate in the presence of 250 μM of CCCP and 2 % DMSO was compared with the uptake rate in the presence of only 2 % DMSO.

## Additional files

**Additional file 1.** Structure based multiple sequence alignment of 42 verified glucose transporters.
**Additional file 2.** ROC curves comparing the performance of Blast vs. HMMs to identify glucose transporters.
**Additional file 3.** Number of proteins identified in the 3 different conditions studied in the *A. niger* membrane-associated proteome analysis.
**Additional file 4.** Relative abundances of proteins from the *A. niger* membrane-associated proteome with at least one tmHMM domain.
**Additional file 5.** Relative abundances of identified *A. niger* proteins grouped according to their location within the cell.
**Additional file 6.** Growth of *S. cerevisiae* EBY.VW4000 expressing the *A. niger mstG* and *mstH* genes, at different glucose concentrations.

## Abbreviations

HMM: hidden Markov model
MFS: major facilitator superfamily
SP: sugar porter
Hxt: hexose transporter
PM: plasmalemma
tmHMM: transmembrane hidden Markov model
ROC: receiver operating characteristic
MCC: Matthews correlation coefficient
*d*: distance
ER: endoplasmic reticulum
Sorb: sorbitol
Glu: glucose
Fruc: fructose
Man: mannose
CCCP: carbonyl cyanide m-chlorophenyl hydrazine
GO: gene ontology
LB: Luria broth
ABC: ammonium bicarbonate
TmD: transmembrane domains
protID: protein identity
rel. ab: relative abundance
sd: standard deviation
O.D.: optical density
