# Supplementary material for: Aspergillus niger membrane-associated proteome analysis for the identification of glucose transporters
Source: Biotechnol Biofuels. 2015 Sep 17;8:150. doi: 10.1186/s13068-015-0317-9 (PMC4574540; doi:10.1186/s13068-015-0317-9)
Supplement: Supplementary file 1 — Additional file 1. Structure based multiple sequence alignment of 42 verified glucose transporters. [file 13068_2015_317_MOESM1_ESM.pdf]

Results colour-coded for transmembrane structure

The current colourscheme of the alignment is for **transmembrane structure type**.

The transmembrane structure for each sequence is represented by a colour. If a sequence in the alignment has no colours assigned, this means that no prediction was possible for that sequence (if this was requested).
The colour assignment is:

TM SEGMENT (T) You have selected to perform transmembrane structure prediction using **TMHMM v2.0** (Krogh et. al, 2001).

```
                         .........10 .........20 .........30 .........40 .........50
(PRED) Q8VZ80_Arabidop  ---------- ---------- ---------- ---------- ----------
(PRED) G1UB10_Candida_  ---------- ---------- ---------- ---------- ----------
(PRED) Q5B0S0_Emericel  ---------- ---------- ---------- ---------- -------MGV
(PRED) P13181_Saccharo  ---------- ---------- ---MAVE--- ENNMPVVSQQ PQAGEDVISS
(PRED) P40886_Saccharo  ---------- ---------- ---MTDR--- KTNLPEEPIF EEAEDDGCPS
(PRED) P32465_Saccharo  ---------- ---------- ---------- --MNSTPDLI SPQKSNSSNS
(PRED) P32466_Saccharo  ---------- ---------- ---------- --MNSTPDLI SPQKSSENSN
(PRED) P39003_Saccharo  ---------- ---------- ---------- --MSQDAAIA EQTPVEHLSA
(PRED) P39004_Saccharo  ---------- ---------- ---------- --MSQDAAIA EQTPVEHLSA
(PRED) P32467_Saccharo  ---------- ---------- ---MSEE--- AAYQEDTAVQ NTPADALSPV
(PRED) P38695_Saccharo  --------MS ELENAHQGPL EGSATVS--- TNSNSYNEKS GNSTAPGTAG
(PRED) P54862_Saccharo  ---------- ---------- ---------- ------MSGV NNTSANELST
(PRED) P40885_Saccharo  ---------- ---------- ---------- ------MSGV NNTSANDLST
(PRED) P39924_Saccharo  ---------- ---------- ---------- ---------- MSSAQSSIDS
(PRED) P53631_Saccharo  ---------- ---------- ---------- ---------- MQSSTESDRD
(PRED) P54854_Saccharo  ---------- ---------- ---------- -------MAS EQSSPEINAD
(PRED) P47185_Saccharo  ---------- ---------- ---------- -------MAS EQSSPEINAD
(PRED) P10870_Saccharo  MDPNSNSSSE TLRQEKQGFL DKALQRVKGI ALRRNNSNKD HTTDDTTGSI
(PRED) P43581_Saccharo  ---------- ---------- ---------- ---------- --------MV
(PRED) P23585_Saccharo  ---------- ---------- ---------- ---------- -MSEFATSRV
(PRED) O74849_Schizosa  ---------- ---------- ---------- ---------- ----------
(PRED) P15325_Emericel  ---------- ---------- ---------- ---------- ----------
(PRED) Q59TA4_Candida_  ---------- ---------- ---------- ---------- ----------
(PRED) Q59TK2_Candida_  ---------- ---------- ---------- ---------- ----------
(PRED) Q8L6Z8_Arabidop  ---------- ---------- ---------- ---------- ----------
(PRED) Q94AZ2_Arabidop  ---------- ---------- ---------- ---------- ----------
(PRED) Q8BFW9_Mus_musc  ---------- ---------- ---------- ---------- ----------
(PRED) Q7KWJ5_Plasmodi  ---------- ---------- ---------- ---------- ----------
(PRED) O44827_Caenorha  ---------- ---------- ---------- ---------- ----------
(PRED) P11166_Homo_sap  ---------- ---------- ---------- ---------- ----------
(PRED) P11167_Rattus_n  ---------- ---------- ---------- ---------- ----------
(PRED) P17809_Mus_musc  ---------- ---------- ---------- ---------- ----------
(PRED) P14142_Mus_musc  ---------- ---------- ---------- ---------- ----------
(PRED) P19357_Rattus_n  ---------- ---------- ---------- ---------- ----------
(PRED) P14246_Mus_musc  ---------- ---------- ---------- ---------- ----------
(PRED) P12336_Rattus_n  ---------- ---------- ---------- ---------- ----------
(PRED) P11168_Homo_sap  ---------- ---------- ---------- ---------- ----------
(PRED) P32037_Mus_musc  ---------- ---------- ---------- ---------- ----------
(PRED) Q07647_Rattus_n  ---------- ---------- ---------- ---------- ----------
(PRED) P43427_Rattus_n  ---------- ---------- ---------- ---------- ----------
(PRED) Q9JIF3_Mus_musc  ---------- ---------- ---------- ---------- ----------
(PRED) Q8K4S3_Rattus_n  MIPPAGS--- ---TPPGEAL IPSVAPQ--- DFWRSPISGY SGSVTRHISH

                         .........60 .........70 .........80 .........90 .........100
(PRED) Q8VZ80_Arabidop  ---------- -MTGATPENR TAPSPPPVKH VPESVLPAKP PKRNNYAFAC
(PRED) G1UB10_Candida_  ---------- ---------- -MSANIQALM KSYVNFDEHT SGSAARGILI
(PRED) Q5B0S0_Emericel  DIKGLFKPKA EQQEHSQATT PSRTDSIAEK DNGIIDDSPV KYLTWRSFIL
(PRED) P13181_Saccharo  LSKDSHLSAQ SQKYSNDELK AGESGSEGSQ SVPIEIPKKP MSEYVTVSLL
(PRED) P40886_Saccharo  IENSSHLSVP TVEENKDF-- ---SEYNGEE AEEVVVPEKP ASAYATVSIM
(PRED) P32465_Saccharo  YELESGRSKA MNTPEGKNES FHDNLSESQV QPAVAPPNTG KGVYVTVSIC
(PRED) P32466_Saccharo  ADLPSNSSQV MNMPEEKGV- --QDDFQAEA DQVLTNPNTG KGAYVTVSIC
(PRED) P39003_Saccharo  VDSASHSVLS TPSNKAERDE IKAYGEGEEH EPVVEIPKRP ASAYVTVSIM
(PRED) P39004_Saccharo  VDSASHSVLS TPSNKAERDE IKAYGEGEEH EPVVEIPKRP ASAYVTVSIM
(PRED) P32467_Saccharo  ESDSNSALST PSNKAERDDM KDFDENHEES NNYVEIPKKP ASAYVTVSIC
(PRED) P38695_Saccharo  YNDNLAQAKP VSSYISHEGP PKDELEELQK EVDKQLEKKS KSDLLFVSVC
(PRED) P54862_Saccharo  TMSNSNSAVG APSVKTEHGD SKNSLNLDAN EPPIDLPQKP LSAYTTVAIL
(PRED) P40885_Saccharo  TESNSNSVAN APSVKTEHND SKNSLNLDAT EPPIDLPQKP LSAYTTVAIL
(PRED) P39924_Saccharo  DGDVRDADIH VAPPVEKEWS DGFDDNEVIN GDNVEPPKRG LIGYLVIYLL
(PRED) P53631_Saccharo  IQDGPDADIH VAPPVEKEWS DGFDDNEVIN GDNVEPPKRG LIGYLVIYLL
(PRED) P54854_Saccharo  NLNSSAADVH VQPPGEKEWS DGFYDKEVIN GNTPDAPKRG FLGYLIIYLL
(PRED) P47185_Saccharo  NLNSSAADVH VQPPGEKEWS DGFYDKEVIN GNTPDAPKRG FLGYLIIYLL
(PRED) P10870_Saccharo  RTPTSLQRQN SDRQSNMTSV FTDDISTIDD NSILFSEPPQ KQSMMMSICV
(PRED) P43581_Saccharo  SSSVSILGTS AKASTSLSRK DEIKLTPETR EASLDIPYKP IIAYWTVMGL
```

```
(PRED) P23585_Saccharo ESGSQQTSIH  STPIVQKLET  DESPIQTKSE  YTNAELPAKP  IAAYWTVICL
(PRED) O74849_Schizosa ----------  ----------  ----------  ----------  -MAKILTIVM
(PRED) P15325_Emericel ----------  ----------  ------MSIL  ALVEDRPTPR  EVYNWRVYLL
(PRED) Q59TA4_Candida_ ----------  ---------MS  SKIERIFSGP  ALKINTYLDK  LPKIYNVFFI
(PRED) Q59TK2_Candida_ ----------  ---------MS  SKIERIFSGP  ALKINTYLDK  LPKIYNVFFI
(PRED) Q8L6Z8_Arabidop MGFDPENQSI  SSVGQVVGDS  SSGGITAEKE  PLLKENHSPE  NYSVLAAIPP
(PRED) Q94AZ2_Arabidop ----------  ----------  ----MTGGGF  ATSANGVEFE  AKITPIVIIS
(PRED) Q8BFW9_Mus_musc ---MVPVENT  EGPNLLNQKG  REAETEGSCG  ASGGGHPACA  GGPSMFTFLT
(PRED) Q7KWJ5_Plasmodi ----------  ----------  -MTKSSKDIC  SENEGKKNGK  SGFFSTSFKY
(PRED) O44827_Caenorha MSEKSRSDTS  ATASLSDSSK  SPSSYSTPGT  TTQKIIFPDG  KLTKCLAFSA
(PRED) P11166_Homo_sap ----------  ----------  ----------  ----MEPSSK  KLTGRLMLAV
(PRED) P11167_Rattus_n ----------  ----------  ----------  ----MEPSSK  KVTGRLMLAV
(PRED) P17809_Mus_musc ----------  ----------  ----------  ----MDPSSK  KVTGRLMLAV
(PRED) P14142_Mus_musc ----------  ----------  --MPSGFQQI  GSDDGEPPRQ  RVTGTLVLAV
(PRED) P19357_Rattus_n ----------  ----------  --MPSGFQQI  GSEDGEPPQQ  RVTGTLVLAV
(PRED) P14246_Mus_musc ----------  ----------  ----------  ------MSED  KITGTLAFTV
(PRED) P12336_Rattus_n ----------  ----------  ----------  ------MSED  KITGTLAFTV
(PRED) P11168_Homo_sap ----------  ----------  ----------  ------MTED  KVTGTLVFTV
(PRED) P32037_Mus_musc ----------  ----------  ----------  ------MGTT  KVTPSLVFAV
(PRED) Q07647_Rattus_n ----------  ----------  ----------  ------MGTA  KVTPSLVFAV
(PRED) P43427_Rattus_n ----------  ----------  ----------  MEKEDQEKTG  KLTLVLALAT
(PRED) Q9JIF3_Mus_musc ----------  ----------  -MSPEDPQET  QPLLRPPEAR  TPRGRRVFLA
(PRED) Q8K4S3_Rattus_n RANNFKRHPK  RRKYIRPS--  PPPPPNTPCP  IELVDFEDLH  PQRSFWELLF

                       .........110 .........120.........130.........140.........150
(PRED) Q8VZ80_Arabidop AILASMTSIL  LGYDIGVMSG  AM----IYIK  RD--------  L---------
(PRED) G1UB10_Candida_ GMFAAFGGFL  FGYDTGTISG  VLSM-DYVKA  RF--------  P------N--
(PRED) Q5B0S0_Emericel GIVVSMGGFI  FGYSTGQISG  FTTM-ADFKK  RF--------  AE-RQ-AN--
(PRED) P13181_Saccharo CLCVAFGGFM  FGWDTGTISG  FVVQ-TDFLR  RF--------  GM-KH-KD--
(PRED) P40886_Saccharo CLCMAFGGFM  SGWDTGTISG  FVNQ-TDFLR  RF--------  GNYSH-SK--
(PRED) P32465_Saccharo CVMVAFGGFI  FGWDTGTISG  FVAQ-TDFLR  RF--------  GM-KH-HD--
(PRED) P32466_Saccharo CVMVAFGGFV  FGWDTGTISG  FVAQ-TDFLR  RF--------  GM-KH-KD--
(PRED) P39003_Saccharo CIMIAFGGFV  FGWDTGTISG  FINQ-TDFIR  RF--------  GM-KH-KD--
(PRED) P39004_Saccharo CIMIAFGGFV  FGWDTGTISG  FINQ-TDFIR  RF--------  GM-KH-KD--
(PRED) P32467_Saccharo CLMVAFGGFV  FGWDTGTISG  FVAQ-TDFIR  RF--------  GM-KH-HD--
(PRED) P38695_Saccharo CLMVAFGGFV  FGWDTGTISG  FVRQ-TDFIR  RF--------  GS-TR-AN--
(PRED) P54862_Saccharo CLMIAFGGFI  FGWDTGTISG  FVNL-SDFIR  RF--------  GQ-KN-DK--
(PRED) P40885_Saccharo CLMIAFGGFI  FGWDTGTISG  FVNL-SDFIR  RF--------  GQ-KN-DK--
(PRED) P39924_Saccharo CYPISFGGFL  PGWDSGITAG  FINM-DNFKM  NF--------  GSYKH-ST--
(PRED) P53631_Saccharo CYPISFGGFL  PGWDSGITAG  FINM-DNFKM  NF--------  GSYKH-ST--
(PRED) P54854_Saccharo CYPVSFGGFL  PGWDSGITAG  FINM-DNFKM  NF--------  GSYKH-ST--
(PRED) P47185_Saccharo CYPVSFGGFL  PGWDSGITAG  FINM-DNFKM  NF--------  GSYKH-ST--
(PRED) P10870_Saccharo GVFVAVGGFL  FGYDTGLINS  ITSM-NYVKS  HV--------  A------P--
(PRED) P43581_Saccharo CLMIAFGGFI  FGWDTGTISG  FINQ-TDFKR  RF--------  GE-LQ-RD--
(PRED) P23585_Saccharo CLMIAFGGFV  FGWDTGTISG  FVNQ-TDFKR  RF--------  GQ-MK-SD--
(PRED) O74849_Schizosa LVFVSMAGWM  FGADTGSIGG  ITNM-RDFQS  RY--------  ADRYDPVT--
(PRED) P15325_Emericel AAVASFTSCM  IGYDSAFIGT  TLSL-QSFQN  EF--------  NW--------
(PRED) Q59TA4_Candida_ ASISTIAGMM  FGFDISSMSA  FIGA-EHYMR  YF--------  N---------
(PRED) Q59TK2_Candida_ ASISTIAGMM  FGFDISSMSA  FIGA-EHYMR  YF--------  N---------
(PRED) Q8L6Z8_Arabidop FLFPALGALL  FGYEIGATSC  AIMS-LKSPT  LS--------  G------I--
(PRED) Q94AZ2_Arabidop CIMAATGGLM  FGYDVGVSGG  VTSM-PDFLE  KFFPVVYRKV  VAGADKDS--
(PRED) Q8BFW9_Mus_musc SVTAAISGLL  VGYELGLISG  ALLQ-IRTLL  AL--------  ----------
(PRED) Q7KWJ5_Plasmodi VLSACIASFI  FGYQVSVLNT  IKNF-IVVEF  EW--------  -CKGEKDR--
(PRED) O44827_Caenorha FVITL-ASFQ  FGYHIGCVNA  PGGLITEWII  GSHKDLFDKE  ----------
(PRED) P11166_Homo_sap GGAVL-GSLQ  FGYNTGVINA  PQKVIEEFYN  QTWVHRYGES  ----------
(PRED) P11167_Rattus_n GGAVL-GSLQ  FGYNTGVINA  PQKVIEEFYN  QTWNHRYGES  ----------
(PRED) P17809_Mus_musc GGAVL-GSLQ  FGYNTGVINA  PQKVIEEFYN  QTWNHRYGEP  ----------
(PRED) P14142_Mus_musc FSAVL-GSLQ  FGYNIGVINA  PQKVIEQSYN  ATWLGRQGPG  GPDS------
(PRED) P19357_Rattus_n FSAVL-GSLQ  FGYNIGVINA  PQKVIEQSYN  ATWLGRQGPG  GPDS------
(PRED) P14246_Mus_musc FTAVL-SSFQ  FGYDIGVINA  PQEVIISHYR  HVLGVPLDDR  KAAINYDVNG
(PRED) P12336_Rattus_n FTAVL-GSFQ  FGYDIGVINA  PQEVIISHYR  HVLGVPLDDR  RATINYDING
(PRED) P11168_Homo_sap ITAVL-GSFQ  FGYDIGVINA  PQQVIISHYR  HVLGVPLDDR  KAINNYVINS
(PRED) P32037_Mus_musc TVATI-GSFQ  FGYNTGVINA  PETILKDFLN  YTLEERLEDL  ----------
(PRED) Q07647_Rattus_n TVATI-GSFQ  FGYNTGVINA  PETIIKDFLN  YTLEERLEDL  ----------
(PRED) P43427_Rattus_n FLAAFGSSFQ  YGYNVAAVNS  PSEFMQQFYN  DTYYDRNKEN  ----------
(PRED) Q9JIF3_Mus_musc SFAAALGPLS  FGFALGYSSP  AIPSLRRTAP  PA--------  ----------
(PRED) Q8K4S3_Rattus_n NGCILFG-IE  FSY-----AM  ETAYVTPVLL  QM--------  ----------

                       .........160 .........170.........180.........190.........200
(PRED) Q8VZ80_Arabidop ----------  ----------  -------KIN  DLQIGILAGS  LNIYSLIGSC
(PRED) G1UB10_Candida_ ----------  ----------  ----NKTDFT  SGESSLIVSI  LSVGTFVGSL
(PRED) Q5B0S0_Emericel ----------  ----------  ----GEYVFS  NVRNGLIVGL  LCIGTMIGAL
(PRED) P13181_Saccharo ----------  ----------  ----GTHYLS  NVRTGLIVAI  FNIGCAFGGI
(PRED) P40886_Saccharo ----------  ----------  ----NTYYLS  NVRTGLIVSI  FNVGSAIGCL
(PRED) P32465_Saccharo ----------  ----------  ----GSHYLS  KVRTGLIVSI  FNIGCAIGGI
```

```
(PRED) P32466_Saccharo ---------- ---------- ----GSYYLS KVRTGLIVSI FNIGCAIGGI
(PRED) P39003_Saccharo ---------- ---------- ----GTNYLS KVRTGLIVSI FNIGCAIGGI
(PRED) P39004_Saccharo ---------- ---------- ----GTNYLS KVRTGLIVSI FNIGCAIGGI
(PRED) P32467_Saccharo ---------- ---------- ----GTYYLS KVRTGLIVSI FNIGCAIGGI
(PRED) P38695_Saccharo ---------- ---------- ----GTTYLS DVRTGLMVSI FNIGCAIGGI
(PRED) P54862_Saccharo ---------- ---------- ----GTYYLS KVRMGLIVSI FNIGCAIGGI
(PRED) P40885_Saccharo ---------- ---------- ----GTYYLS KVRMGLIVSI FNIGCAIGGI
(PRED) P39924_Saccharo ---------- ---------- ----GEYYLS NVRMGLLVAM FSIGCAIGGL
(PRED) P53631_Saccharo ---------- ---------- ----GEYYLS NVRMGLLVAM FSIGCAIGGL
(PRED) P54854_Saccharo ---------- ---------- ----GEYYLS NVRMGLLVAM FSVGCSIGGV
(PRED) P47185_Saccharo ---------- ---------- ----GEYYLS NVRMGLLVAM FSVGCSIGGV
(PRED) P10870_Saccharo ---------- ---------- ----NHDSFT AQQMSILVSF LSLGTFFGAL
(PRED) P43581_Saccharo ---------- ---------- ----GSFQLS DVRTGLIVGI FNIGCALGGL
(PRED) P23585_Saccharo ---------- ---------- ----GTYYLS DVRTGLIVGI FNIGCAFGGL
(PRED) O74849_Schizosa ---------- ---------- ----DTYSYS SARQGLLVGM VNTGTTVGCL
(PRED) P15325_Emericel ---------- ---------- -----ESLNT DLISANIVSL YQAGAFFGAL
(PRED) Q59TA4_Candida_ ---------- ---------- -------SPG SDIQGFITSS MALGSFFGSI
(PRED) Q59TK2_Candida_ ---------- ---------- -------SPG SNIQGFITSS MALGSFFGSI
(PRED) Q8L6Z8_Arabidop ---------- ---------- ----SWYDLS SVDVGIITSG SLYGALIGSI
(PRED) Q94AZ2_Arabidop ---------- ---------- ----NYCKYD NQGLQLFTSS LYLAGLTATF
(PRED) Q8BFW9_Mus_musc ---------- ---------- ---------T CHEQEMVVSS LLIGAFLASL
(PRED) Q7KWJ5_Plasmodi ---------- ---------- ----LNCSNN TIQSSFLLAS VFIGAVLGCG
(PRED) O44827_Caenorha ---------- ---------- ----LSRENA DLAWSVAVSV FAVGGMIGGL
(PRED) P11166_Homo_sap ---------- ---------- ----ILPTTL TTLWSLSVAI FSVGGMIGSF
(PRED) P11167_Rattus_n ---------- ---------- ----IPSTTL TTLWSLSVAI FSVGGMIGSF
(PRED) P17809_Mus_musc ---------- ---------- ----IPSTTL TTLWSLSVAI FSVGGMIGSF
(PRED) P14142_Mus_musc ---------- ---------- ----IPQGTL TTLWALSVAI FSVGGMISSF
(PRED) P19357_Rattus_n ---------- ---------- ----IPQGTL TTLWALSVAI FSVGGMISSF
(PRED) P14246_Mus_musc TDTPLTVTPA Y-TTPAPWDE EETEGSAHIV TMLWSLSVSS FAVGGMVASF
(PRED) P12336_Rattus_n TDTPLIVTPA --HTTPDAWE EETEGSAHIV TMLWSLSVSS FAVGGMVASF
(PRED) P11168_Homo_sap TDELPTISYS MNPKPTPWAE EETVAAAQLI TMLWSLSVSS FAVGGMTASF
(PRED) P32037_Mus_musc ---------- ---------- ----PSEGLL TALWSLCVAI FSVGGMIGSF
(PRED) Q07647_Rattus_n ---------- ---------- ----PREGLL TTLWSLCVAI FSVGGMIGSF
(PRED) P43427_Rattus_n ---------- ---------- ----IESFTL TLLWSLTVSM FPFGGFIGSL
(PRED) Q9JIF3_Mus_musc ---------- ---------- ------LRLG DNAASWFGAV VTLGAAAGGI
(PRED) Q8K4S3_Rattus_n ---------- ---------- -------GLP DQLYSLVWFI SPILGFLLQP

                       .........210 .........220.........230.........240.........250
(PRED) Q8VZ80_Arabidop AAGRTSDW-- --IGRRYTIV LAGA-IFFAG AILMGLSP-- -NYAFLMFGR
(PRED) G1UB10_Candida_ IAPLFSDR-- --IGRRWTLI LSTLIVFNLG VLLQTVAT-- -EKKLLIAGR
(PRED) Q5B0S0_Emericel VAAPIADR-- --IGRKLSMS FWSI-IHIVG IIIQIATDS- -NWVQIAMGR
(PRED) P13181_Saccharo ILSKGGDM-- --YGRKKGLS IVVS-VYIVG IIIQIASIN- -KWYQYFIGR
(PRED) P40886_Saccharo FLSKLGDI-- --YGRCMGLI IVIV-VYMVG IVIQIASID- -KWYQYFIGR
(PRED) P32465_Saccharo VLAKLGDM-- --YGRRIGLI VVVV-IYTIG IIIQIASIN- -KWYQYFIGR
(PRED) P32466_Saccharo ILAKLGDM-- --YGRKMGLI VVVV-IYIIG IIIQIASIN- -KWYQYFIGR
(PRED) P39003_Saccharo ILSKLGDM-- --YGRKVGLI VVVV-IYIIG IIIQIASIN- -KWYQYFIGR
(PRED) P39004_Saccharo ILSKLGDM-- --YGRKVGLI VVVV-IYIIG IIIQIASIN- -KWYQYFIGR
(PRED) P32467_Saccharo ILAKLGDM-- --YGRKMGLI VVVV-IYIIG IIIQIASIN- -KWYQYFIGR
(PRED) P38695_Saccharo VLSKLGDM-- --YGRKIGLM TVVV-IYSIG IIIQIASID- -KWYQYFIGR
(PRED) P54862_Saccharo VLSKVGDI-- --YGRRIGLI TVTA-IYVVG ILIQITSIN- -KWYQYFIGR
(PRED) P40885_Saccharo VLSKVGDI-- --YGRRIGLI TVTA-IYVVG ILIQITSIN- -KWYQYFIGR
(PRED) P39924_Saccharo IFARLADT-- --LGRRLAIV IVVL-VYMVG AIIQISSNH- -KWYQYFVGK
(PRED) P53631_Saccharo IFARLADT-- --LGRRLAIV IVVL-VYMVG AIIQISSNH- -KWYQYFVGK
(PRED) P54854_Saccharo AFARLADT-- --LGRRLAIV IVVL-VYMVG AIIQISSNH- -KWYQYFVGK
(PRED) P47185_Saccharo AFARLADT-- --LGRRLAIV IVVL-VYMVG AIIQISSNH- -KWYQYFVGK
(PRED) P10870_Saccharo TAPFISDS-- --YGRKPTII FSTIFIFSIG NSLQVGAG-- -GITLLIVGR
(PRED) P43581_Saccharo TLGRLGDI-- --YGRKIGLM CVIL-VYYVG IVIQIASSD- -KWYQYFIGR
(PRED) P23585_Saccharo TLGRLGDM-- --YGRRIGLM CVVL-VYIVG IVIQIASSD- -KWYQYFIGR
(PRED) O74849_Schizosa LSSPLGDR-- --FGKRKCIM GWTL-VYITG VIVQLTTIP- -SWVQMMVAK
(PRED) P15325_Emericel FAYPIGHF-- --WGRRWGLM FSAL-IFFLG AGMMLGANGD RGLGLIYGGR
(PRED) Q59TA4_Candida_ ASSFVSEP-- --FGRRLSLL TCAF-FWMVG AAIQSSVQ-- -NRAQLIIGR
(PRED) Q59TK2_Candida_ ASSFVSEP-- --FGRRLSLL TCAF-FWMVG AAIQSSVQ-- -NRAQLIIGR
(PRED) Q8L6Z8_Arabidop VAFSVADI-- --IGRRKELI LAAF-LYLVG AIVTVVAP-- -VFSILIIGR
(PRED) Q94AZ2_Arabidop FASYTTRT-- --LGRRLTML IAGV-FFIIG VALNAGAQ-- -DLAMLIAGR
(PRED) Q8BFW9_Mus_musc TGGVLIDR-- --YGRRLAII LSSC-LLGLG SLVLIMSL-- -SYTLLIMGR
(PRED) Q7KWJ5_Plasmodi FSGYLVQ--- --FGRRLSLL IIYN-FFFLV SILTSITH-- -HFHTILFAR
(PRED) O44827_Caenorha SSGWLADK-- --VGRRGALF YNNL-LALAA AALMGLAKSV GAYPMVILGR
(PRED) P11166_Homo_sap SVGLFVNR-- --FGRRNSML MMNL-LAFVS AVLMGFSKLG KSFEMLILGR
(PRED) P11167_Rattus_n SVGLFVNR-- --FGRRNSML MMNL-LAFVS AVLMGFSKLG KSFEMLILGR
(PRED) P17809_Mus_musc SVGLFVNR-- --FGRRNSML MMNL-LAFVA AVLMGFSKLG KSFEMLILGR
(PRED) P14142_Mus_musc LIGIISQW-- --LGRKRAML ANNV-LAVLG GALMGLANAA ASYEILILGR
(PRED) P19357_Rattus_n LIGIISQW-- --LGRKRAML ANNV-LAVLG GALMGLANAA ASYEILILGR
(PRED) P14246_Mus_musc FGGWLGDK-- --LGRIKAML AANS-LSLTG ALLMGCSKFG PAHALIIAGR
(PRED) P12336_Rattus_n FGGWLGDK-- --LGRIKAML AANS-LSLTG ALLMGCSKFG PAHALIIAGR
(PRED) P11168_Homo_sap FGGWLGDT-- --LGRIKAML VANI-LSLVG ALLMGFSKLG PSHILIIAGR
```

```
(PRED) P32037_Mus_musc SVGLFVNR-- --FGRRNSML LVNL-LAIIA GCLMGFAKIA ESVEMLILGR
(PRED) Q07647_Rattus_n SVGLFVNR-- --FGRRNSML LVNL-IAILG GCLMGFAKIA ESVEMLILGR
(PRED) P43427_Rattus_n MVGFLVNN-- --LGRKGALL FNNI-FSILP AILMGCSKIA KSFEIIIASR
(PRED) Q9JIF3_Mus_musc LGGWLLDR-- --AGRKLSLL LCTV-PFVTG FAVITAAR-- -DVWMLLGGR
(PRED) Q8K4S3_Rattus_n LLGAWSDRCT SRFGRRRPFI LVLAIGALLG LSLLLNGRDI GMALADTATN

                       .........260 .........270.........280.........290.........300
(PRED) Q8VZ80_Arabidop FIAGIGVGYA LMIAPVYTAE VSPASSRGFL NSF--PEVFI NAGIMLGYVS
(PRED) G1UB10_Candida_ AIAGTGVGLI SSVIPNYISE TTPKWARGAV TAS--YQWMI TWGLLIAACA
(PRED) Q5B0S0_Emericel WVAGLGVGAL SSVVPMYQSE AAPRQVRGAM ISA--FQLFV AFGIFISYII
(PRED) P13181_Saccharo IISGLGVGGI AVLCPMLISE IAPKHLRGTL VSC--YQLMI TAGIFLGYCT
(PRED) P40886_Saccharo IIAGIGAGSI SVLAPMLISE TAPKHIRGTL LAC--WQLMV TFAIFLGYCT
(PRED) P32465_Saccharo IISGLGVGGI TVLSPMLISE VAPSEMRGTL VSC--YQVMI TLGIFLGYCT
(PRED) P32466_Saccharo IISGLGVGGI AVLSPMLISE VAPKEMRGTL VSC--YQLMI TLGIFLGYCT
(PRED) P39003_Saccharo IISGLGVGGI AVLSPMLISE VSPKHLRGTL VSC--YQLMI TAGIFLGYCT
(PRED) P39004_Saccharo IISGLGVGGI AVLSPMLISE VSPKHLRGTL VSC--YQLMI TAGIFLGYCT
(PRED) P32467_Saccharo IISGLGVGGI AVLSPMLISE VSPKHIRGTL VSC--YQLMI TLGIFLGYCT
(PRED) P38695_Saccharo IISGLGVGGI TVLAPMLISE VSPKQLRGTL VSC--YQLMI TFGIFLGYCT
(PRED) P54862_Saccharo IISGLGVGGI AVLSPMLISE VAPKHIRGTL VQL--YQLMG TMGIFLGYCT
(PRED) P40885_Saccharo IISGLGVGGI AVLSPMLISE VAPKQIRGTL VQL--YQLMC TMGIFLGYCT
(PRED) P39924_Saccharo IIYGLGAGGC SVLCPMLLSE IAPTDLRGGL VSL--YQLNM TFGIFLGYCS
(PRED) P53631_Saccharo IIYGLGAGGC SVLCPMLLSE IAPTDLRGGL VSL--YQLNM TFGIFLGYCS
(PRED) P54854_Saccharo IIYGLGAGGC SVLCPMLLSE IAPTDLRGGL VSL--YQLNM TFGIFLGYCS
(PRED) P47185_Saccharo IIYGLGAGGC SVLCPMLLSE IAPTDLRGGL VSL--YQLNM TFGIFLGYCS
(PRED) P10870_Saccharo VISGIGIGAI SAVVPLYQAE ATHKSLRGAI IST--YQWAI TWGLLVSSAV
(PRED) P43581_Saccharo IVSGMGVGGV AVLSPTLISE ISPKHLRGTC VSF--YQLMI TLGIFLGYCT
(PRED) P23585_Saccharo IISGMGVGGI AVLSPTLISE TAPKHIRGTC VSF--YQLMI TLGIFLGYCT
(PRED) O74849_Schizosa IWTGLGIGAL SVIAPGYQSE SSPPHIRGAI VTT--YQLFI TLGIFIAACI
(PRED) P15325_Emericel VLAGIGVGAG SNICPIYISE MAPPAIRGRL VGV--YELGW QIGGVVGFWI
(PRED) Q59TA4_Candida_ IISGIGVGFG SAVAPVYGAE LAPRKIRGLI GGM--FQFFV TLGIMIMFYL
(PRED) Q59TK2_Candida_ IISGIGVGFG SAVAPVYGAE LAPRKIRGLI GGM--FQFFV TLGIMIMFYL
(PRED) Q8L6Z8_Arabidop VTYGMGIGLT MHAAPMYIAE TAPSQIRGRM ISL--KEFST VLGMVGGYGI
(PRED) Q94AZ2_Arabidop ILLGCGVGFA NQAVPLFLSE IAPTRIRGGL NIL--FQLNV TIGILFANLV
(PRED) Q8BFW9_Mus_musc VAIGVSISLS SIATCVYIAE IAPQHRRGLL VSL--NELMI VTGILFAYIS
(PRED) Q7KWJ5_Plasmodi LLSGFGIGLV TVSVPMYISE MTHKDKKGAY GVM--HQLFI TFGIFVAVML
(PRED) O44827_Caenorha LIIGLNCGFS SALVPMFLTE ISPNNLRGML GSL--HQLLV TIAILVSQIF
(PRED) P11166_Homo_sap FIIGVYCGLT TGFVPMYVGE VSPTALRGAL GTL--HQLGI VVGILIAQVF
(PRED) P11167_Rattus_n FIIGVYCGLT TGFVPMYVGE VSPTALRGAL GTL--HQLGI VVGILIAQVF
(PRED) P17809_Mus_musc FIIGVYCGLT TGFVPMYVGE VSPTALRGAL GTL--HQLGI VVGILIAQVF
(PRED) P14142_Mus_musc FLIGAYSGLT SGLVPMYVGE IAPTHLRGAL GTL--NQLAI VIGILVAQVL
(PRED) P19357_Rattus_n FLIGAYSGLT SGLVPMYVGE IAPTHLRGAL GTL--NQLAI VIGILVAQVL
(PRED) P14246_Mus_musc SVSGLYCGLI SGLVPMYIGE IAPTTLRGAL GTL--HQLAL VTGILISQIA
(PRED) P12336_Rattus_n SVSGLYCGLI SGLVPMYIGE IAPTTLRGAL GTL--HQLAL VTGILISQIA
(PRED) P11168_Homo_sap SISGLYCGLI SGLVPMYIGE IAPTALRGAL GTF--HQLAI VTGILISQII
(PRED) P32037_Mus_musc LLIGIFCGLC TGFVPMYIGE VSPTALRGAF GTL--NQLGI VVGILVAQIF
(PRED) Q07647_Rattus_n LIIGIFCGLC TGFVPMYIGE VSPTALRGAF GTL--NQLGI VVGILVAQVF
(PRED) P43427_Rattus_n LLVGICAGIS SNVVPMYLGE LAPKNLRGAL GVV--PQLFI TVGILVAQLF
(PRED) Q9JIF3_Mus_musc LLTGLACGVA SLVAPVYISE IAYPAVRGLL GSC--VQLMV VTGILLAYVA
(PRED) Q8K4S3_Rattus_n HKWGILLTVC GVVLMDFSAD SADNPSHAYM MDVCGPVDQD RGLNIHALMA

                       .........310 .........320.........330.........340.........350
(PRED) Q8VZ80_Arabidop NLAFSNLP-- LK-------- -VGWR-LMLG IGAVPSVI-- LAIGVLAMPE
(PRED) G1UB10_Candida_ NKGSQGRK-- DS-------- -GSYR-IPIG IQFLWALI-- LGIGFLFLPE
(PRED) Q5B0S0_Emericel NYGTESIQ-- ST-------- -ASWR-ITMG IGFAWPLI-- LGLGALFLPE
(PRED) P13181_Saccharo NYGTKSYS-- NS-------- -VQWR-VPLG LCFAWSLF-- MIGALTLVPE
(PRED) P40886_Saccharo NYGTKTYS-- NS-------- -VQWR-VPLG LCFAWAII-- MIGGMTFVPE
(PRED) P32465_Saccharo NFGTKNYS-- NS-------- -VQWR-VPLG LCFAWALF-- MIGGMMFVPE
(PRED) P32466_Saccharo NFGTKNYS-- NS-------- -VQWR-VPLG LCFAWALF-- MIGGMTFVPE
(PRED) P39003_Saccharo NFGTKNYS-- NS-------- -VQWR-VPLG LCFAWALF-- MIGGMTFVPE
(PRED) P39004_Saccharo NFGTKNYS-- NS-------- -VQWR-VPLG LCFAWALF-- MIGGMTFVPE
(PRED) P32467_Saccharo NYGTKTYT-- NS-------- -VQWR-VPLG LGFAWALF-- MIGGMTFVPE
(PRED) P38695_Saccharo NFGTKNYS-- NS-------- -VQWR-VPLG LCFAWSIF-- MIVGMTFVPE
(PRED) P54862_Saccharo NYGTKNYH-- NA-------- -TQWR-VGLG LCFAWATF-- MVSGMMFVPE
(PRED) P40885_Saccharo NYGTKNYH-- NA-------- -TQWR-VGLG LCFAWTTF-- MVSGMMFVPE
(PRED) P39924_Saccharo VYGTRKYD-- NT-------- -AQWR-VPLG LCFLWALI-- IIIGMLLVPE
(PRED) P53631_Saccharo VYGTRKYD-- NT-------- -AQWR-VPLG LCFLWTLI-- IIIGMLLVPE
(PRED) P54854_Saccharo VYGTRKYS-- NT-------- -AQWR-IPVG LCFLWALI-- IIVGMLLVPE
(PRED) P47185_Saccharo VYGTRKYS-- NT-------- -AQWR-IPVG LCFLWALI-- IIVGMLLVPE
(PRED) P10870_Saccharo SQGTHARN-- DA-------- -SSYR-IPIG LQYVWSSF-- LAIGMFFLPE
(PRED) P43581_Saccharo NYGTKKYS-- NS-------- -IQWR-VPLG LCFAWAIF-- MVIGMVMVPE
(PRED) P23585_Saccharo NYGTKDYS-- NS-------- -VQWR-VPLG LNFAFAIF-- MIAGMLMVPE
(PRED) O74849_Schizosa NMGTHKYTTH PE-------- -AQWR-VPIG INLLWGIL-- MFFGMLFLPE
(PRED) P15325_Emericel NYGVDETLAP SH-------- -KQWI-IPFA VQLIPAGL-- LIIGALLIRE
(PRED) Q59TA4_Candida_ SFGLGHIN-- GV-------- -ASFR-IAWG LQIVPGLC-- LFLGCFFIPE
(PRED) Q59TK2_Candida_ SFGLGHIN-- GV-------- -ASFR-IAWG LQIVPGLC-- LFLGCFFIPE
```

```
(PRED) Q8L6Z8_Arabidop GSLWIT----  VI--------  -SGWR-YMYA  TILPFPVI--  MGTGMCWLPA
(PRED) Q94AZ2_Arabidop NYGTAKIK--  GG--------  -WGWR-LSLG  LAGIPALL--  LTVGALLVTE
(PRED) Q8BFW9_Mus_musc NYAFANI---  -S--------  -NGWK-YMFG  LVIPLGVL--  QAIAMYFLPP
(PRED) Q7KWJ5_Plasmodi GLAMGEGPKA  DSTEPLTSFA  KLWWR-LMFL  FPSVISLIGI  LALVVFFKEE
(PRED) O44827_Caenorha GLPHLLGT--  GD--------  --RWP-LIFA  FTVVPAVL--  QLALLMLCPE
(PRED) P11166_Homo_sap GLDSIMGN--  KD--------  --LWP-LLLS  IIFIPALL--  QCIVLPFCPE
(PRED) P11167_Rattus_n GLDSIMGN--  AD--------  --LWP-LLLS  VIFIPALL--  QCILLPFCPE
(PRED) P17809_Mus_musc GLDSIMGN--  AD--------  --LWP-LLLS  VIFIPALL--  QCILLPFCPE
(PRED) P14142_Mus_musc GLESMLGT--  AT--------  --LWP-LLLA  LTVLPALL--  QLILLPFCPE
(PRED) P19357_Rattus_n GLESMLGT--  AT--------  --LWP-LLLA  ITVLPALL--  QLLLLPFCPE
(PRED) P14246_Mus_musc GLSFILGN--  QD--------  --HWH-ILLG  LSAVPALL--  QCLLLLFCPE
(PRED) P12336_Rattus_n GLSFILGN--  QD--------  --YWH-ILLG  LSAVPALL--  QCLLLLFCPE
(PRED) P11168_Homo_sap GLEFILGN--  YD--------  --LWH-ILLG  LSGVRAIL--  QSLLLFFCPE
(PRED) P32037_Mus_musc GLDFILGS--  EE--------  --LWP-GLLG  LTIIPAIL--  QSAALPFCPE
(PRED) Q07647_Rattus_n GLDFILGS--  EE--------  --LWP-GLLG  LTIIPAIL--  QSAALPFCPE
(PRED) P43427_Rattus_n GLRSVLAS--  EE--------  --GWP-ILLG  LTGVPAGL--  QLLLLPFFPE
(PRED) Q9JIF3_Mus_musc GWVL------  ----------  --EWR-WLAV  LGCVPPTL--  MLLLMCYMPE
(PRED) Q8K4S3_Rattus_n GLGGGFGYVV  GG--------  -IHWDKTSFG  RALGGQLRVI  YIFTAITLSV

                       .........360 .........370.........380.........390.........400
(PRED) Q8VZ80_Arabidop SPRWLVMQGR  L---------  GDAKRVLDKT  SD-SPTEATL  RLEDIKHAAG
(PRED) G1UB10_Candida_ TPRYWVSKSE  E---------  TKAKDSLRRI  RN-LPVDHPD  ----------
(PRED) Q5B0S0_Emericel SPRYAYRLGR  I---------  DEARKVMAKL  YG-VEVNHRV  ----------
(PRED) P13181_Saccharo SPRYLCEVNK  V---------  EDAKRSIAKS  NK-VSPEDPA  ----------
(PRED) P40886_Saccharo SPRFLVQVGK  I---------  EQAKASFAKS  NK-LSVDDPA  ----------
(PRED) P32465_Saccharo SPRYLVEAGR  I---------  DEARASLAKV  NK-CPPDHPY  ----------
(PRED) P32466_Saccharo SPRYLVEAGQ  I---------  DEARASLSKV  NK-VAPDHPF  ----------
(PRED) P39003_Saccharo SPRYLAEVGK  I---------  EEAKRSIAVS  NK-VAVDDPS  ----------
(PRED) P39004_Saccharo SPRYLAEVGK  I---------  EEAKRSIAVS  NK-VAVDDPS  ----------
(PRED) P32467_Saccharo SPRYLVEVGK  I---------  EEAKRSIALS  NK-VSADDPA  ----------
(PRED) P38695_Saccharo SPRYLVEVGK  I---------  EEAKRSLARA  NK-TTEDSPL  ----------
(PRED) P54862_Saccharo SPRYLIEVGK  D---------  EEAKRSLSKS  NK-VSVDDPA  ----------
(PRED) P40885_Saccharo SPRYLIEVGK  D---------  EEAKRSLSKS  NK-VSVDDPA  ----------
(PRED) P39924_Saccharo SPRYLIECER  H---------  EEARASIAKI  NK-VSPEDPW  ----------
(PRED) P53631_Saccharo SPRYLIECER  H---------  EEARASIAKI  NK-VSPEDPW  ----------
(PRED) P54854_Saccharo SPRYLIECER  H---------  EEACVSIAKI  NK-VSPEDPW  ----------
(PRED) P47185_Saccharo SPRYLIECER  H---------  EEACVSIAKI  DK-VSPEDPW  ----------
(PRED) P10870_Saccharo SPRYYVLKDK  L---------  DEAAKSLSFL  RG-VPVHDSG  ----------
(PRED) P43581_Saccharo SPRYLVEKGK  Y---------  EEARRSLAKS  NK-VTVTDPG  ----------
(PRED) P23585_Saccharo SPRFLVEKGR  Y---------  EDAKRSLAKS  NK-VTIEDPS  ----------
(PRED) O74849_Schizosa SPRYLAVKGR  N---------  EECMKILTRN  AG-LPADHPI  ----------
(PRED) P15325_Emericel SPRWLFLRGN  R---------  EKGIETLAWI  RN-LPADHIY  ----------
(PRED) Q59TA4_Candida_ SPRWLAKQGQ  W---------  EAAEEIVAKI  QAHGDRENPD  ----------
(PRED) Q59TK2_Candida_ SPRWLAKQGQ  W---------  EAAEEIVAKI  QAHGDRENPD  ----------
(PRED) Q8L6Z8_Arabidop SPRWLLLRAL  QGQGNGENLQ  QAAIRSLCRL  RG--SVIADS  ----------
(PRED) Q94AZ2_Arabidop TPNSLVERGR  L---------  DEGKAVLRRI  RGTDN-----  ----------
(PRED) Q8BFW9_Mus_musc SPRFLVMKGQ  E---------  ESAGKVLRKL  RV----ISD-  ----------
(PRED) Q7KWJ5_Plasmodi TPYFLFEKGR  I---------  EESKNILKKI  YE-----TDN  ----------
(PRED) O44827_Caenorha SPKYTMAVRG  Q--------R  NEAESALKKL  RDTED-----  ----------
(PRED) P11166_Homo_sap SPRFLLINRN  E--------E  NRAKSVLKKL  RGTAD-----  ----------
(PRED) P11167_Rattus_n SPRFLLINRN  E--------E  NRAKSVLKKL  RGTAD-----  ----------
(PRED) P17809_Mus_musc SPRFLLINRN  E--------E  NRAKSVLKKL  RGTAD-----  ----------
(PRED) P14142_Mus_musc SPRYLYIIRN  L--------E  GPARKSLKRL  TGWAD-----  ----------
(PRED) P19357_Rattus_n SPRYLYIIRN  L--------E  GPARKSLKRL  TGWAD-----  ----------
(PRED) P14246_Mus_musc SPRYLYIKLE  E--------E  VRAKKSLKRL  RGTED-----  ----------
(PRED) P12336_Rattus_n SPRYLYLNLE  E--------E  VRAKKSLKRL  RGTED-----  ----------
(PRED) P11168_Homo_sap SPRYLYIKLD  E--------E  VKAKQSLKRL  RGYDD-----  ----------
(PRED) P32037_Mus_musc SPRFLLINKK  E--------E  DQATEILQRL  WGTSD-----  ----------
(PRED) Q07647_Rattus_n SPRFLLINRK  E--------E  DQATEILQRL  WGTPD-----  ----------
(PRED) P43427_Rattus_n SPRYLLIQKK  N--------E  SAAEKALQTL  RGWKD-----  ----------
(PRED) Q9JIF3_Mus_musc TPRFLLTQHQ  Y---------  QEAMAALRFL  WG--------  ----------
(PRED) Q8K4S3_Rattus_n TTVFTLVSIP  ERPL---RPL  GEKRTAMKSP  SLPLPPSPPV  LLEEGAGDTL

                       .........410 .........420.........430.........440.........450
(PRED) Q8VZ80_Arabidop IPADCHDDVV  QVSRRNSHGE  GVWRELLIRP  --TPAV--RR  VMIAAIGIHF
(PRED) G1UB10_Candida_ LVSEYDDIKA  NFDFESKYAT  SSWTQVFKN-  --VNKQ--HH  RLFTGVAIQA
(PRED) Q5B0S0_Emericel VVQEMKDMKD  KLEEERAAGV  APWHEVVTG-  ---PRM--LY  RTLLGIALQS
(PRED) P13181_Saccharo VQAELDLIMA  GIEAEKLAGN  ASWGELFST-  --KTKV--FQ  RLLMGVFVQM
(PRED) P40886_Saccharo VVAEIDLLVA  GVEAEEAMGT  ASWKELFSR-  --KTKV--FQ  RLTMTVMINS
(PRED) P32465_Saccharo IQYELETIEA  SVEEMRAAGT  ASWGELFTG-  --KPAM--FQ  RTMMGIMIQS
(PRED) P32466_Saccharo IQQELEVIEA  SVEEARAAGS  ASWGELFTG-  --KPAM--FK  RTMMGIMIQS
(PRED) P39003_Saccharo VLAEVEAVLA  GVEAEKLAGN  ASWGELFSS-  --KTKV--LQ  RLIMGAMIQS
(PRED) P39004_Saccharo VLAEVEAVLA  GVEAEKLAGN  ASWGELFSS-  --KTKV--LQ  RLIMGAMIQS
(PRED) P32467_Saccharo VMAEVEVVQA  TVEAEKLAGN  ASWGEIFST-  --KTKV--FQ  RLIMGAMIQS
(PRED) P38695_Saccharo VTLEMENYQS  SIEAERLAGS  ASWGELVTG-  --KPQM--FR  RTLMGMMIQS
```

```
(PRED) P54862_Saccharo LLVEYDTIKA GIELEKLAGN ASWSELLST- --KTKV--FQ RVLMGVMIQS
(PRED) P40885_Saccharo LLAEYDTIKA GIELEKLAGN ASWSELLST- --KTKV--FQ RVLMGVMIQS
(PRED) P39924_Saccharo VLKQADEINA GVLAQRELGE ASWKELFSV- --KTKV--LQ RLITGILVQT
(PRED) P53631_Saccharo VLKQADEINA GVLAQRELGE ASWKELFSV- --KTKV--LQ RLITGILVQT
(PRED) P54854_Saccharo VLKQADEINA GVLAQRELGE ASWKELFSV- --KTKV--LQ RLITGILVQT
(PRED) P47185_Saccharo VLKQADEINA GVLAQRELGE ASWKELFSV- --KTKV--LQ RLITGILVQT
(PRED) P10870_Saccharo LLEELVEIKA TYDYEASFGS SNFIDCFISS KSRPKQ--TL RMFTGIALQA
(PRED) P43581_Saccharo VVFEFDTIVA NMELERAVGN ASWHELFSN- --KGAI--LP RVIMGIVIQS
(PRED) P23585_Saccharo IVAEMDTIMA NVETERLAGN ASWGELFSN- --KGAI--LP RVIMGIMIQS
(PRED) O74849_Schizosa MQKEYNAIQA DVEAELAGGP CSWPQIFS-- ---NEI--RY RTLLGMGVMA
(PRED) P15325_Emericel MVEEINMIEQ SLEQQRVKIG LGFWKPFKAA WTNKRI--LY RLFLGSMLFL
(PRED) Q59TA4_Candida_ VLIEISEIKD QLLLEESSKQ IGYATLFT-- ---KKY--IQ RTFTAIFAQI
(PRED) Q59TK2_Candida_ VLIEISEIKD QLLLEESSKQ IGYATLFT-- ---KKY--IQ RTFTAIFAQI
(PRED) Q8L6Z8_Arabidop AAEQVNEILA ELSLVGEDKE ATFGELFR-- ---GKC--LK ALTIAGGLVL
(PRED) Q94AZ2_Arabidop VEPEFADLLE ASRLAKEV-K HPFRNLLQ-- ---RRN--RP QLVIAVALQI
(PRED) Q8BFW9_Mus_musc TTEELTLIKS SLKDEYQY-- -SFWDLFRS- --KDNM--RT RILIGLTLVF
(PRED) Q7KWJ5_Plasmodi VDEPLNAIKE AVEQNESAKK NSLSLLSALK --IPSY--RY VIILGCLLSG
(PRED) O44827_Caenorha VSTEIEAMQE EATAAGVQEK PKMGDMFK-- ---GAL--LW PMSIAIMMML
(PRED) P11166_Homo_sap VTHDLQEMKE ESRQMMREKK VTILELFRS- ---PAY--RQ PILIAVVLQL
(PRED) P11167_Rattus_n VTRDLQEMKE EGRQMMREKK VTILELFRS- ---PAY--RQ PILIAVVLQL
(PRED) P17809_Mus_musc VTRDLQEMKE EGRQMMREKK VTILELFRS- ---PAY--RQ PILIAVVLQL
(PRED) P14142_Mus_musc VSDALAELKD EKRKLERERP MSLLQLLGS- ---RTH--RQ PLIIAVVLQL
(PRED) P19357_Rattus_n VSDALAELKD EKRKLERERP LSLLQLLGS- ---RTH--RQ PLIIAVVLQL
(PRED) P14246_Mus_musc VTKDINEMKK EKEEASTEQK VSVIQLFTD- ---ANY--RQ PILVALMLHM
(PRED) P12336_Rattus_n ITKDINEMRK EKEEASTEQK VSVIQLFTD- ---PNY--RQ PIVVALMLHL
(PRED) P11168_Homo_sap VTKDINEMRK EREEASSEQK VSIIQLFTN- ---SSY--RQ PILVALMLHV
(PRED) P32037_Mus_musc VVQEIQEMKD ESVRMSQEKQ VTVLELFRS- ---PNY--VQ PLLISIVLQL
(PRED) Q07647_Rattus_n VIQEIQEMKD ESIRMSQEKQ VTVLELFKS- ---PSY--FQ PLLISVVLQL
(PRED) P43427_Rattus_n VDMEMEEIRK EDEAEKAAGF ISVWKLFRM- ---QSL--RW QLISTIVLMA
(PRED) Q9JIF3_Mus_musc SEEGWEEPPV GAEHQGF--- -QLALLRR-- ---PGI--YK PLIIGISLMV
(PRED) Q8K4S3_Rattus_n PSTTATSLYA SFSSPISPPS PLTPKYGSFI SRDSSLTGIN EFASSFGTSN

                       .........460 .........470 .........480 .........490 .........500
(PRED) Q8VZ80_Arabidop FQQASGIDAV VLFSPRIFKT AGLKTDHQQL LATVAVGVVK TSFILVA-TF
(PRED) G1UB10_Candida_ LQQLTGINFI FYYGTQFFKR SGI---EDPF LIQLATNIVN VGMTVPG-II
(PRED) Q5B0S0_Emericel LQQLSGANFI FYYGNSIFTS TGL---NNSY VTQIILGAVN FGMTLPG-LY
(PRED) P13181_Saccharo FQQLTGNNYF FYYGTVIFKS VGL---DDSF ETSIVIGVVN FASTFFS-LW
(PRED) P40886_Saccharo LQQLTGDNYF FYYGTTIFKS VGM---NDSF ETSIVLGIVN FASCFFS-LY
(PRED) P32465_Saccharo LQQLTGDNYF FYYGTIVFQA VGL---SDSF ETSIVFGVVN FFSTCCS-LY
(PRED) P32466_Saccharo LQQLTGDNYF FYYGTTVFNA VGM---SDSF ETSIVFGVVN FFSTCCS-LY
(PRED) P39003_Saccharo LQQLTGDNYF FYYGTTIFKA VGL---SDSF ETSIVLGIVN FASTFVG-IY
(PRED) P39004_Saccharo LQQLTGDNYF FYYGTTIFKA VGL---SDSF ETSIVLGIVN FASTFVG-IY
(PRED) P32467_Saccharo LQQLTGDNYF FYYGTTVFTA VGL---EDSF ETSIVLGIVN FASTFVG-IF
(PRED) P38695_Saccharo LQQLTGDNYF FYYGTTIFQA VGL---EDSF ETAIVLGVVN FVSTFFS-LY
(PRED) P54862_Saccharo LQQLTGDNYF FYYGTTIFKS VGL---KDSF QTSIIIGVVN FFSSFIA-VY
(PRED) P40885_Saccharo LQQLTGDNYF FYYGTTIFKS VGL---KDSF QTSIIIGVVN FFSSFIA-VY
(PRED) P39924_Saccharo FLQLTGENYF FFYGTTIFKS VGL---TDGF ETSIVLGTVN FFSTIIA-VM
(PRED) P53631_Saccharo FLQLTGENYF FFYGTTIFKS VGL---TDGF ETSIVLGTVN FFSTIIA-VM
(PRED) P54854_Saccharo FLQLTGENYF FFYGTTIFKS VGL---TDGF ETSIVLGTVN FFSTIIA-VM
(PRED) P47185_Saccharo FLQLTGENYF FFYGTTIFKS VGL---TDGF ETSIVLGTVN FFSTIIA-VM
(PRED) P10870_Saccharo FQQFSGINFI FYYGVNFFNK TGV---SNSY LVSFITYAVN VVFNVPG-LF
(PRED) P43581_Saccharo LQQLTGCNYF FYYGTTIFNA VGM---QDSF ETSIVLGAVN FASTFVA-LY
(PRED) P23585_Saccharo LQQLTGNNYF FYYGTTIFNA VGM---KDSF QTSIVLGIVN FASTFVA-LY
(PRED) O74849_Schizosa FQQLTGNNYF FYYGTQVFRG TGL---NSPF LAALILDAVN FGCTFGA-IF
(PRED) P15325_Emericel WQNGSGINAI NYYSPRVFKS IGVSGGNTSL LTTGIFGVVK AVITFVWLLY
(PRED) Q59TA4_Candida_ WQQLTGMNVM MYYIVYIFQM AGYSG-NSNL VASSIQYVIN TCVTVPA-LY
(PRED) Q59TK2_Candida_ WQQLTGMNVM MYYIVYIFQM AGYSG-NSNL VASSIQYVIN TCVTVPA-LY
(PRED) Q8L6Z8_Arabidop FQQITGQPSV LYYAPSILQT AGFSAAADAT RISILLGLLK LVMTGVS-VI
(PRED) Q94AZ2_Arabidop FQQCTGINAI MFYAPVLFST LGFGS-DASL YSAVVTGAVN VLSTLVS-IY
(PRED) Q8BFW9_Mus_musc FVQTTGQPNI LFYASTVLKS VGFQSNEAAS LASTGVGVVK VVSTIPA-TL
(PRED) Q7KWJ5_Plasmodi LQQFTGINVL VSNSNELYKE FLDS--HLIT ILSVVMTAVN FLMTFPA-IY
(PRED) O44827_Caenorha AQQLSGINVA MFYSTVIFRG AGLTG-NEPF YATIGMGAVN VIMTLIS-VW
(PRED) P11166_Homo_sap SQQLSGINAV FYYSTSIFEK AGV---QQPV YATIGSGIVN TAFTVVS-LF
(PRED) P11167_Rattus_n SQQLSGINAV FYYSTSIFEK AGV---QQPV YATIGSGIVN TAFTVVS-LF
(PRED) P17809_Mus_musc SQQLSGINAV FYYSTSIFEK AGV---QQPV YATIGSGIVN TAFTVVS-LF
(PRED) P14142_Mus_musc SQQLSGINAV FYYSTSIFES AGV---GQPA YATIGAGVVN TVFTLVS-VL
(PRED) P19357_Rattus_n SQQLSGINAV FYYSTSIFEL AGV---EQPA YATIGAGVVN TVFTLVS-VL
(PRED) P14246_Mus_musc AQQFSGINGI FYYSTSIFQT AGI---SQPV YATIGVGAIN MIFTAVS-VL
(PRED) P12336_Rattus_n AQQFSGINGI FYYSTSIFQT AGI---SQPV YATIGVGAIN MIFTAVS-VL
(PRED) P11168_Homo_sap AQQFSGINGI FYYSTSIFQT AGI---SKPV YATIGVGAVN MVFTAVS-VF
(PRED) P32037_Mus_musc SQQLSGINAV FYYSTGIFKD AGV---QEPI YATIGAGVVN TIFTVVS-LF
(PRED) Q07647_Rattus_n SQQFSGINAV FYYSTGIFQD AGV---QEPI YATIGAGVVN TIFTVVS-LF
(PRED) P43427_Rattus_n GQQLSGVNAI YYYADQIYLS AGVKS-NDVQ YVTAGTGAVN VFMTMVT-VF
(PRED) Q9JIF3_Mus_musc FQQLSGVNAI MFYANSIFEE AKF---KDSS LASVTVGIIQ VLFTAVA-AL
(PRED) Q8K4S3_Rattus_n IDSVLIDCFT AGHDNYLALP SSV---PRQA ISVSFPRAPD GFYCQERGLE
```

```
                            .........510 .........520.........530.........540.........550
(PRED) Q8VZ80_Arabidop LLD--RIGRR  PLLLTSVGGM VLSLAALGTS LTII--DQSE K---------
(PRED) G1UB10_Candida_ LVE--TWGRR  PLLMAGSVVM AVSQLIVAIV GVAA--S--- ----------
(PRED) Q5B0S0_Emericel IVE--HFGRR  ASLMVGGAWM AICFYIWASV GNSV--LDLD N---------
(PRED) P13181_Saccharo TVE--NLGHR  KCLLLGAATM MACMVIYASV GVTR--LYPH G---------
(PRED) P40886_Saccharo SVD--KLGRR  RCLLLGAATM TACMVIYASV GVTR--LYPN G---------
(PRED) P32465_Saccharo TVD--RFGRR  NCLMWGAVGM VCCYVVYASV GVTR--LWPN G---------
(PRED) P32466_Saccharo TVD--RFGRR  NCLLYGAIGM VCCYVVYASV GVTR--LWPN G---------
(PRED) P39003_Saccharo VVE--RYGRR  TCLLWGAASM TACMVVYASV GVTR--LWPN G---------
(PRED) P39004_Saccharo VVE--RYGRR  TCLLWGAASM TACMVVYASV GVTR--LWPN G---------
(PRED) P32467_Saccharo LVE--RYGRR  RCLLWGAASM TACMVVFASV GVTR--LWPN G---------
(PRED) P38695_Saccharo TVD--RFGRR  NCLLWGCVGM ICCYVVYASV GVTR--LWPN G---------
(PRED) P54862_Saccharo TIE--RFGRR  TCLLWGAASM LCCFAVFASV GVTK--LWPQ G---------
(PRED) P40885_Saccharo TIE--RFGRR  TCLLWGAASM LCCFAVFASV GVTK--LWPQ G---------
(PRED) P39924_Saccharo VVD--KIGRR  KCLLFGAAGM MACMVIFASI GVKC--LYPH G---------
(PRED) P53631_Saccharo VVD--KIGRR  KCLLFGAAGM MACMVIFASI GVKC--LYPH G---------
(PRED) P54854_Saccharo VVD--KIGRR  KCLLFGAASM MACMVIFASI GVKC--LYPH G---------
(PRED) P47185_Saccharo VVD--KIGRR  KCLLFGAASM MACMVIFASI GVKC--LYPH G---------
(PRED) P10870_Saccharo FVE--FFGRR  KVLVVGGVIM TIANFIVAIV GCSL------ ----------
(PRED) P43581_Saccharo IVD--KFGRR  KCLLWGSASM AICFVIFATV GVTR--LWPQ G---------
(PRED) P23585_Saccharo TVD--KFGRR  KCLLGGSASM AICFVIFSTV GVTS--LYPN G---------
(PRED) O74849_Schizosa VLE--YFGRR  GPLIVGGVWQ SICFFIYASV GDRA--LTRP N---------
(PRED) P15325_Emericel LID--HFGRR  NLLLVGAAGG SVCLWIVGGY IKIA--KPEN N---------
(PRED) Q59TA4_Candida_ FID--KVGRR  PLLIGGATMM MAFQFGLAGI LGQY--SIPW PDS---GNDS
(PRED) Q59TK2_Candida_ FID--KVGRR  PLLIGGATMM MAFQFGLAGI LGQY--SIPW PDS---GNDS
(PRED) Q8L6Z8_Arabidop VID--RVGRR  PLLLCGVSGM VISLFLLGSY YMFY------ ----------
(PRED) Q94AZ2_Arabidop SVD--KVGRR  VLLLEAGVQM FFSQVVIAII LGVK--V--- ----------
(PRED) Q8BFW9_Mus_musc LVD--HIGSK  TFLCIGSSVM SASLLTMGIV NLNI--NMNF ----------
(PRED) Q7KWJ5_Plasmodi IVE--KLGRK  TLLLWGCVGV LVAYLPTAIA NEIN--R--- ----------
(PRED) O44827_Caenorha LVDHPKFGRR  SLLLAGLTGM FVSTLLLVGA LTIQ--NSG- ----------
(PRED) P11166_Homo_sap VVE--RAGRR  TLHLIGLAGM AGCAILMTIA LALL--EQ-- ----------
(PRED) P11167_Rattus_n VVE--RAGRR  TLHLIGLAGM AGCAVLMTIA LALL--EQ-- ----------
(PRED) P17809_Mus_musc VVE--RAGRR  TLHLIGLAGM AGCAVLMTIA LALL--ER-- ----------
(PRED) P14142_Mus_musc LVE--RAGRR  TLHLLGLAGM CGCAILMTVA LLLL--ER-- ----------
(PRED) P19357_Rattus_n LVE--RAGRR  TLHLLGLAGM CGCAILMTVA LLLL--ER-- ----------
(PRED) P14246_Mus_musc LVE--KAGRR  TLFLTGMIGM FFCTIFMSVG LVLL--DK-- ----------
(PRED) P12336_Rattus_n LVE--KAGRR  TLFLAGMIGM FFCAVFMSLG LVLL--DK-- ----------
(PRED) P11168_Homo_sap LVE--KAGRR  SLFLIGMSGM FVCAIFMSVG LVLL--NK-- ----------
(PRED) P32037_Mus_musc LVE--RAGRR  TLHMIGLGGM AVCSVFMTIS LLLK--DD-- ----------
(PRED) Q07647_Rattus_n LVE--RAGRR  TLHMIGLGGM AVCSVFMTIS LLLK--DE-- ----------
(PRED) P43427_Rattus_n VVE--LWGRR  NLLLIGFSTC LTACIVLTVA LALQ--NT-- ----------
(PRED) Q9JIF3_Mus_musc IMD--RAGRR  LLLALSGVIM VFSMSAFGTY FKLT--QSLP SNSSHVGLVP
(PRED) Q8K4S3_Rattus_n RREGPLTLGL  DGDVLRVGSL DTSKPRASGI LKRPQTLALP DVAGGNGPET

                            .........560 .........570.........580.........590.........600
(PRED) Q8VZ80_Arabidop -------KVM  WAVVVAIATV MTYVATFSIG AGPITWVYSS EIF-PLRLRS
(PRED) G1UB10_Candida_ ---------SH AANQCLVAFS CIFIAGFAAT WGPLCWAICG ESF-ALNVRS
(PRED) Q5B0S0_Emericel -PQ----NTP  KAGAAMIVFT CFFIAGFATT WGPIVWSICS EMY-PNRSRA
(PRED) P13181_Saccharo -KSQP--SSK  GAGNCMIVFT CFYIFCYATT WAPVAWVITA ESF-PLRVKS
(PRED) P40886_Saccharo -KSEP--SSK  GAGNCTIVFT CFYIFCFSCT WGPVCYVIIS ETF-PLRVRS
(PRED) P32465_Saccharo -QDQP--SSK  GAGNCMIVFA CFYIFCFATT WAPIAYVVIS ECF-PLRVKS
(PRED) P32466_Saccharo -EGNG--SSK  GAGNCMIVFA CFYIFCFATT WAPIAYVVIS ETF-PLRVKS
(PRED) P39003_Saccharo -QDQP--SSK  GAGNCMIVFA CFYIFCFATT WAPIPYVVVS ETF-PLRVKS
(PRED) P39004_Saccharo -QDQP--SSK  GAGNCMIVFA CFYIFCFATT WAPIPYVVVS ETF-PLRVKS
(PRED) P32467_Saccharo -KKNG--SSK  GAGNCMIVFT CFYLFCFATT WAPIPFVVNS ETF-PLRVKS
(PRED) P38695_Saccharo -QDQP--SSK  GAGNCMIVFA CFYIFCFATT WAPVAYVLIS ESY-PLRVRG
(PRED) P54862_Saccharo -SSHQDITSQ  GAGNCMIVFT MFFIFSFATT WAGGCYVIVS ETF-PLRVKS
(PRED) P40885_Saccharo -SSHQDITSQ  GAGNCMIVFT MFFIFSFATT WAGGCYVIVS ETF-PLRVKS
(PRED) P39924_Saccharo -QDGP--SSK  GAGNAMIVFT CFYIFCFATT WAPVAYIVVA ESF-PSKVKS
(PRED) P53631_Saccharo -QDGP--SSK  GAGNAMIVFT CFYIFCFATT WAPVAYIVVA ESF-PSKVKS
(PRED) P54854_Saccharo -QDGP--SSK  GAGNAMIVFT CFYIFCFATT WAPVAYIVVA ESF-PSKVKS
(PRED) P47185_Saccharo -QDGP--SSK  GAGNAMIVFT CFYIFCFATT WAPVAYIVVA ESF-PSKVKS
(PRED) P10870_Saccharo -------KTV  AAAKVMIAFI CLFIAAFSAT WGGVVWVISA ELY-PLGVRS
(PRED) P43581_Saccharo -KDQP--SSQ  SAGNVMIVFT CFFIFSFAIT WAPIAYVIVA ETY-PLRVKN
(PRED) P23585_Saccharo -KDQP--SSK  AAGNVMIVFT CLFIFFFAIS WAPIAYVIVA ESY-PLRVKN
(PRED) O74849_Schizosa -GT----SNH  RAGAVMIVFS CLFIFSFAQT WAPAAYVIVG ESY-PIRYRS
(PRED) P15325_Emericel -PEGT--QLD  SGGIAAIFFF YLWTAFYTPS WNGTPWVINS EMF-DPTVRS
(PRED) Q59TA4_Candida_ VNIRIPEDNK  SASKGAIACC YLFVASFAFT WGVGIWVYCA EIWGDNRVAQ
(PRED) Q59TK2_Candida_ VNIRIPEDNK  SASKGAIACC YLFVASFAFT WGVGIWVYCA EIWGDNRVAQ
(PRED) Q8L6Z8_Arabidop ---------K  NVPAVAVAAL LLYVGCYQLS FGPIGWLMIS EIF-PLKLRG
(PRED) Q94AZ2_Arabidop -TDTSTNLSK  GFAILVVVMI CTYVAAFAWS WGPLGWLIPS ETF-PLETRS
(PRED) Q8BFW9_Mus_musc -------TNI  CRSHSLLNQS LEEFVFYATG NLSISNSSLR EHFKRITPYS
(PRED) Q7KWJ5_Plasmodi -------NSN  FVKILSIVAT FVMIISFAVS YGPVLWIYLH EMF-PSEIKD
(PRED) O44827_Caenorha -------GDK  WASYSAIGFV LLFVISFATG PGAIPWFFVS EIF-DSSARG
```

```
(PRED) P11166_Homo_sap ---------LP WMSYLSIVAI FGFVAFFEVG PGPIPWFIVA ELF-SQGPRP
(PRED) P11167_Rattus_n ---------LP WMSYLSIVAI FGFVAFFEVG PGPIPWFIVA ELF-SQGPRP
(PRED) P17809_Mus_musc ---------LP WMSYLSIVAI FGFVAFFEVG PGPIPWFIVA ELF-SQGPRP
(PRED) P14142_Mus_musc ---------VP AMSYVSIVAI FGFVAFFEIG PGPIPWFIVA ELF-SQGPRP
(PRED) P19357_Rattus_n ---------VP SMSYVSIVAI FGFVAFFEIG PGPIPWFIVA ELF-SQGPRP
(PRED) P14246_Mus_musc ---------FA WMSYVSMTAI FLFVSFFEIG PGPIPWFMVA EFF-SQGPRP
(PRED) P12336_Rattus_n ---------FT WMSYVSMTAI FLFVSFFEIG PGPIPWFMVA EFF-SQGPRP
(PRED) P11168_Homo_sap ---------FS WMSYVSMIAI FLFVSFFEIG PGPIPWFMVA EFF-SQGPRP
(PRED) P32037_Mus_musc ---------YE AMSFVCIVAI LIYVAFFEIG PGPIPWFIVA ELF-SQGPRP
(PRED) Q07647_Rattus_n ---------YE AMSFVCIVAI LVYVAFFEIG PGPIPWFIVA ELF-SQGPRP
(PRED) P43427_Rattus_n ---------IS WMPYVSIVCV IVYVIGHAVG PSPIPALFIT EIF-LQSSRP
(PRED) Q9JIF3_Mus_musc IAAEPVDVQV GLAWLAVGSM CLFIAGFAVG WGPIPWLLMS EIF-PLHVKG
(PRED) Q8K4S3_Rattus_n SRRRNVTFSQ QVANILLNGV KYESELTGSS EQSEQPLSLR RLCSTIYNMP

                       .........610 .........620.........630.........640.........650
(PRED) Q8VZ80_Arabidop QGSSMGVVVN ----RVTSGV ISISFLPMSK A--------M TTGGAFY-LF
(PRED) G1UB10_Candida_ KSISLCTASN ----WLWNFG IGY-ATPYMV DSGKGNA--D LGSKVFF-IW
(PRED) Q5B0S0_Emericel TSIGIATCAN ----WTWNFL ISF-FTPFIS G--------S IHFAYGY-VF
(PRED) P13181_Saccharo KCMALASASN ----WVWGFL IAF-FTPFIT S--------A INFYYGY-VF
(PRED) P40886_Saccharo KCMSVATAAN ----LLWGFL IGF-FTPFIT S--------A INFYYGY-VF
(PRED) P32465_Saccharo KCMSIASAAN ----WIWGFL ISF-FTPFIT G--------A INFYYGY-VF
(PRED) P32466_Saccharo KAMSIATAAN ----WLWGFL IGF-FTPFIT G--------A INFYYGY-VF
(PRED) P39003_Saccharo KAMSIATAAN ----WLWGFL IGF-FTPFIT G--------A INFYYGY-VF
(PRED) P39004_Saccharo KAMSIATAAN ----WLWGFL IGF-FTPFIT G--------A INFYYGY-VF
(PRED) P32467_Saccharo KCMAIAQACN ----WIWGFL IGF-FTPFIS G--------A IDFYYGY-VF
(PRED) P38695_Saccharo KAMSIASACN ----WIWGFL ISF-FTPFIT S--------A INFYYGY-VF
(PRED) P54862_Saccharo RGMAIATAAN ----WMWGFL ISF-FTPFIT G--------A INFYYGY-VF
(PRED) P40885_Saccharo RGMAIATAAN ----WMWGFL ISF-FTPFIT G--------A INFYYGY-VF
(PRED) P39924_Saccharo RAMSISTACN ----WLWQFL IGF-FTPFIT G--------S IHFYYGY-VF
(PRED) P53631_Saccharo RAMSISTACN ----WLWQFL IGF-FTPFIT G--------S IHFYYGY-VF
(PRED) P54854_Saccharo KAMSISTAFN ----WLWQFL IGF-FTPFIT G--------S IHFYYGY-VF
(PRED) P47185_Saccharo KAMSISTAFN ----WLWQFL IGF-FTPFIT G--------S IHFYYGY-VF
(PRED) P10870_Saccharo KCTAICAAAN ----WLVNFI CAL-ITPYIV DTGSHTS--S LGAKIFF-IW
(PRED) P43581_Saccharo RAMAIAVGAN ----WMWGFL IGF-FTPFIT R--------S IGFSYGY-VF
(PRED) P23585_Saccharo RAMAIAVGAN ----WIWGFL IGF-FTPFIT S--------A IGFSYGY-VF
(PRED) O74849_Schizosa KCAAVATASN ----WFWNFM ISF-FTPFIS N--------S IGFKYGY-VF
(PRED) P15325_Emericel LAQACAAASN ----WLWNFL ISR-FTPQMF T--------S MGYGVYF-FF
(PRED) Q59TA4_Candida_ RGNAISTSAN ----WILNFA IAM-YTPSGF K--------N ISWKTYI-IY
(PRED) Q59TK2_Candida_ RGNAISTSAN ----WILNFA IAM-YTPTGF K--------N ISWKTYI-IY
(PRED) Q8L6Z8_Arabidop RGISLAVLVN ----FGANAL VTF-AFSPLK E--------L LGAGILFCAF
(PRED) Q94AZ2_Arabidop AGQSVTVCVN ----LLFTFI IAQ-AFLSML C--------H FKFGIFI-FF
(PRED) Q8BFW9_Mus_musc KGSFMPMGNG MEPKGEMTFT SSLPNAGLSR TE-------H QGVTDTAVVP
(PRED) Q7KWJ5_Plasmodi SAASLASLVN ----WVCAII VVF-PSDIII K--------K SPSILFI-VF
(PRED) O44827_Caenorha NANSIAVMVN ----WAANLL VGL-TFLPIN N--------L MQQYSFF-IF
(PRED) P11166_Homo_sap AAIAVAGFSN ----WTSNFI VGM-CFQYVE Q--------L CGPYVFI-IF
(PRED) P11167_Rattus_n AAVAVAGFSN ----WTSNFI VGM-CFQYVE Q--------L CGPYVFI-IF
(PRED) P17809_Mus_musc AAIAVAGFSN ----WTSNFI VGM-CFQYVE Q--------L CGPYVFI-IF
(PRED) P14142_Mus_musc AAMAVAGFSN ----WTCNFI VGM-GFQYVA D--------A MGPYVFL-LF
(PRED) P19357_Rattus_n AAMAVAGFSN ----WTCNFI VGM-GFQYVA D--------A MGPYVFL-LF
(PRED) P14246_Mus_musc TALALAAFSN ----WVCNFV IAL-CFQYIA D--------F LGPYVFF-LF
(PRED) P12336_Rattus_n TALALAAFSN ----WVCNFI IAL-CFQYIA D--------F LGPYVFF-LF
(PRED) P11168_Homo_sap AALAIAAFSN ----WTCNFI VAL-CFQYIA D--------F CGPYVFF-LF
(PRED) P32037_Mus_musc AAIAVAGCCN ----WTSNFL VGM-LFPSAA A--------Y LGAYVFI-IF
(PRED) Q07647_Rattus_n AAMAVAGCSN ----WTSNFL VGM-FFPSAA A--------Y LGAYVFI-IF
(PRED) P43427_Rattus_n SAYMIGGSVH ----WLSNFI VGL-IFPFIQ V--------G LGPYSFI-IF
(PRED) Q9JIF3_Mus_musc VATGICVLTN ----WFMAFL VTK-EFSSVM E--------M LRPYGAFWLT
(PRED) Q8K4S3_Rattus_n RPVRNLCVNH F-LGWLSFEG MLLFYTDFMG EVVFQGDPKA PHASEAYQKY

                       .........660 .........670.........680.........690.........700
(PRED) Q8VZ80_Arabidop GGIA--TVAW VFFYTFLPET QGRMLEDMDE LFSGF--RWR DSKSKPKGNP
(PRED) G1UB10_Candida_ GGCN--VIGG LFAYFMVYET KSLTLEQVDE LYLKVDHAWQ SKGFVPSVHA
(PRED) Q5B0S0_Emericel ASCC--VVGV LIVFFFVNET QGRTLEEVDT MYVLHVVPWK SASWVPDESI
(PRED) P13181_Saccharo MGCL--VAMF FYVFFFVPET KGLSLEEIQE LWEEGVLPWK SEGWIPSSRR
(PRED) P40886_Saccharo MGCL--AFSY FYVFFFVPET KGLTLEEVDE MWMDGVLPWK SESWVPASRR
(PRED) P32465_Saccharo MGCM--VFAY FYVFFFVPET KGLSLEEVND MYAEGVLPWK SASWVPVSKR
(PRED) P32466_Saccharo MGCM--VFAY FYVFFFVPET KGLTLEEVND MYAEGVLPWK SASWVPTSQR
(PRED) P39003_Saccharo MGCL--VFMF FYVLLVVPET KGLTLEEVNT MWEEGVLPWK SASWVPPSRR
(PRED) P39004_Saccharo MGCL--VFMF FYVLLVVPET KGLTLEEVNT MWEEGVLPWK SASWVPPSRR
(PRED) P32467_Saccharo MGCL--VFSY FYVFFFVPET KGLTLEEVNT LWEEGVLPWK SPSWVPPNKR
(PRED) P38695_Saccharo MGCM--VFAY FYVFFFVPET KGLTLEEVNE MYEENVLPWK STKWIPPSRR
(PRED) P54862_Saccharo LGCL--VFAY FYVFFFVPET KGLTLEEVNT MWLEGVPAWK SASWVPPERR
(PRED) P40885_Saccharo LGCL--VFAY FYVFFFVPET KGLTLEEVNT MWLEGVPAWK SASWVPPERR
(PRED) P39924_Saccharo VGCL--VAMF LYVFFFLPET IGLSLEEIQL LYEEGIKPWK SASWVPPSRR
(PRED) P53631_Saccharo VGCL--VAMF LYVFFFLPET IGLSLEEIQL LYEEGIKPWK SASWVPPSRR
(PRED) P54854_Saccharo VGCL--VAMF LYVFFFLPET IGLSLEEIQL LYEEGIKPWK SASWVPPSRR
```

```
(PRED) P47185_Saccharo VGCL--VAMF LYVFFFLPET IGLSLEETQL LYEEGIKPWK SASWVPPSRR
(PRED) P10870_Saccharo GSLN--AMGV IVVYLTVYET KGLTLEEIDE LYIKSSTGVV SPKFNKDIRE
(PRED) P43581_Saccharo MGCL--IFSY FYVFFFVCET KGLTLEEVNE MYEERIKPWK SGGWIPSSRR
(PRED) P23585_Saccharo MGCL--VFSF FYVFFFVCET KGLTLEEVNE MYVEGVKPWK SGSWISKEKR
(PRED) O74849_Schizosa AACN--LCAA IIIFLFAKET KGLTLEEINQ LYLSNIKPWN TGAYQRDRED
(PRED) P15325_Emericel ASLM--ILSI VFVFFLIPET KGVPLESMET LFDKKP-VWH AHSQLIRELR
(PRED) Q59TA4_Candida_ GVFC--FAMA THVYFGFPET KGKRLEEIGQ MWEERVPAWR SRSWQPTVPI
(PRED) Q59TK2_Candida_ GVFC--FAMA THVYFGFPET KGKRLEEIGQ MWEERVPAWR SRSWQPTVPI
(PRED) Q8L6Z8_Arabidop GVIC--VVSL FFIYYIVPET KGLTLEEIEA KCL------- ----------
(PRED) Q94AZ2_Arabidop SAWV--LIMS VFVMFLLPET KNIPIEEMTE RVWKK--HWF WARFMDDHND
(PRED) Q8BFW9_Mus_musc AAYK--WLSL ASLLVYVA-- -AFSIGLGPM PW-------- ----------
(PRED) Q7KWJ5_Plasmodi SVMS--ILTF FFIFFFIKET KGGEIGTSPY ITMEERQKHM TKSVV-----
(PRED) O44827_Caenorha SGFL--AFFI FYTWKFVPET KGKSIEQIQA EFEKRK---- ----------
(PRED) P11166_Homo_sap TVLL--VLFF IFTYFKVPET KGRTFDEIAS GFRQG-GASQ -SDKTPEELF
(PRED) P11167_Rattus_n TVLL--VLFF IFTYFKVPET KGRTFDEIAS GFRQG-GASQ -SDKTPEELF
(PRED) P17809_Mus_musc TVLL--VLFF IFTYFKVPET KGRTFDEIAS GFRQG-GASQ -SDKTPEELF
(PRED) P14142_Mus_musc AVLL--LGFF IFTFLKVPET RGRTFDQISA AFRRTPSLLE -QEVKPSTEL
(PRED) P19357_Rattus_n AVLL--LGFF IFTFLRVPET RGRTFDQISA TFRRTPSLLE -QEVKPSTEL
(PRED) P14246_Mus_musc AGVV--LVFT LFTFFKVPET KGKSFEEIAA EFRKK-SGSA -PPRKAAVQM
(PRED) P12336_Rattus_n AGVV--LVFT LFTFFKVPET KGKSFDEIAA EFRKK-SGSA -PPRKATVQM
(PRED) P11168_Homo_sap AGVL--LAFT LFTFFKVPET KGKSFEEIAA EFQKK-SGSA -HRPKAAVEM
(PRED) P32037_Mus_musc AAFL--IFFL IFTFFKVPET KGRTFEDIAR AFEGQ--AHS -GKGPAGVEL
(PRED) Q07647_Rattus_n AAFL--VFFL IFTSFKVPET KGRTFEDITR AFEGQ--AHS -GKGSAGVEL
(PRED) P43427_Rattus_n AIIC--LLTT IYIFMVVPET KGRTFVEINQ IFAKKNKVSD VYPEKEEKEL
(PRED) Q9JIF3_Mus_musc AAFC--ALSV LFTLTVVPET KGRTLEQVTA HFEGR----- ----------
(PRED) Q8K4S3_Rattus_n NSGVTMGCWG MCIYAFSAAF YSAILEKLEE CLSVRTLYFI AYLLFGLGTG

                       .........710.........720.........730.........740.........750
(PRED) Q8VZ80_Arabidop EKTV--PNPE VEIGSNKQWK EGDTQSS--- ---------- ----------
(PRED) G1UB10_Candida_ FRDD--GDIE HISSDGKAEM VEVDENSV-- ---------- ----------
(PRED) Q5B0S0_Emericel VRD---LHPG SDANKTEGLG QAEHGEESRP EPVEIRE--- ----------
(PRED) P13181_Saccharo GNN---YDLE DLQHDDKPWY KAMLE----- ---------- ----------
(PRED) P40886_Saccharo DGD---YDNE KLQHDEKPFY KRMF------ ---------- ----------
(PRED) P32465_Saccharo GAD---YNAD DLMHDDQPFY KSLFSRK--- ---------- ----------
(PRED) P32466_Saccharo GAN---YDAD ALMHDDQPFY KKMFGKK--- ---------- ----------
(PRED) P39003_Saccharo GAN---YDAE EMAHDDKPLY KRMFSTK--- ---------- ----------
(PRED) P39004_Saccharo GAN---YDAE EMTHDDKPLY KRMFSTK--- ---------- ----------
(PRED) P32467_Saccharo GTD---YNAD DLMHDDQPFY KKMFGKK--- ---------- ----------
(PRED) P38695_Saccharo TTD---YDLD ATRNDPRPFY KRMFTKEK-- ---------- ----------
(PRED) P54862_Saccharo TAD---YDAD AIDHDNRPIY KRFFSS---- ---------- ----------
(PRED) P40885_Saccharo TAD---YDAD AIDHDDRPIY KRFFSS---- ---------- ----------
(PRED) P39924_Saccharo GIS---SEES KTEKKDWKKF LKFSKNSD-- ---------- ----------
(PRED) P53631_Saccharo GIP---SEES KTEKKDWKKF LKFSKGSD-- ---------- ----------
(PRED) P54854_Saccharo GAS---SRET EAKKKSWKEV LKFPKSFN-- ---------- ----------
(PRED) P47185_Saccharo GAS---SRET EAKKKSWKEV LKFPKSFN-- ---------- ----------
(PRED) P10870_Saccharo RALKFQYDPL QRLEDGKNTF VAKRNNFDDE TPRNDFRNTI SGEIDHSPNQ
(PRED) P43581_Saccharo TPQP-TSSTP LVIVDSK--- ---------- ---------- ----------
(PRED) P23585_Saccharo VSEE------ ---------- ---------- ---------- ----------
(PRED) O74849_Schizosa IKQ---SDSE KERGPTSKLH EYVEHAPNSY ASTHSTESEN YPQQVTNPVG
(PRED) P15325_Emericel ENEEAFRADM GASGKGGVTK EYVEEA---- ---------- ----------
(PRED) Q59TA4_Candida_ ASDAELARKM EVEHEEDKLM NEDSNSESRE NQA------- ----------
(PRED) Q59TK2_Candida_ ASDAELARKM EVEHEEDKLM NEDSNSESRE NQA------- ----------
(PRED) Q8L6Z8_Arabidop ---------- ---------- ---------- ---------- ----------
(PRED) Q94AZ2_Arabidop HEF---VNGE KSNGKSNGFD PSTRL----- ---------- ----------
(PRED) Q8BFW9_Mus_musc ---------- ---------- ---------- ---------- ----------
(PRED) Q7KWJ5_Plasmodi ---------- ---------- ---------- ---------- ----------
(PRED) O44827_Caenorha ---------- ---------- ---------- ---------- ----------
(PRED) P11166_Homo_sap HPLGADSQV- ---------- ---------- ---------- ----------
(PRED) P11167_Rattus_n HPLGADSQV- ---------- ---------- ---------- ----------
(PRED) P17809_Mus_musc HPLGADSQV- ---------- ---------- ---------- ----------
(PRED) P14142_Mus_musc EYLGPDEND- ---------- ---------- ---------- ----------
(PRED) P19357_Rattus_n EYLGPDEND- ---------- ---------- ---------- ----------
(PRED) P14246_Mus_musc EFLASSESV- ---------- ---------- ---------- ----------
(PRED) P12336_Rattus_n EFLGSSETV- ---------- ---------- ---------- ----------
(PRED) P11168_Homo_sap KFLGATETV- ---------- ---------- ---------- ----------
(PRED) P32037_Mus_musc NSMQPVKETP GNA------- ---------- ---------- ----------
(PRED) Q07647_Rattus_n NSMQPVKETP GNA------- ---------- ---------- ----------
(PRED) P43427_Rattus_n NDLPPATREQ ---------- ---------- ---------- ----------
(PRED) Q9JIF3_Mus_musc ---------- ---------- ---------- ---------- ----------
(PRED) Q8K4S3_Rattus_n LATLS----- ---------- ---------- ---------- ----------

                       .........760.........770.........780.........790.........800
(PRED) Q8VZ80_Arabidop ---------- ---------- ---------- ---------- ----------
(PRED) G1UB10_Candida_ ---------- ---------- ---------- ---------- ----------
(PRED) Q5B0S0_Emericel ---------- ---------- ---------- ---------- ----------
```

```
(PRED) P13181_Saccharo ---------- ---------- ---------- ---------- ----------
(PRED) P40886_Saccharo ---------- ---------- ---------- ---------- ----------
(PRED) P32465_Saccharo ---------- ---------- ---------- ---------- ----------
(PRED) P32466_Saccharo ---------- ---------- ---------- ---------- ----------
(PRED) P39003_Saccharo ---------- ---------- ---------- ---------- ----------
(PRED) P39004_Saccharo ---------- ---------- ---------- ---------- ----------
(PRED) P32467_Saccharo ---------- ---------- ---------- ---------- ----------
(PRED) P38695_Saccharo ---------- ---------- ---------- ---------- ----------
(PRED) P54862_Saccharo ---------- ---------- ---------- ---------- ----------
(PRED) P40885_Saccharo ---------- ---------- ---------- ---------- ----------
(PRED) P39924_Saccharo ---------- ---------- ---------- ---------- ----------
(PRED) P53631_Saccharo ---------- ---------- ---------- ---------- ----------
(PRED) P54854_Saccharo ---------- ---------- ---------- ---------- ----------
(PRED) P47185_Saccharo ---------- ---------- ---------- ---------- ----------
(PRED) P10870_Saccharo KEVHSIPERV DIPTSTEILE SPNKSSGMTV PVSPSLQDVP IPQTTEPAEI
(PRED) P43581_Saccharo ---------- ---------- ---------- ---------- ----------
(PRED) P23585_Saccharo ---------- ---------- ---------- ---------- ----------
(PRED) O74849_Schizosa L--------- ---------- ---------- ---------- ----------
(PRED) P15325_Emericel ---------- ---------- ---------- ---------- ----------
(PRED) Q59TA4_Candida_ ---------- ---------- ---------- ---------- ----------
(PRED) Q59TK2_Candida_ ---------- ---------- ---------- ---------- ----------
(PRED) Q8L6Z8_Arabidop ---------- ---------- ---------- ---------- ----------
(PRED) Q94AZ2_Arabidop ---------- ---------- ---------- ---------- ----------
(PRED) Q8BFW9_Mus_musc ---------- ---------- ---------- ---------- ----------
(PRED) Q7KWJ5_Plasmodi ---------- ---------- ---------- ---------- ----------
(PRED) O44827_Caenorha ---------- ---------- ---------- ---------- ----------
(PRED) P11166_Homo_sap ---------- ---------- ---------- ---------- ----------
(PRED) P11167_Rattus_n ---------- ---------- ---------- ---------- ----------
(PRED) P17809_Mus_musc ---------- ---------- ---------- ---------- ----------
(PRED) P14142_Mus_musc ---------- ---------- ---------- ---------- ----------
(PRED) P19357_Rattus_n ---------- ---------- ---------- ---------- ----------
(PRED) P14246_Mus_musc ---------- ---------- ---------- ---------- ----------
(PRED) P12336_Rattus_n ---------- ---------- ---------- ---------- ----------
(PRED) P11168_Homo_sap ---------- ---------- ---------- ---------- ----------
(PRED) P32037_Mus_musc ---------- ---------- ---------- ---------- ----------
(PRED) Q07647_Rattus_n ---------- ---------- ---------- ---------- ----------
(PRED) P43427_Rattus_n ---------- ---------- ---------- ---------- ----------
(PRED) Q9JIF3_Mus_musc ---------- ---------- ---------- ---------- ----------
(PRED) Q8K4S3_Rattus_n ---------- ---------- ---------- ---------- ----------

                       .........810 .........820.........830.........840.........850
(PRED) Q8VZ80_Arabidop ---------- ---------- ---------- ---------- ----------
(PRED) G1UB10_Candida_ ---------- ---------- ---------- ---------- ----------
(PRED) Q5B0S0_Emericel ---------- ---------- ---------- ---------- ----------
(PRED) P13181_Saccharo ---------- ---------- ---------- ---------- ----------
(PRED) P40886_Saccharo ---------- ---------- ---------- ---------- ----------
(PRED) P32465_Saccharo ---------- ---------- ---------- ---------- ----------
(PRED) P32466_Saccharo ---------- ---------- ---------- ---------- ----------
(PRED) P39003_Saccharo ---------- ---------- ---------- ---------- ----------
(PRED) P39004_Saccharo ---------- ---------- ---------- ---------- ----------
(PRED) P32467_Saccharo ---------- ---------- ---------- ---------- ----------
(PRED) P38695_Saccharo ---------- ---------- ---------- ---------- ----------
(PRED) P54862_Saccharo ---------- ---------- ---------- ---------- ----------
(PRED) P40885_Saccharo ---------- ---------- ---------- ---------- ----------
(PRED) P39924_Saccharo ---------- ---------- ---------- ---------- ----------
(PRED) P53631_Saccharo ---------- ---------- ---------- ---------- ----------
(PRED) P54854_Saccharo ---------- ---------- ---------- ---------- ----------
(PRED) P47185_Saccharo ---------- ---------- ---------- ---------- ----------
(PRED) P10870_Saccharo RTKYVDLGNG LGLNTYNRGP PSLSSDSSED YTEDEIGGPS SQGDQSNRST
(PRED) P43581_Saccharo ---------- ---------- ---------- ---------- ----------
(PRED) P23585_Saccharo ---------- ---------- ---------- ---------- ----------
(PRED) O74849_Schizosa ---------- ---------- ---------- ---------- ----------
(PRED) P15325_Emericel ---------- ---------- ---------- ---------- ----------
(PRED) Q59TA4_Candida_ ---------- ---------- ---------- ---------- ----------
(PRED) Q59TK2_Candida_ ---------- ---------- ---------- ---------- ----------
(PRED) Q8L6Z8_Arabidop ---------- ---------- ---------- ---------- ----------
(PRED) Q94AZ2_Arabidop ---------- ---------- ---------- ---------- ----------
(PRED) Q8BFW9_Mus_musc ---LVL---- --SEIFPGGI RGRA------ ---------M ALTSSMNWGV
(PRED) Q7KWJ5_Plasmodi ---------- ---------- ---------- ---------- ----------
(PRED) O44827_Caenorha ---------- ---------- ---------- ---------- ----------
(PRED) P11166_Homo_sap ---------- ---------- ---------- ---------- ----------
(PRED) P11167_Rattus_n ---------- ---------- ---------- ---------- ----------
(PRED) P17809_Mus_musc ---------- ---------- ---------- ---------- ----------
(PRED) P14142_Mus_musc ---------- ---------- ---------- ---------- ----------
(PRED) P19357_Rattus_n ---------- ---------- ---------- ---------- ----------
```

```
(PRED) P14246_Mus_musc ---------- ---------- ---------- ---------- ----------
(PRED) P12336_Rattus_n ---------- ---------- ---------- ---------- ----------
(PRED) P11168_Homo_sap ---------- ---------- ---------- ---------- ----------
(PRED) P32037_Mus_musc ---------- ---------- ---------- ---------- ----------
(PRED) Q07647_Rattus_n ---------- ---------- ---------- ---------- ----------
(PRED) P43427_Rattus_n ---------- ---------- ---------- ---------- ----------
(PRED) Q9JIF3_Mus_musc ---------- ---------- ---------- ---------- ----------
(PRED) Q8K4S3_Rattus_n ---------- --RNLYVVLS LCTH------ ---------Y GILFSTLCTL

                       .........860 .........870.........880.........890.........900
(PRED) Q8VZ80_Arabidop ---------- ---------- ---------- ---------- ----------
(PRED) G1UB10_Candida_ ---------- ---------- ---------- ---------- ----------
(PRED) Q5B0S0_Emericel ---------- ---------- ---------- ---------- ----------
(PRED) P13181_Saccharo ---------- ---------- ---------- ---------- ----------
(PRED) P40886_Saccharo ---------- ---------- ---------- ---------- ----------
(PRED) P32465_Saccharo ---------- ---------- ---------- ---------- ----------
(PRED) P32466_Saccharo ---------- ---------- ---------- ---------- ----------
(PRED) P39003_Saccharo ---------- ---------- ---------- ---------- ----------
(PRED) P39004_Saccharo ---------- ---------- ---------- ---------- ----------
(PRED) P32467_Saccharo ---------- ---------- ---------- ---------- ----------
(PRED) P38695_Saccharo ---------- ---------- ---------- ---------- ----------
(PRED) P54862_Saccharo ---------- ---------- ---------- ---------- ----------
(PRED) P40885_Saccharo ---------- ---------- ---------- ---------- ----------
(PRED) P39924_Saccharo ---------- ---------- ---------- ---------- ----------
(PRED) P53631_Saccharo ---------- ---------- ---------- ---------- ----------
(PRED) P54854_Saccharo ---------- ---------- ---------- ---------- ----------
(PRED) P47185_Saccharo ---------- ---------- ---------- ---------- ----------
(PRED) P10870_Saccharo MNDINDYMAR LIHSTSTASN TTDKFSGNQS TLRYHTASSH SDTTEEDSNL
(PRED) P43581_Saccharo ---------- ---------- ---------- ---------- ----------
(PRED) P23585_Saccharo ---------- ---------- ---------- ---------- ----------
(PRED) O74849_Schizosa ---------- ---------- ---------- ---------- ----------
(PRED) P15325_Emericel ---------- ---------- ---------- ---------- ----------
(PRED) Q59TA4_Candida_ ---------- ---------- ---------- ---------- ----------
(PRED) Q59TK2_Candida_ ---------- ---------- ---------- ---------- ----------
(PRED) Q8L6Z8_Arabidop ---------- ---------- ---------- ---------- ----------
(PRED) Q94AZ2_Arabidop ---------- ---------- ---------- ---------- ----------
(PRED) Q8BFW9_Mus_musc NLLISLTFLT VTDL------ ---------- ---------- ----------
(PRED) Q7KWJ5_Plasmodi ---------- ---------- ---------- ---------- ----------
(PRED) O44827_Caenorha ---------- ---------- ---------- ---------- ----------
(PRED) P11166_Homo_sap ---------- ---------- ---------- ---------- ----------
(PRED) P11167_Rattus_n ---------- ---------- ---------- ---------- ----------
(PRED) P17809_Mus_musc ---------- ---------- ---------- ---------- ----------
(PRED) P14142_Mus_musc ---------- ---------- ---------- ---------- ----------
(PRED) P19357_Rattus_n ---------- ---------- ---------- ---------- ----------
(PRED) P14246_Mus_musc ---------- ---------- ---------- ---------- ----------
(PRED) P12336_Rattus_n ---------- ---------- ---------- ---------- ----------
(PRED) P11168_Homo_sap ---------- ---------- ---------- ---------- ----------
(PRED) P32037_Mus_musc ---------- ---------- ---------- ---------- ----------
(PRED) Q07647_Rattus_n ---------- ---------- ---------- ---------- ----------
(PRED) P43427_Rattus_n ---------- ---------- ---------- ---------- ----------
(PRED) Q9JIF3_Mus_musc ---------- ---------- ---------- ---------- ----------
(PRED) Q8K4S3_Rattus_n PYSLLCDYYQ SKKFAGSSA- ---------- ---------- ----------

                       .........910 .........920.........930.........940.........950
(PRED) Q8VZ80_Arabidop ---------- ---------- ---------- ---------- ----------
(PRED) G1UB10_Candida_ ---------- ---------- ---------- ---------- ----------
(PRED) Q5B0S0_Emericel ---------- ---------- ---------- ---------- ----------
(PRED) P13181_Saccharo ---------- ---------- ---------- ---------- ----------
(PRED) P40886_Saccharo ---------- ---------- ---------- ---------- ----------
(PRED) P32465_Saccharo ---------- ---------- ---------- ---------- ----------
(PRED) P32466_Saccharo ---------- ---------- ---------- ---------- ----------
(PRED) P39003_Saccharo ---------- ---------- ---------- ---------- ----------
(PRED) P39004_Saccharo ---------- ---------- ---------- ---------- ----------
(PRED) P32467_Saccharo ---------- ---------- ---------- ---------- ----------
(PRED) P38695_Saccharo ---------- ---------- ---------- ---------- ----------
(PRED) P54862_Saccharo ---------- ---------- ---------- ---------- ----------
(PRED) P40885_Saccharo ---------- ---------- ---------- ---------- ----------
(PRED) P39924_Saccharo ---------- ---------- ---------- ---------- ----------
(PRED) P53631_Saccharo ---------- ---------- ---------- ---------- ----------
(PRED) P54854_Saccharo ---------- ---------- ---------- ---------- ----------
(PRED) P47185_Saccharo ---------- ---------- ---------- ---------- ----------
(PRED) P10870_Saccharo MDLGNGLALN AYNRGPPSIL MNSSDEEANG GETSDNLNTA QDLAGMKERM
(PRED) P43581_Saccharo ---------- ---------- ---------- ---------- ----------
(PRED) P23585_Saccharo ---------- ---------- ---------- ---------- ----------
(PRED) O74849_Schizosa ---------- ---------- ---------- ---------- ----------
```

```
(PRED) P15325_Emericel ---------- ---------- ---------- ---------- ----------
(PRED) Q59TA4_Candida_ ---------- ---------- ---------- ---------- ----------
(PRED) Q59TK2_Candida_ ---------- ---------- ---------- ---------- ----------
(PRED) Q8L6Z8_Arabidop ---------- ---------- ---------- ---------- ----------
(PRED) Q94AZ2_Arabidop ---------- ---------- ---------- ---------- ----------
(PRED) Q8BFW9_Mus_musc IGLSWVCFIY TIMSLASLAF VVLFIPETKG ---------- ---CSLEQIS
(PRED) Q7KWJ5_Plasmodi ---------- ---------- ---------- ---------- ----------
(PRED) O44827_Caenorha ---------- ---------- ---------- ---------- ----------
(PRED) P11166_Homo_sap ---------- ---------- ---------- ---------- ----------
(PRED) P11167_Rattus_n ---------- ---------- ---------- ---------- ----------
(PRED) P17809_Mus_musc ---------- ---------- ---------- ---------- ----------
(PRED) P14142_Mus_musc ---------- ---------- ---------- ---------- ----------
(PRED) P19357_Rattus_n ---------- ---------- ---------- ---------- ----------
(PRED) P14246_Mus_musc ---------- ---------- ---------- ---------- ----------
(PRED) P12336_Rattus_n ---------- ---------- ---------- ---------- ----------
(PRED) P11168_Homo_sap ---------- ---------- ---------- ---------- ----------
(PRED) P32037_Mus_musc ---------- ---------- ---------- ---------- ----------
(PRED) Q07647_Rattus_n ---------- ---------- ---------- ---------- ----------
(PRED) P43427_Rattus_n ---------- ---------- ---------- ---------- ----------
(PRED) Q9JIF3_Mus_musc ---------- ---------- ---------- ---------- ----------
(PRED) Q8K4S3_Rattus_n DGTRRGMGVD ISLLSCQYFL AQILVS---- ---------- ---LVLGPLT

                       .........960 .........970.........980.........990.........1000
(PRED) Q8VZ80_Arabidop ---------- ---------- ---------- ---------- ----------
(PRED) G1UB10_Candida_ ---------- ---------- ---------- ---------- ----------
(PRED) Q5B0S0_Emericel ---------- ---------- ---------- ---------- ----------
(PRED) P13181_Saccharo ---------- ---------- ---------- ---------- ----------
(PRED) P40886_Saccharo ---------- ---------- ---------- ---------- ----------
(PRED) P32465_Saccharo ---------- ---------- ---------- ---------- ----------
(PRED) P32466_Saccharo ---------- ---------- ---------- ---------- ----------
(PRED) P39003_Saccharo ---------- ---------- ---------- ---------- ----------
(PRED) P39004_Saccharo ---------- ---------- ---------- ---------- ----------
(PRED) P32467_Saccharo ---------- ---------- ---------- ---------- ----------
(PRED) P38695_Saccharo ---------- ---------- ---------- ---------- ----------
(PRED) P54862_Saccharo ---------- ---------- ---------- ---------- ----------
(PRED) P40885_Saccharo ---------- ---------- ---------- ---------- ----------
(PRED) P39924_Saccharo ---------- ---------- ---------- ---------- ----------
(PRED) P53631_Saccharo ---------- ---------- ---------- ---------- ----------
(PRED) P54854_Saccharo ---------- ---------- ---------- ---------- ----------
(PRED) P47185_Saccharo ---------- ---------- ---------- ---------- ----------
(PRED) P10870_Saccharo AQFAQSYIDK RGGLEPETQS NILSTSLSVM ADTNEHNNEI LHSSEENATN
(PRED) P43581_Saccharo ---------- ---------- ---------- ---------- ----------
(PRED) P23585_Saccharo ---------- ---------- ---------- ---------- ----------
(PRED) O74849_Schizosa ---------- ---------- ---------- ---------- ----------
(PRED) P15325_Emericel ---------- ---------- ---------- ---------- ----------
(PRED) Q59TA4_Candida_ ---------- ---------- ---------- ---------- ----------
(PRED) Q59TK2_Candida_ ---------- ---------- ---------- ---------- ----------
(PRED) Q8L6Z8_Arabidop ---------- ---------- ---------- ---------- ----------
(PRED) Q94AZ2_Arabidop ---------- ---------- ---------- ---------- ----------
(PRED) Q8BFW9_Mus_musc VELAKANYVK NNICFMSHHQ EELVPTQLQK RKPQEQLPEC NHLCGRGQSQ
(PRED) Q7KWJ5_Plasmodi ---------- ---------- ---------- ---------- ----------
(PRED) O44827_Caenorha ---------- ---------- ---------- ---------- ----------
(PRED) P11166_Homo_sap ---------- ---------- ---------- ---------- ----------
(PRED) P11167_Rattus_n ---------- ---------- ---------- ---------- ----------
(PRED) P17809_Mus_musc ---------- ---------- ---------- ---------- ----------
(PRED) P14142_Mus_musc ---------- ---------- ---------- ---------- ----------
(PRED) P19357_Rattus_n ---------- ---------- ---------- ---------- ----------
(PRED) P14246_Mus_musc ---------- ---------- ---------- ---------- ----------
(PRED) P12336_Rattus_n ---------- ---------- ---------- ---------- ----------
(PRED) P11168_Homo_sap ---------- ---------- ---------- ---------- ----------
(PRED) P32037_Mus_musc ---------- ---------- ---------- ---------- ----------
(PRED) Q07647_Rattus_n ---------- ---------- ---------- ---------- ----------
(PRED) P43427_Rattus_n ---------- ---------- ---------- ---------- ----------
(PRED) Q9JIF3_Mus_musc ---------- ---------- ---------- ---------- ----------
(PRED) Q8K4S3_Rattus_n SAVGSANGVM YFASLVSFLG CLYSSLCVTY EIPSADAADE ERQPLLLNV-

                       .........1010
(PRED) Q8VZ80_Arabidop ----------
(PRED) G1UB10_Candida_ ----------
(PRED) Q5B0S0_Emericel ----------
(PRED) P13181_Saccharo ----------
(PRED) P40886_Saccharo ----------
(PRED) P32465_Saccharo ----------
(PRED) P32466_Saccharo ----------
(PRED) P39003_Saccharo ----------
```

```
(PRED) P39004_Saccharo ----------
(PRED) P32467_Saccharo ----------
(PRED) P38695_Saccharo ----------
(PRED) P54862_Saccharo ----------
(PRED) P40885_Saccharo ----------
(PRED) P39924_Saccharo ----------
(PRED) P53631_Saccharo ----------
(PRED) P54854_Saccharo ----------
(PRED) P47185_Saccharo ----------
(PRED) P10870_Saccharo QPVNENNDLK
(PRED) P43581_Saccharo ----------
(PRED) P23585_Saccharo ----------
(PRED) O74849_Schizosa ----------
(PRED) P15325_Emericel ----------
(PRED) Q59TA4_Candida_ ----------
(PRED) Q59TK2_Candida_ ----------
(PRED) Q8L6Z8_Arabidop ----------
(PRED) Q94AZ2_Arabidop ----------
(PRED) Q8BFW9_Mus_musc RPSPDT----
(PRED) Q7KWJ5_Plasmodi ----------
(PRED) O44827_Caenorha ----------
(PRED) P11166_Homo_sap ----------
(PRED) P11167_Rattus_n ----------
(PRED) P17809_Mus_musc ----------
(PRED) P14142_Mus_musc ----------
(PRED) P19357_Rattus_n ----------
(PRED) P14246_Mus_musc ----------
(PRED) P12336_Rattus_n ----------
(PRED) P11168_Homo_sap ----------
(PRED) P32037_Mus_musc ----------
(PRED) Q07647_Rattus_n ----------
(PRED) P43427_Rattus_n ----------
(PRED) Q9JIF3_Mus_musc ----------
(PRED) Q8K4S3_Rattus_n ----------
```
