# Supplementary material for: Aspergillus niger membrane-associated proteome analysis for the identification of glucose transporters
Source: Biotechnol Biofuels. 2015 Sep 17;8:150. doi: 10.1186/s13068-015-0317-9 (PMC4574540; doi:10.1186/s13068-015-0317-9)

ROC curves comparing the performance of Blast vs. HMMs to identify glucose transporters

True and false positive rates were calculated for each approach using the same core dataset (described in the Methods section) plus the verified glucose transporters (obtained from the UniProt database, see Methods section for details). The two discussed thresholds of the final HMM_{gluT}, d_{min} and MCC_{max}, are marked on the HMM ROC curve (white square and black circle, respectively).

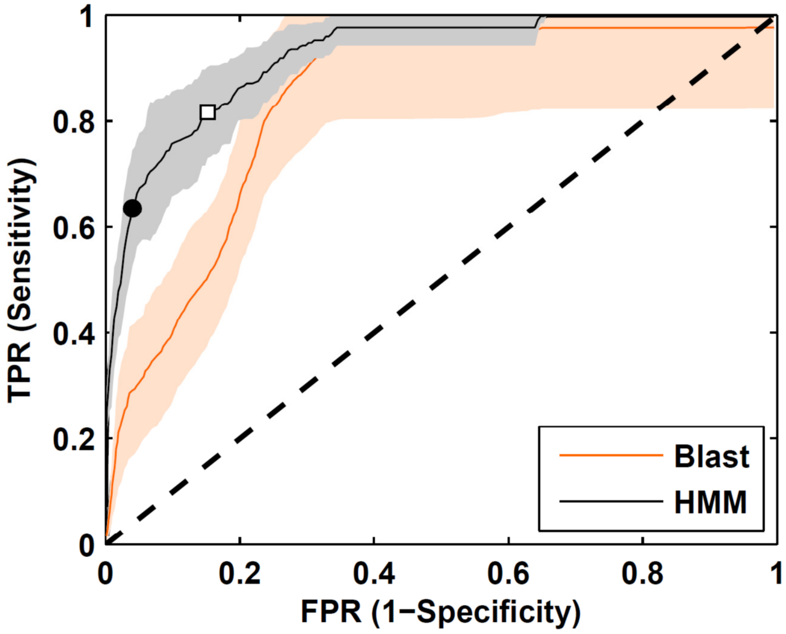

Supplement: Supplementary file 2 — Additional file 2. ROC curves comparing the performance of Blast vs. HMMs to identify glucose transporters. [file 13068_2015_317_MOESM2_ESM.pdf]
