# Supplementary material for: Aspergillus niger membrane-associated proteome analysis for the identification of glucose transporters
Source: Biotechnol Biofuels. 2015 Sep 17;8:150. doi: 10.1186/s13068-015-0317-9 (PMC4574540; doi:10.1186/s13068-015-0317-9)
Supplement: Supplementary file 3 — Additional file 3. Number of proteins identified in the 3 different conditions studied in the A. niger membrane-associated proteome analysis. [file 13068_2015_317_MOESM3_ESM.pdf]

Number of proteins identified in the 3 different conditions

	N_{tot}	N_{unique}	N_{tmHMM}	N_{tmHMM}/N_{tot} [%]	Q_{tmHMM}/Q_{tot} [%]
sorbitol	651	72	187	28.73	15.28
sorbitol + 1mM glucose	543	34	155	28.55	17.50
sorbitol + 60mM glucose	692	106	193	27.89	15.45

N_{tot} = total number of proteins in the sample
N_{unique} = number of proteins found only in this condition
N_{tmHMM} = number of proteins in the sample containing at least one transmembrane helix domain
Q = averaged protein abundance [a.u]
