# Supplementary material for: Aspergillus niger membrane-associated proteome analysis for the identification of glucose transporters
Source: Biotechnol Biofuels. 2015 Sep 17;8:150. doi: 10.1186/s13068-015-0317-9 (PMC4574540; doi:10.1186/s13068-015-0317-9)
Supplement: Supplementary file 4 — Additional file 4. Relative abundances of proteins from the A. niger membrane-associated proteome with at least one tmHMM domain. [file 13068_2015_317_MOESM4_ESM.pdf]

protID	TmD	sorbitol rel. ab	±	sd [%]	sorbitol + 1mM glucose rel. ab	±	sd [%]	sorbitol + 60mM glucose rel. ab	±	sd [%]	Description (conserved domains)
**Plasma membrane**											
201546	4	0.984	±	0.008	1.141	±	0.039	0.353	±	0.262	-
1145073;1115054;1186115	9;9;10	0.677	±	0.152	0.908	±	0.260	0.763	±	0.063	ATPase, P-type, K/Mg/Cd/Cu/Zn/Na/Ca/Na/H-transporter<br>ATPase, P-type cation-transporter, N-terminal<br>Plasma-membrane proton-efflux P-type ATPase<br>H+ transporting ATPase, proton pump<br>E1-E2 ATPase-associated region
1180988	4	0.131	±	0.011	0.151	±	0.011	0.241	±	0.008	Yip1 domain<br>Bacterial surface antigen (D15)
1141113	1	0.116	±	0.030	0.032	±	0.001	0.089	±	0.011	Glycoside hydrolase, catalytic core<br>X8
1093022	11	0.116	±	0.000	0.041	±	0.001	0.080	±	0.013	Amino acid transporter, transmembrane
1142721	3	0.103	±	0.009	0.113	±	0.013	0.060	±	0.002	B-cell receptor-associated 31-like
1133629	6	0.098	±	0.049	0.182	±	0.008	0.238	±	0.141	Formate/nitrite transporter<br>Lipocalin
1084143	1	0.098	±	0.013	0.043	±	0.004	0.056	±	0.001	Tricalbin<br>C2 calcium/lipid-binding region, CaLB<br>C2 calcium-dependent membrane targeting
1113589	11	0.097	±	0.054	0.000	±	0.000	0.061	±	0.009	Rh-like protein/ammonium transporter<br>Blood group Rhesus C/E and D polypeptide<br>Ammonium transporter
1141872	12	0.096	±	0.023	0.072	±	0.008	0.099	±	0.012	Amino acid/polyamine transporter I

1121621	12	0.091	±	0.010	0.119	±	0.048	0.095	±	0.035	Major facilitator superfamily
1105147	14	0.089	±	0.000	0.071	±	0.020	0.117	±	0.012	Major facilitator superfamily MFS-1
											ATP-dependent DNA ligase, conserved site
1107420	4	0.087	±	0.004	0.030	±	0.001	0.073	±	0.001	OST3/OST6
											Thioredoxin-like fold
1141471	6	0.085	±	0.015	0.051	±	0.002	0.108	±	0.004	Protein of unknown function DUF1774, fungi
1143598	10	0.064	±	0.018	0.175	±	0.024	0.000	±	0.000	Major facilitator superfamily
1144791	12	0.062	±	0.012	0.000	±	0.000	0.000	±	0.000	Major facilitator superfamily
1184281	1	0.061	±	0.049	0.037	±	0.003	0.110	±	0.012	Alkaline-phosphatase-like, core domain
											Alkaline phosphatase
1101809	10	0.048	±	0.005	0.020	±	0.003	0.000	±	0.000	Major facilitator superfamily
											General substrate transporter
											Sugar transporter, conserved site
											Sugar transporter
											MFS general substrate transporter
1026385	12	0.047	±	0.004	0.027	±	0.001	0.056	±	0.003	Amino acid/polyamine transporter I
											Amino acid permease-associated region
											Amino acid permease subfamily
209668	2	0.046	±	0.024	0.055	±	0.022	0.082	±	0.011	-
1177845	4	0.041	±	0.032	0.098	±	0.001	0.000	±	0.000	Shr3 amino acid permease chaperone
1128338	9	0.037	±	0.003	0.044	±	0.000	0.065	±	0.008	Major facilitator superfamily MFS-1
1141295	2	0.031	±	0.014	0.033	±	0.010	0.019	±	0.002	GOLD
											emp24/gp25L/p24
1122987	1	0.030	±	0.014	0.042	±	0.012	0.071	±	0.009	Ribophorin I
1148445	9	0.029	±	0.003	0.000		0.000	0.061	±	0.014	Protein of unknown function DUF250
51992	15	0.028	±	0.003	0.034	±	0.004	0.022	±	0.012	Na+/solute symporter
1186700	2	0.027	±	0.003	0.016	±	0.000	0.018	±	0.005	VPS10
											BNR repeat
1142882	11	0.022	±	0.005	0.045	±	0.002	0.000	±	0.000	Major facilitator superfamily
1142864	1	0.022	±	0.010	0.014	±	0.001	0.019	±	0.013	-

1098273	16	0.022	±	0.006	0.027	±	0.001	0.031	±	0.004	Glycosyl transferase, family 48
											BAAT/Acyl-CoA thioester hydrolase C-
44753	1	0.020	±	0.007	0.000	±	0.000	0.000	±	0.000	terminal
1186946	5	0.019	±	0.002	0.000	±	0.000	0.026	±	0.000	Cystinosin/ERS1p repeat
											Mannose-P-dolichol utilization defect 1
											protein
											Protein of unknown function DUF284,
1019516	2	0.018	±	0.001	0.000	±	0.000	0.007	±	0.003	transmembrane eukaryotic
1187492	2	0.018	±	0.004	0.000	±	0.000	0.011	±	0.001	Cytochrome P450, E-class, group IV
1162850	1	0.016	±	0.005	0.000	±	0.000	0.000	±	0.000	Mannose-6-phosphate receptor, binding
1038787	1	0.014	±	0.008	0.017	±	0.001	0.027	±	0.015	Peptidase M28
											Transferrin receptor-like, dimerisation
											Protease-associated PA
1180715	13	0.013	±	0.006	0.003	±	0.000	0.019	±	0.005	Glycoside hydrolase, catalytic core
											Glycosyl transferase, group 1
											Starch synthase catalytic region
											Glycosyl hydrolase, family 13, catalytic
											region
											Glycosyl hydrolase, family 13, subfamily,
											catalytic region
											Cation-dependent mannose-6-phosphate
202429	1	0.013	±	0.001	0.000		0.000	0.011	±	0.002	receptor
											Mannose-6-phosphate receptor, binding
											Phosphatidic acid phosphatase type
1146541	3	0.012	±	0.002	0.012	±	0.002	0.011	±	0.007	2/haloperoxidase
1112865	1	0.012	±	0.001	0.008	±	0.001	0.003	±	0.000	Cytochrome P450, E-class, group IV
											P-II protein urydylation site
											ERG4/ERG24 ergosterol biosynthesis
1101638	9	0.011	±	0.004	0.000	±	0.000	0.000	±	0.000	protein
1188093	9	0.011	±	0.004	0.008	±	0.001	0.008	±	0.003	Globin, subset
											Histone H4

											Oxidoreductase FAD-binding region
											Globin-like
1160579	9	0.011	±	0.001	0.010	±	0.003	0.008	±	0.003	ATPase, P-type, K/Mg/Cd/Cu/Zn/Na/Ca/Na/H-transporter
											ATPase, P-type cation-transporter, C-terminal
											Calcium ATPase
1117429	1	0.010	±	0.003	0.000		0.000	0.005	±	0.000	GOLD
1081653	2	0.010	±	0.001	0.000	±	0.000	0.016	±	0.000	Cytochrome P450, E-class, group IV
											Cytochrome P450
											Legume lectin, beta domain
1141222;1143346;1153587	7;8;6	0.026	±	0.002	0.024	±	0.006	0.029	±	0.005	Chitin synthase
											Chitin synthase N-terminal
1107559	1	0.008	±	0.001	0.000	±	0.000	0.022	±	0.003	Glycoside hydrolase, catalytic core
											Glycolipid anchored surface protein GAS1
1116513	12	0.008	±	0.002	0.000	±	0.000	0.005	±	0.002	Amino acid/polyamine transporter I
											Amino acid permease, fungi
											Amino acid permease, conserved site
1186153	14	0.007	±	0.001	0.003	±	0.001	0.012	±	0.000	ABC transporter, transmembrane region, type 1
41765	11	0.007	±	0.000	0.007	±	0.005	0.009	±	0.001	Protein of unknown function DUF221
1087119	9	0.005	±	0.003	0.005	±	0.001	0.002	±	0.000	ERG4/ERG24 ergosterol biosynthesis protein
											Dienelactone hydrolase
1086577	5	0.004	±	0.001	0.000	±	0.000	0.013	±	0.004	Cleft lip and palate transmembrane 1
1129336	9	0.003	±	0.000	0.005	±	0.001	0.011	±	0.000	TGF-beta receptor, type I/II extracellular region
1125334	5	0.003	±	0.002	0.005	±	0.001	0.000	±	0.000	-
1160087	14	0.003	±	0.001	0.013	±	0.000	0.014	±	0.002	Sterol-sensing 5TM box
											Patched

												Aspartic acid and asparagine hydroxylation site
	1189214	9	0.003	±	0.000	0.000	±	0.000	0.000	±	0.000	Major facilitator superfamily
	1158555	1	0.002	±	0.001	0.000	±	0.000	0.000	±	0.000	-
	1214584	1	0.001	±	0.000	0.002	±	0.000	0.000	±	0.000	Peptidase M28
	1143191	12	0.000	±	0.000	0.000	±	0.000	0.000	±	0.000	Major facilitator superfamily
	1089440	7	0.000	±	0.000	0.012	±	0.001	0.000	±	0.000	Major facilitator superfamily MFS-1
	1104422	14	0.000	±	0.000	0.003	±	0.001	0.000	±	0.000	ABC transporter, transmembrane region, type 1
	1113000	8	0.000	±	0.000	0.001	±	0.000	0.006	±	0.000	Peptidase M28
												Leucine-rich repeat 2
												Leucine-rich repeat, cysteine-containing subtype
	1127869	13	0.000	±	0.000	0.006	±	0.000	0.000	±	0.000	Oligopeptide transporter OPT superfamily
	1145364	3	0.000	±	0.000	0.283	±	0.064	0.000	±	0.000	TB2/DP1 and HVA22 related protein
	1185324	6	0.000	±	0.000	0.045	±	0.023	0.040	±	0.012	Major intrinsic protein
	1185987	11	0.000	±	0.000	0.003	±	0.001	0.002	±	0.000	-
	1188715	2	0.000	±	0.000	0.003	±	0.002	0.000	±	0.000	ABC transporter, transmembrane region, type 1
	1022562	11	0.000	±	0.000	0.000	±	0.000	0.029	±	0.002	Protein of unknown function DUF250
	1099906	5	0.000	±	0.000	0.000	±	0.000	0.002	±	0.000	Protein of unknown function DUF590
	1111630	10	0.000	±	0.000	0.000	±	0.000	0.011	±	0.001	Major facilitator superfamily MFS-1
	1118545	11	0.000	±	0.000	0.000	±	0.000	0.039	±	0.011	TGF-beta receptor, type I/II extracellular region
	1124902	12	0.000	±	0.000	0.000	±	0.000	0.014	±	0.004	Major facilitator superfamily MFS-1
	1146101	14	0.000	±	0.000	0.000	±	0.000	0.012	±	0.009	Major facilitator superfamily MFS-1
	1156564	8	0.000	±	0.000	0.000	±	0.000	0.000	±	0.000	Glycoside hydrolase, catalytic core
												Amylo-alpha-1,6-glucosidase
												Glycogen debranching enzyme
												Six-hairpin glycosidase-like
												Protein of unknown function DUF221
	1175877	9	0.000	±	0.000	0.000	±	0.000	0.008	±	0.002	-
	1180703	12	0.000	±	0.000	0.000	±	0.000	0.014	±	0.001	Major facilitator superfamily

1187342	13	0.000	±	0.000	0.000	±	0.000	0.003	±	0.001	Glycoside hydrolase, catalytic core
1188840	12	0.000	±	0.000	0.000	±	0.000	0.030	±	0.013	Major facilitator superfamily MFS-1 Nitrate transporter
1168100	1	0.000	±	0.000	0.000	±	0.000	0.013	±	0.003	-
1039419	1	0.000	±	0.000	0.000	±	0.000	0.003	±	0.000	Octicosapeptide/Phox/Bem1p Cystathionine beta-synthase, core
1227231	12	0.000	±	0.000	0.000	±	0.000	0.001	±	0.000	ABC transporter, transmembrane region, type 1
1166284	5	0.000	±	0.000	0.000	±	0.000	0.001	±	0.000	Calcium-binding EF-hand
**Membrane bound mitochondrial**											
1147568	4	1.360	±	0.317	1.372	±	0.292	1.563	±	0.244	Mitochondrial carrier protein
1103448	1	0.455	±	0.120	0.321	±	0.099	0.381	±	0.041	Prohibitin Band 7 protein
1142070	3	0.362	±	0.047	0.633	±	0.150	0.445	±	0.072	-
1146906	1	0.347	±	0.016	0.495	±	0.029	0.247	±	0.007	Ubiquinol-cytochrome C reductase, UQCRX/QCR9-like
1141403	2	0.294	±	0.102	0.564	±	0.078	0.344	±	0.102	-
1145529	1	0.217	±	0.060	0.144	±	0.078	0.093	±	0.013	GRIM-19
1164228	3	0.167	±	0.119	0.167	±	0.036	0.106	±	0.016	-
1179969	1	0.166	±	0.081	0.363	±	0.003	0.192	±	0.070	Protein of unknown function DUF543
1167367	1	0.126	±	0.005	0.050	±	0.011	0.052	±	0.005	Concanavalin A-like lectin/glucanase
1148052	3	0.123	±	0.021	0.083	±	0.025	0.096	±	0.013	Hypoxia induced protein conserved region
1113466	2	0.108	±	0.021	0.082	±	0.010	0.034	±	0.003	Mitochondrial carrier protein Mitochondrial substrate carrier Adenine nucleotide translocator 1
1148385	3	0.073	±	0.011	0.322	±	0.007	0.144	±	0.058	Mitochondrial import inner membrane translocase, subunit Tim17/22
1112854	2	0.066	±	0.017	0.053	±	0.014	0.053	±	0.002	Cytochrome P450, E-class, group I
1104685	3	0.059	±	0.024	0.038	±	0.010	0.054	±	0.009	Protein of unknown function UPF0136, Transmembrane
1146802	3	0.058	±	0.019	0.000	±	0.000	0.008	±	0.004	Mpv17/PMP22

1211786	2	0.031	±	0.017	0.005	±	0.001	0.019	±	0.000	AMP-dependent synthetase and ligase
1143042	1	0.026	±	0.001	0.006	±	0.001	0.021	±	0.004	Ras GTPase
1129330	2	0.021	±	0.000	0.008	±	0.000	0.010	±	0.001	Root hair defective 3 GTP-binding
1187028	4	0.014	±	0.003	0.236	±	0.053	0.158	±	0.003	Protein of unknown function DUF1772, fungi
1126414	1	0.011	±	0.001	0.000	±	0.000	0.013	±	0.002	AAA ATPase, conserved site<br>AAA ATPase, core<br>Peptidase M41, FtsH<br>Peptidase M41
1145516	1	0.010	±	0.001	0.000	±	0.000	0.005	±	0.003	Inorganic phosphate transport PHO88
1141800	1	0.051	±	0.003	0.084	±	0.006	0.059	±	0.003	Mitochondrial substrate carrier
213531	1	0.023	±	0.003	0.021	±	0.004	0.034	±	0.010	Protease inhibitor I4, serpin<br>Tetratricopeptide TPR2<br>Tetratricopeptide TPR-1<br>Tetratricopeptide region<br>Mitochondrial import translocase, subunit Tom70
1186873	5	0.008	±	0.003	0.012	±	0.005	0.000	±	0.000	Insulin-induced
1188148	1	0.005	±	0.002	0.007	±	0.000	0.009	±	0.003	Cytochrome P450, E-class, group IV
213721	3	0.005	±	0.003	0.007	±	0.001	0.005	±	0.003	EXS, C-terminal
1139856	1	0.005	±	0.002	0.000	±	0.000	0.000	±	0.000	Alpha/beta hydrolase fold-1
1182144	1	0.003	±	0.001	0.000	±	0.000	0.000	±	0.000	-
1161772	1	0.003	±	0.001	0.000	±	0.000	0.000	±	0.000	Apc13p<br>Initiation factor 2B related
1102272	1	0.001	±	0.001	0.000	±	0.000	0.001	±	0.000	Sel1-like
1142582	1	0.000	±	0.000	0.202	±	0.040	0.197	±	0.020	-
1183694	3	0.000	±	0.000	0.003	±	0.000	0.000	±	0.000	Fungal transcriptional regulatory protein, N-terminal
1122081	1	0.000	±	0.000	0.000	±	0.000	0.005	±	0.001	NAD(P)-binding<br>Aspartate/homoserine dehydrogenase, NAD-binding<br>Homoserine dehydrogenase, catalytic

1145832	2	0.000 ± 0.000	0.000 ± 0.000	0.037 ± 0.000	Microsomal signal peptidase 25 kDa subunit
1148034	2	0.000 ± 0.000	0.000 ± 0.000	0.036 ± 0.016	-
**Membrane bound ER**					
1147042	1	0.587 ± 0.047	0.833 ± 0.081	0.381 ± 0.064	Cytochrome b5
1030262	10	0.334 ± 0.157	0.381 ± 0.002	0.121 ± 0.014	SecY protein
1148233	1	0.183 ± 0.051	0.149 ± 0.007	0.219 ± 0.010	FAD-binding, type 1
					Flavodoxin-like
					Oxidoreductase FAD/NAD(P)-binding
					Flavoprotein pyridine nucleotide cytochrome reductase
					Flavodoxin/nitric oxide synthase
1141865	2	0.144 ± 0.047	0.057 ± 0.006	0.041 ± 0.006	Cyclopropane-fatty-acyl-phospholipid synthase
1126047	2	0.134 ± 0.047	0.018 ± 0.000	0.046 ± 0.004	Cyclophilin-like
					Surface protein from Gram-positive cocci, anchor region
					Peptidyl-prolyl cis-trans isomerase, cyclophilin-type
1111800	1	0.133 ± 0.011	0.067 ± 0.015	0.059 ± 0.026	Heat shock protein 70
					Heat shock protein Hsp70
1161167	15	0.116 ± 0.038	0.016 ± 0.011	0.113 ± 0.009	Tetrapeptide transporter, OPT1/isp4
					Oligopeptide transporter OPT superfamily
1142917	1	0.099 ± 0.030	0.085 ± 0.026	0.085 ± 0.004	Terpenoid synthase
					Farnesyl-diphosphate farnesyltransferase
					Squalene/phytoene synthase
187424	3	0.085 ± 0.023	0.035 ± 0.007	0.048 ± 0.003	Defender against death DAD protein
1156469	1	0.064 ± 0.002	0.228 ± 0.052	0.142 ± 0.014	ERG2 and sigma1 receptor-like
1022934	7	0.052 ± 0.011	0.040 ± 0.011	0.038 ± 0.003	GNS1/SUR4 membrane protein
1126035	5	0.041 ± 0.010	0.038 ± 0.016	0.045 ± 0.025	Peptidase M48, Ste24p

											Peptidase M, neutral zinc
											metallopeptidases, zinc-binding site
1129295	7	0.038	±	0.014	0.051	±	0.004	0.080	±	0.001	Cytochrome c oxidase, subunit VIa
											Surfeit locus 4-related
											Surfeit locus 4
1141391	5	0.035	±	0.012	0.034	±	0.010	0.028	±	0.001	ER lumen protein retaining receptor
1176663	13	0.032	±	0.002	0.037	±	0.000	0.035	±	0.003	Oligosaccharyl transferase, STT3 subunit
1150065	1	0.030	±	0.004	0.004	±	0.001	0.024	±	0.004	Heat shock protein 70
1122380	10	0.022	±	0.000	0.015	±	0.005	0.019	±	0.004	E1-E2 ATPase-associated region
											ATPase, P-type,
											K/Mg/Cd/Cu/Zn/Na/Ca/Na/H-transporter
											P-type ATPase of unknown pump
											specificity (type V)
											Haloacid dehalogenase-like hydrolase
											Cof protein
											3-oxo-5-alpha-steroid 4-dehydrogenase,
1126550	6	0.020	±	0.006	0.021	±	0.007	0.032	±	0.004	C-terminal
											Phosphotransferase system, HPr serine
											phosphorylation site
1112450	10	0.018	±	0.001	0.014	±	0.002	0.009	±	0.006	Glycosyl transferase, family 39
											MIR motif
											MIR
1185951	17	0.016	±	0.006	0.019	±	0.006	0.052	±	0.006	Endonuclease/exonuclease/phosphatase
											Inositolphosphorylceramide-B
1142806	3	0.016	±	0.000	0.012	±	0.008	0.037	±	0.004	hydroxylase
											Cytochrome b5
											Fatty acid hydroxylase
1184388	7	0.015	±	0.003	0.006	±	0.000	0.009	±	0.002	ABC-2 type transporter
											EGF-like, type 3
											EGF, extracellular

1142025	1	0.015 ± 0.005	0.013 ± 0.001	0.000 ± 0.000	Peptidase S26A, signal peptidase I
					Peptidase S24, S26A, S26B and S26C
					Peptidase S26B, eukaryotic signal peptidase
					Peptidase S24, S26A and S26B, C-terminal
1167021	2	0.013 ± 0.000	0.000 ± 0.000	0.015 ± 0.008	Synaptojanin, N-terminal
1128516	2	0.013 ± 0.002	0.017 ± 0.003	0.009 ± 0.000	Cytochrome P450, E-class, group I
1159100	1	0.013 ± 0.006	0.012 ± 0.003	0.018 ± 0.004	UDP-glucuronosyl/UDP-glucosyltransferase
1151842	3	0.012 ± 0.005	0.032 ± 0.004	0.023 ± 0.010	Short-chain dehydrogenase/reductase SDR
					Glucose/ribitol dehydrogenase
					NAD(P)-binding
1087806	1	0.012 ± 0.001	0.004 ± 0.001	0.021 ± 0.002	Glycosyl transferase, family 15
1164538;1188786	10;11	0.021 ± 0.001	0.000 ± 0.000	0.026 ± 0.002	Major facilitator superfamily MFS-1
1165478	9	0.006 ± 0.001	0.002 ± 0.000	0.008 ± 0.001	ALG6, ALG8 glycosyltransferase
1180885	1	0.006 ± 0.002	0.004 ± 0.001	0.009 ± 0.002	Glycoside hydrolase, catalytic core
					Glycoside hydrolase, family 31
1119913	4	0.006 ± 0.003	0.000 ± 0.000	0.008 ± 0.001	Calcium-binding EF-hand
					Like-Sm ribonucleoprotein-related, core
					Membrane protein, At2g17000, predicted
					Mechanosensitive ion channel MscS
1212687	6	0.005 ± 0.001	0.000 ± 0.000	0.002 ± 0.001	Hydroxymethylglutaryl-CoA reductase, class I/II, substrate-binding
					Hydroxymethylglutaryl-CoA reductase, class I, catalytic
					Ribosomal protein S2
					Hydroxymethylglutaryl-CoA reductase, class I/II, NAD/NADP-binding

											Sterol-sensing 5TM box
											Hydroxymethylglutaryl-CoA reductase, class I/II, catalytic
1105077	5	0.004	±	0.003	0.011	±	0.001	0.005	±	0.001	Spermine synthase
1184634	9	0.003	±	0.000	0.000	±	0.000	0.000	±	0.000	Major facilitator superfamily
1102309	3	0.002	±	0.001	0.005	±	0.001	0.000	±	0.000	Sec63
											Heat shock protein DnaJ, N-terminal
1126795	11	0.000	±	0.000	0.007	±	0.001	0.006	±	0.001	ALG6, ALG8 glycosyltransferase
1141396	2	0.000	±	0.000	0.001	±	0.000	0.016	±	0.007	Sterol desaturase
1141926	12	0.000	±	0.000	0.007	±	0.000	0.000	±	0.000	Tetrapeptide transporter, OPT1/isp4
1164790	2	0.000	±	0.000	0.010	±	0.001	0.000	±	0.000	Translocation protein Sec62, ascomycota
											Translocation protein Sec62
											Protein of unknown function UPF0136,
1038793	4	0.000	±	0.000	0.000	±	0.000	0.014	±	0.004	Transmembrane
1145703	1	0.000	±	0.000	0.000	±	0.000	0.032	±	0.011	Cytochrome b5
1165706	11	0.000	±	0.000	0.000	±	0.000	0.000	±	0.000	Major facilitator superfamily MFS-1
											Flagellar basal body rod protein
1173782	3	0.000	±	0.000	0.000	±	0.000	0.022	±	0.002	Fatty acid desaturase, type 1, core
											Delta-9 acyl-CoA desaturase with haem/steroid binding region
											Fatty acid desaturase, type 1, C-terminal
											Fatty acid desaturase, type 1, N-terminal
1178623	11	0.000	±	0.000	0.000	±	0.000	0.047	±	0.011	Major facilitator superfamily MFS-1
1180615	12	0.000	±	0.000	0.000	±	0.000	0.015	±	0.005	Oligopeptide transporter OPT superfamily
129462	9	0.000	±	0.000	0.000	±	0.000	0.003	±	0.000	Alg9-like mannosyltransferase
**Membrane bound golgi**											
1121885	1	0.095	±	0.005	0.054	±	0.009	0.053	±	0.012	Thioredoxin domain
											Thioredoxin-related
1145709	4	0.035	±	0.016	0.000	±	0.000	0.000		0.000	-

1189177	9	0.025 ± 0.005	0.023 ± 0.000	0.021 ± 0.001	Nonaspanin (TM9SF)
1025773	9	0.022 ± 0.004	0.011 ± 0.000	0.035 ± 0.001	Protein of unknown function DUF250
					UDP-galactose transporter
					Dolichyl-diphosphooligosaccharide-
1134649	1	0.022 ± 0.013	0.000 ± 0.000	0.008 ± 0.005	protein glycosyltransferase 48kDa subunit
1182240	5	0.012 ± 0.009	0.014 ± 0.001	0.000 ± 0.000	SNARE associated Golgi protein
1150734	1	0.009 ± 0.005	0.034 ± 0.000	0.007 ± 0.001	-
1169091	7	0.006 ± 0.001	0.000 ± 0.000	0.000 ± 0.000	Nucleotide-sugar transporter
1163105	2	0.000 ± 0.000	0.004 ± 0.000	0.000 ± 0.000	Cytochrome P450, E-class, group I
1157289	1	0.000 ± 0.000	0.000 ± 0.000	0.000 ± 0.000	Cytochrome P450, E-class, group I
**Membrane bound vacuolar**					
1104971	1	0.041 ± 0.005	0.040 ± 0.003	0.032 ± 0.005	Protein of unknown function DUF1620
					Pyrrolo-quinoline quinone
					WD40 repeat-like
					Quinonprotein alcohol dehydrogenase-
					like
1142522	7	0.039 ± 0.037	0.000 ± 0.000	0.000 ± 0.000	Iron permease FTR1
1167192	6	0.035 ± 0.003	0.131 ± 0.009	0.130 ± 0.013	ATPase, V0/A0 complex, 116-kDa subunit
1122202	13	0.009 ± 0.001	0.000 ± 0.000	0.005 ± 0.003	Major facilitator superfamily MFS-1
					Major facilitator superfamily
					Tetracycline resistance protein, TetB
					ABC transporter, transmembrane region,
1093419	12	0.004 ± 0.002	0.001 ± 0.000	0.001 ± 0.001	type 1
					AAA+ ATPase, core
					ABC transporter-like
					ABC transporter, transmembrane region
1080879	12	0.000 ± 0.000	0.000 ± 0.000	0.020 ± 0.001	Citrate synthase-like, core
					Amino acid/polyamine transporter I
1208596	3	0.000 ± 0.000	0.000 ± 0.000	0.002 ± 0.000	SPX, N-terminal

**Membrane bound lysosomal**											
1100508	7	0.027	±	0.003	0.011	±	0.001	0.010	±	0.007	Transmembrane receptor, eukaryota
**Membrane bound extracellular (secreted)**											
1034818	1	0.009	±	0.002	0.007	±	0.001	0.000	±	0.000	-
1139200	1	0.000	±	0.000	0.031	±	0.009	0.000	±	0.000	-
1145281	1	0.000	±	0.000	0.000	±	0.000	0.145	±	0.084	NADH-ubiquinone oxidoreductase B12 subunit
**Mitochondrial - with targeting sequence**											
1141664	1	0.856	±	0.081	1.836	±	0.108	1.898	±	0.373	Cytochrome c oxidase subunit IV
1101772	1	0.597	±	0.104	0.258	±	0.049	0.356	±	0.056	Concanavalin A-like lectin/glucanase<br>Calreticulin/calnexin<br>Calreticulin/calnexin, P
1098376	1	0.316	±	0.019	0.134	±	0.031	0.195	±	0.045	FAD dependent oxidoreductase<br>FAD-dependent glycerol-3-phosphate dehydrogenase
1147204	1	0.251	±	0.212	0.033	±	0.007	0.027	±	0.009	-
209689	1	0.137	±	0.005	0.236	±	0.021	0.097	±	0.011	-
1146585	4	0.130	±	0.052	0.283	±	0.067	0.039	±	0.007	-
1125366	4	0.054	±	0.007	0.038	±	0.005	0.044	±	0.012	FAD dependent oxidoreductase<br>Aromatic-ring hydroxylase<br>Squalene epoxidase
1143198	1	0.050	±	0.022	0.085	±	0.049	0.055	±	0.016	Protein of unknown function UPF0041
1144353	9	0.034	±	0.001	0.024	±	0.012	0.036	±	0.001	Glycosyl transferase, family 39<br>EGF-like region, conserved site<br>Lipase, active site
1143907	2	0.031	±	0.009	0.035	±	0.004	0.000	±	0.000	Succinate dehydrogenase, cytochrome b subunit<br>Succinate dehydrogenase, cytochrome b556 subunit

1179837	1	0.028 ± 0.005	0.031 ± 0.005	0.042 ± 0.006	Nucleoside phosphatase GDA1/CD39
1176655	10	0.027 ± 0.001	0.000 ± 0.000	0.004 ± 0.001	Glycosyl transferase, family 39
1184291	6	0.025 ± 0.015	0.029 ± 0.007	0.047 ± 0.016	-
1144427	1	0.024 ± 0.011	0.000 ± 0.000	0.000 ± 0.000	-
1146026	1	0.023 ± 0.002	0.031 ± 0.006	0.017 ± 0.002	-
1038150	1	0.016 ± 0.012	0.000 ± 0.000	0.000 ± 0.000	Glycosyl transferase, family 15
1144049	4	0.016 ± 0.006	0.023 ± 0.010	0.008 ± 0.002	-
1142370	2	0.015 ± 0.001	0.012 ± 0.004	0.011 ± 0.001	Mitochondrial import protein Pam17
1084241	1	0.013 ± 0.003	0.000 ± 0.000	0.000 ± 0.000	N-acetylglucosaminyl phosphatidylinositol deacetylase
1158534	1	0.013 ± 0.004	0.000 ± 0.000	0.000 ± 0.000	Thioredoxin-like fold
					Copper chaperone SCO1/SenC
					Synthesis of cytochrome c oxidase, Sco1/Sco2
1226708	1	0.012 ± 0.000	0.000 ± 0.000	0.007 ± 0.000	Glycoside hydrolase, family 47
1090476	4	0.012 ± 0.002	0.011 ± 0.002	0.012 ± 0.000	Der1-like
1162283	2	0.011 ± 0.002	0.015 ± 0.002	0.007 ± 0.001	Protein of unknown function DUF1692
1114338	6	0.008 ± 0.000	0.021 ± 0.002	0.031 ± 0.006	-
1127980	1	0.008 ± 0.005	0.005 ± 0.000	0.009 ± 0.002	FAD-dependent pyridine nucleotide-disulphide oxidoreductase
					Calcium-binding EF-hand
					Pyridine nucleotide-disulphide oxidoreductase, NAD-binding region
1164910	2	0.006 ± 0.000	0.009 ± 0.001	0.000 ± 0.000	-
1174618	6	0.005 ± 0.001	0.000 ± 0.000	0.007 ± 0.001	DEK C terminal
					Myosin head, motor region
					Major intrinsic protein
					Fungal chitin synthase
					RNA polymerase sigma factor 54, interaction
1100035	3	0.002 ± 0.000	0.000 ± 0.000	0.000 ± 0.000	-
1176977	1	0.002 ± 0.000	0.000 ± 0.000	0.000 ± 0.000	-
1164300	17	0.001 ± 0.000	0.000 ± 0.000	0.000 ± 0.000	ABC transporter, transmembrane region, type 1

	1110430	11	0.000 ± 0.000	0.009 ± 0.003	0.006 ± 0.000	Amino acid transporter, transmembrane
	1129829	4	0.000 ± 0.000	0.007 ± 0.003	0.000 ± 0.000	ABC transporter, transmembrane region, type 1
	1142998	1	0.000 ± 0.000	0.077 ± 0.049	0.077 ± 0.047	-
	1144677	1	0.000 ± 0.000	0.131 ± 0.002	0.087 ± 0.001	-
	1156944	7	0.000 ± 0.000	0.016 ± 0.005	0.014 ± 0.002	-
	1148070	4	0.000 ± 0.000	0.000 ± 0.000	0.007 ± 0.004	Fatty acid desaturase, type 1, N-terminal
			±			Fatty acid desaturase, type 1
	1174341	8	0.000 ± 0.000	0.000 ± 0.000	0.012 ± 0.001	Phosphatidate cytidylyltransferase, eukaryota<br>Phosphatidate cytidylyltransferase
	1187508	8	0.000 ± 0.000	0.000 ± 0.000	0.012 ± 0.000	Cytochrome oxidase assembly
	1187906	2	0.000 ± 0.000	0.000 ± 0.000	0.007 ± 0.001	Surfeit locus 1
**Mitochondrial - no targeting sequence**						
	1182982	2	0.016 ± 0.004	0.000 ± 0.000	0.000 ± 0.000	-
	1124805	1	0.009 ± 0.005	0.000 ± 0.000	0.015 ± 0.007	-
**Endoplasmatic reticulum (ER)**						
	1186974	4	0.011 ± 0.007	0.000 ± 0.000	0.000 ± 0.000	-
**Golgi**						
	1141641	1	0.021 ± 0.005	0.000 ± 0.000	0.017 ± 0.004	Glycosyl transferase, family 15
	1126561	1	0.007 ± 0.001	0.017 ± 0.001	0.009 ± 0.001	Target SNARE coiled-coil region<br>Vesicle transport v-SNARE
**Nuclear**						
	1144574	1	0.079 ± 0.019	0.020 ± 0.009	0.088 ± 0.016	Concanavalin A-like lectin/glucanase<br>Legume-like lectin
**Cytoplasmic**						
	1091575	2	0.055 ± 0.001	0.023 ± 0.003	0.030 ± 0.010	-

1161479	1	0.016 ± 0.007	0.007 ± 0.004	0.049 ± 0.008	Amidohydrolase 1
1137546	2	0.005 ± 0.003	0.000 ± 0.000	0.000 ± 0.000	Metal-dependent hydrolase, composite Metallophosphoesterase
**Extracellular (secreted)**					
1135869	1	0.100 ± 0.029	0.076 ± 0.042	0.071 ± 0.038	Ribosomal protein L13
1041322	1	0.012 ± 0.005	0.000 ± 0.000	0.008 ± 0.003	-
1178906	1	0.008 ± 0.004	0.000 ± 0.000	0.000 ± 0.000	-
