# Supplementary material for: Aspergillus niger membrane-associated proteome analysis for the identification of glucose transporters
Source: Biotechnol Biofuels. 2015 Sep 17;8:150. doi: 10.1186/s13068-015-0317-9 (PMC4574540; doi:10.1186/s13068-015-0317-9)
Supplement: Supplementary file 5 — Additional file 5. Relative abundances of identified A. niger proteins grouped according to their location within the cell. [file 13068_2015_317_MOESM5_ESM.pdf]

	sorbitol			sorbitol + 1mM glucose			sorbitol + 60mM glucose		
	rel. ab	±	sd [%]	rel. ab	±	sd [%]	rel. ab	±	sd [%]
Plasma membrane	3.773	±	0.648	4.100	±	0.618	3.482	±	0.786
Membrane bound mitochondrial	4.199	±	1.020	5.279	±	0.952	4.420	±	0.730
Membrane bound ER	2.334	±	0.555	2.287	±	0.295	1.952	±	0.290
Membrane bound golgi	0.225	±	0.058	0.140	±	0.011	0.125	±	0.019
Membrane bound vacuolar	0.128	±	0.048	0.172	±	0.012	0.190	±	0.023
Membrane bound lysosomal	0.027	±	0.003	0.011	±	0.001	0.010	±	0.007
Membrane bound extracellular (secreted)	0.009	±	0.002	0.038	±	0.011	0.145	±	0.084
Mitochondrial - with targeting sequence	2.723	±	0.582	3.389	±	0.453	3.171	±	0.627
Mitochondrial - no targeting sequence	0.025	±	0.009	0	±	0	0.015	±	0.007
Endoplasmatic reticulum (ER)	0.011	±	0.007	0	±	0	0	±	0
Golgi	0.028	±	0.006	0.017	±	0.001	0.026	±	0.006
Nuclear	0.079	±	0.019	0.020	±	0.009	0.088	±	0.016
Cytoplasmic	0.076	±	0.011	0.030	±	0.007	0.079	±	0.018
Extracellular (secreted)	0.119	±	0.038	0.076	±	0.042	0.079	±	0.042
