# Supplementary material for: Aspergillus niger membrane-associated proteome analysis for the identification of glucose transporters
Source: Biotechnol Biofuels. 2015 Sep 17;8:150. doi: 10.1186/s13068-015-0317-9 (PMC4574540; doi:10.1186/s13068-015-0317-9)

Growth of *S. cerevisiae* EBY.VW4000 expressing the *A. niger mstG* and *mstH* genes, at different glucose concentrations

The diagram shows glucose consumption profiles and growth curves of *mstG* and *mstH* expressing transformants growing in minimal medium with different glucose concentrations (2.5 mM, 10 mM and 50 mM). Results presented are the mean of three independent experiments.

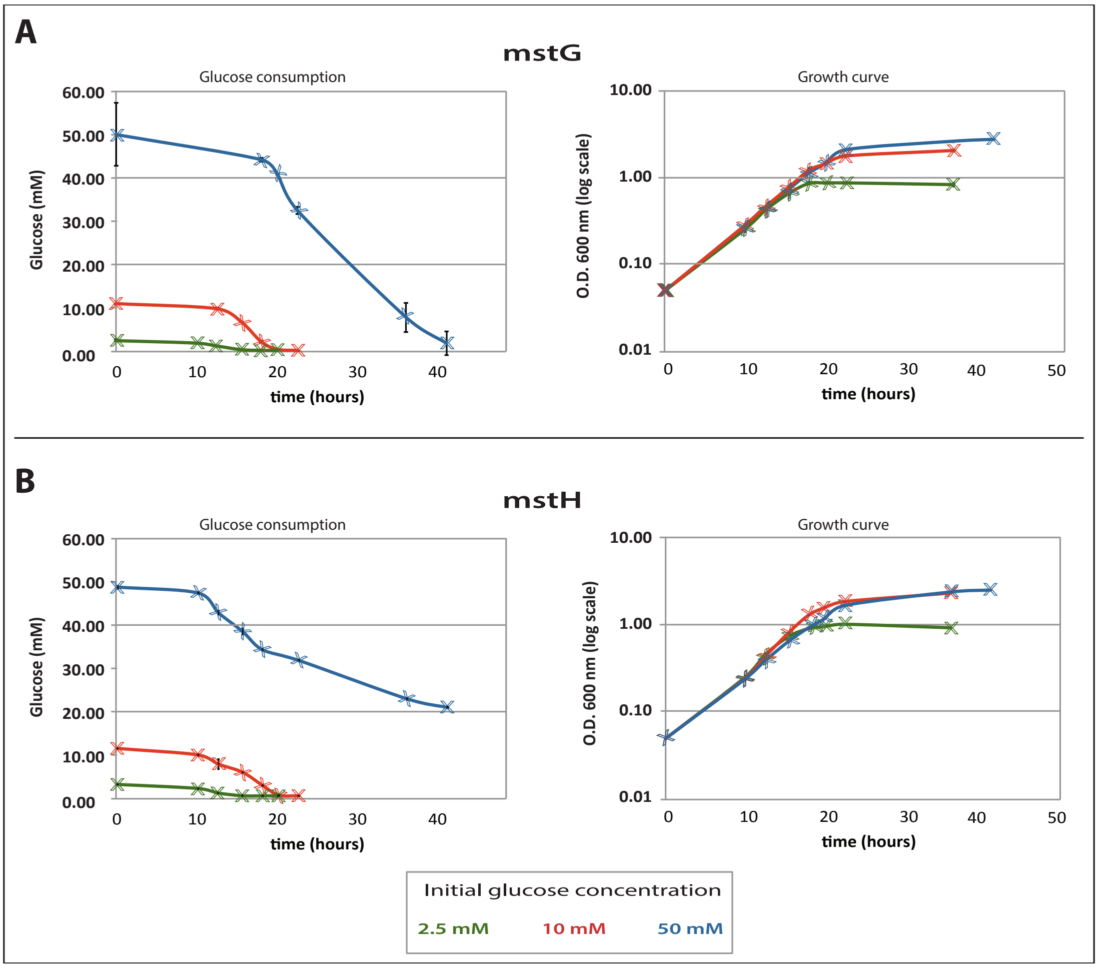

Supplement: Supplementary file 6 — Additional file 6. Growth of S. cerevisiae EBY.VW4000 expressing the A. niger mstG and mstH genes, at different glucose concentrations. [file 13068_2015_317_MOESM6_ESM.pdf]
